# Evaluation of performance of generative large language models for stroke care

**DOI:** 10.1038/s41746-025-01830-9

**Published:** 2025-07-29

**Authors:** John Tayu Lee, Vincent Cheng-Sheng Li, Jia-Jyun Wu, Hsiao-Hui Chen, Sophia Sin-Yu Su, Brian Pin-Hsuan Chang, Richard Lee Lai, Chi-Hung Liu, Chung-Ting Chen, Valis Tanapima, Toby Kai-Bo Shen, Rifat Atun

**Affiliations:** 1 Department of Global Health and Population, Harvard T.H. Chan School of Public Health, Harvard University, Boston, Massachusetts USA; 2 Institute of Health Policy and Management, College of Public Health, National Taiwan University, Taipei, Taiwan; 3 Department of Family Medicine, Taichung Veterans General Hospital, Taichung, Taiwan; 4 Institute of Health Data Analytics and Statistics, National Taiwan University, Taipei, Taiwan; 5 Department of Pediatrics, MacKay Children’s Hospital, Taipei City, Taiwan; 6 Graduate Institute of Health and Biotechnology Law, Taipei Medical University, Taipei City, Taiwan; 7 Graduate Institute of Biomedical Electronics and Bioinformatics, National Taiwan University, Taipei, Taiwan; 8 Stroke Center and Department of Neurology, Chang Gung Memorial Hospital, Linkou Medical Center, Taipei, Taiwan; 9 School of Medicine, Chang Gung University, Taoyuan, Taiwan; 10 School of Medicine, National Yang Ming Chiao Tung University, Taipei, Taiwan; 11 Department of Emergency and Critical Care Medicine, Cheng Hsin General Hospital, Taipei, Taiwan

**Keywords:** Health care, Vascular diseases

## Abstract

Stroke is a leading cause of global morbidity and mortality, disproportionately impacting lower socioeconomic groups. In this study, we evaluated three generative LLMs—GPT, Claude, and Gemini—across four stages of stroke care: prevention, diagnosis, treatment, and rehabilitation. Using three prompt engineering techniques—Zero-Shot Learning (ZSL), Chain of Thought (COT), and Talking Out Your Thoughts (TOT)—we applied each to realistic stroke scenarios. Clinical experts assessed the outputs across five domains: (1) accuracy; (2) hallucinations; (3) specificity; (4) empathy; and (5) actionability, based on clinical competency benchmarks. Overall, the LLMs demonstrated suboptimal performance with inconsistent scores across domains. Each prompt engineering method showed strengths in specific areas: TOT does well in empathy and actionability, COT was strong in structured reasoning during diagnosis, and ZSL provided concise, accurate responses with fewer hallucinations, especially in the Treatment stage. However, none consistently met high clinical standards across all stroke care stages.

## Introduction

Stroke is the second-leading cause of death and the third-leading cause of disability worldwide^1^. Most stroke cases are preventable^2^ and result from impaired blood flow to the brain^3^. Major pathological stroke types comprise ischemic stroke, primary intracerebral hemorrhage, and subarachnoid hemorrhage^4^. According to TOAST classification, ischemic stroke can be further denoted into 5 subtypes: (1) large-artery atherosclerosis, (2) cardioembolism, (3) small-vessel occlusion, (4) stroke of other determined etiology, and (5) stroke of undetermined etiology, which have distinct risk factors, treatments, and outcomes^5^. Socioeconomic factors significantly influence stroke prevalence and outcomes, disproportionately affecting lower socioeconomic groups^6–8^ and creating major disparities. Targeted interventions in stroke prevention, treatment, and management are urgently needed to address these disparities.

Generative Large Language Models (LLMs) are designed to generate text in response to prompts and are widely used by patients and caregivers^9^. Utilizing natural language processing (NLP) technology, LLMs produce human-like text from extensive datasets, providing immediate, accessible health information tailored to specific inquiries. Accessible via the Internet and mobile devices or computers, LLMs can deliver health information in regions with few health professionals or underserved populations. For example, AI-enabled chatbots and telehealth platforms can offer essential medical advice in underserved areas and ‘medical deserts’^10^. This makes LLMs valuable for personalized patient education, improving health literacy, and supporting condition management.

However, there is intense debate about the effectiveness and risks of LLMs for patients and their caregivers. Recent studies on orthopedics and myopia-related eye conditions have shown that while LLMs can provide accurate and relevant information, they may also produce incorrect or misleading content, known as ‘hallucinations’^11,12^. It remains largely unknown whether different prompt engineering techniques can enhance LLM functionality by improving the accuracy, clarity, and empathy of responses for common health conditions like cardiovascular disease.

To date, no studies have investigated how various prompt engineering approaches might improve the performance of generative language models specifically for stroke care. This study examines different prompt types and their effectiveness in providing accurate and useful information for stroke care against current clinical guidelines, medical practices, and public health recommendations.

## Results

The consolidated data across the three generative LLMs—ChatGPT-4o, Claude 3 Sonnet, and Gemini Ultra 1.0—using different prompt engineering techniques (ZSL, COT, TOT) reveal that the overall performance is suboptimal. Most scores fell below the minimum clinical competency threshold of 60, and even when LLMs exceeded this benchmark, the gains were marginal, typically only reaching 60 to 65 at most.

**Fig. 1:**
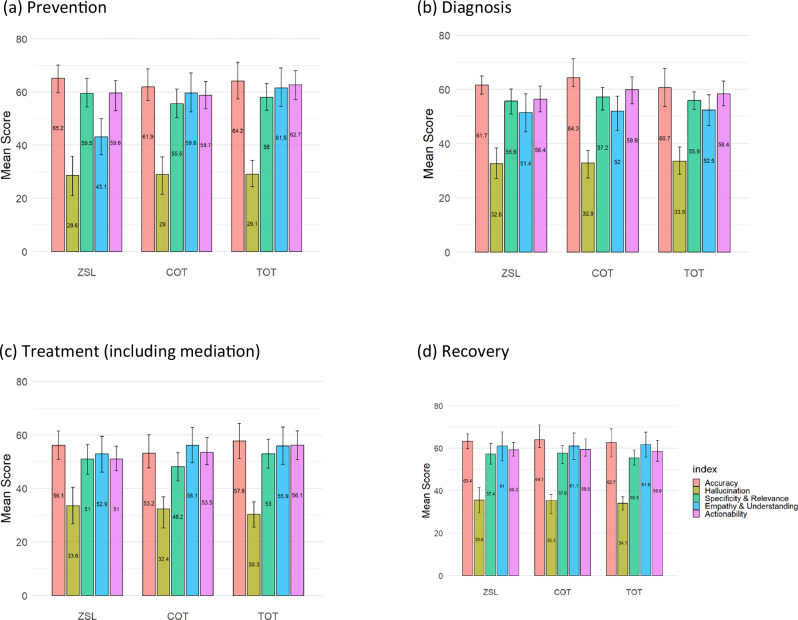
Average performance scores of prompt engineering techniques across stroke care stages. **a** Prevention: Scores (43.1–65.2) show that TOT provides higher empathy (61.5) but lower accuracy and specificity. **b** Diagnosis: Scores (51.4–64.3) reveal a modest advantage of COT in specificity (57.2), with uniformly low hallucination scores (32.6–33.5) and limited actionability. **c** Treatment: Variability (48.2–57.8) reflects challenges in delivering consistent accuracy and actionable guidance. **d** Recovery: Scores (55.5–64.1) indicate that TOT again achieves higher empathy (61.6), with only modest gains in other domains.

As shown in Fig. 1, in the prevention stage, performance scores across all domains (excluding hallucination) ranged from 43.1 to 65.2, with the TOT technique performing better in empathy (61.5) but failing to deliver consistently high scores in accuracy and specificity. In the diagnosis stage, scores fall between 51.4 and 64.3, with COT showing a minor edge in specificity and relevance (57.2), but hallucination scores remain low (in the range of 32.6 to 33.5), indicating a high level of hallucination, and actionability struggles to exceed 59.9.

The treatment stage exhibited the weakest performance: every prompt engineering technique fell below the 60-point clinical competency threshold, with scores between 48.2 and 57.8, underscoring the challenge of generating accurate, actionable advice for this complex phase of care. By contrast, all techniques managed to clear the 60-point cut-off—or came very close—in the prevention, diagnosis, and recovery stages; the gains were modest (typically 60–65) but nevertheless marked an improvement over treatment. Within recovery, for example, scores ranged from 55.5 to 64.1, and TOT showed a relative advantage in empathy (61.6), although domains such as hallucination and actionability did not improve appreciably.

Moreover, according to our results of the mixed-effect model, when assessing the comparative effectiveness of various types of prompt engineering, the effect of each assessor is statistically significant (*p*-value < 0.05). That is, every doctor has their own view about all domains for the 4 stages. In the prevention stage, when considering the domain of empathy and understanding, the score of prompt ZSL is different from COT, and is significantly lower (*p*-value < 0.05). While some prompt techniques (e.g., TOT for empathy, ZSL for hallucination reduction) showed higher mean scores, statistical tests revealed that most differences between techniques were not significant, suggesting a generally similar suboptimal level of performance across prompt engineering strategies in many domains. Therefore, for the following analysis, unless otherwise specified with a *p*-value, pairwise differences among the three prompt techniques or models were not statistically significant (all *p* > 0.05).

This may reflect a performance plateau in prompt effectiveness or limitations in the current evaluation scale’s granularity to detect more nuanced differences in model behavior. Also, these results highlight that while each approach has isolated strengths, the overall scores across all stages suggest significant limitations in delivering adequate, clinically relevant, and actionable outputs for stroke care.

### Average performance of gLLMs by prompt techniques

**Fig. 2:**
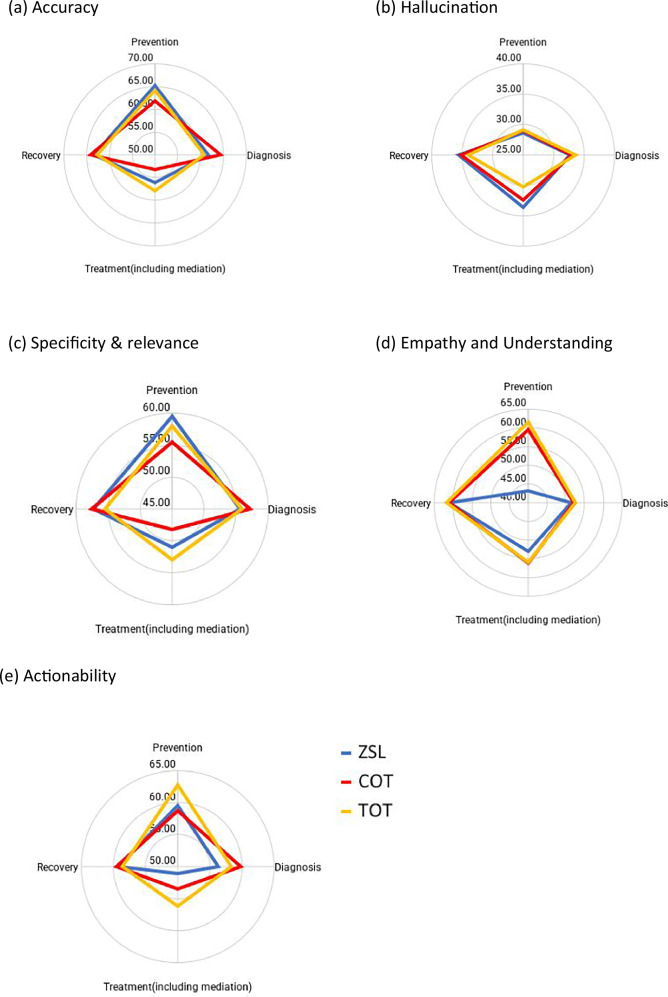
Average performance of prompt engineering techniques across evaluation domains. **a** Accuracy: ZSL does better in prevention, COT in diagnosis (64.33) and recovery, while TOT leads in treatment (57.83), though treatment scores remain generally low. **b** Hallucination: ZSL shows higher scores (fewer hallucinations) in treatment (33.58) and recovery (35.65), whereas TOT performs best in prevention (29.12) and diagnosis (33.55). **c** Specificity and relevance: ZSL is strongest in prevention (59.53), TOT in treatment (52.95), and COT in recovery (57.22). **d** Empathy and understanding: TOT consistently outperforms, notably in prevention (61.55) and recovery (61.65). **e** Actionability: TOT provides the most actionable insights in prevention (62.73) and treatment (56.15), with COT leading in diagnosis (59.88). Overall, these results underscore the complementary strengths of each technique and the importance of tailoring prompt strategies to specific stages of stroke care.

Accuracy domain: As shown in Fig. 2 and Supplementary Table 1, accuracy scores exceeded the total mean in prevention and recovery stages, with ZSL outperforming TOT and COT in prevention and COT leading in recovery. In the diagnosis stage, COT scored highest (64.33), while TOT performed well in the treatment stage (57.83). However, all prompts performed poorly in treatment overall, reflecting challenges in delivering accurate guidance in this phase.

Hallucination domain: Higher scores indicate fewer hallucinations. Among the three prompt techniques, ZSL recorded the highest hallucination scores in treatment (33.58) and recovery (35.65), followed by COT, which achieved slightly lower scores in recovery (35.28) and notably lower in treatment (32.37). In contrast, TOT outperformed the others in prevention and diagnosis, with scores of 29.12 and 33.55, respectively. Although TOT had a marginal advantage in prevention, all methods exhibited low hallucination scores overall, and the differences were minimal. Notably, all three techniques performed best in recovery.

Specificity and relevance domain: Performance varied across stages. ZSL led in prevention (59.53), generating more precise and relevant information. COT outperforms others in diagnosis (57.22), while TOT was strongest in treatment (52.95). Differences among approaches were less pronounced in diagnosis. Overall, while each technique demonstrated strengths in specific stages, none consistently outperformed across all.

Empathy and understanding domain: TOT generally outperformed in empathy, particularly in prevention (61.55) and recovery (61.65). ZSL underperformed in prevention (43.07), falling below the total average. In diagnosis, TOT led (52.45), though differences were minimal. For treatment, COT had a slight edge (56.07). TOT’s ability to convey empathy and understanding made it the most effective approach for patient interactions, especially in prevention and recovery.

Actionability domain: TOT demonstrated relatively strong actionability in prevention (62.73) and treatment (56.15), surpassing ZSL and COT. COT led in diagnosis (59.88), yet this remained just below the 60-point competency threshold, while recovery scores were close across all techniques, but again, the difference was not statistically significant. TOT consistently delivered actionable insights in prevention and treatment, indicating it may be a relatively adequate option in stroke care compared with other prompt techniques.

Overall, TOT performed better in empathy, understanding, and actionability, particularly during the prevention and treatment stages, though it struggled with hallucinations, especially in treatment. ZSL performed relatively well in prevention regarding accuracy, specificity, and relevance, but it lagged in empathy and understanding. Meanwhile, COT demonstrated strengths in diagnosis and recovery accuracy, yet it did not stand out in managing hallucinations or in conveying empathy.

### Comparison of performance across gLLMs

**Fig. 3:**
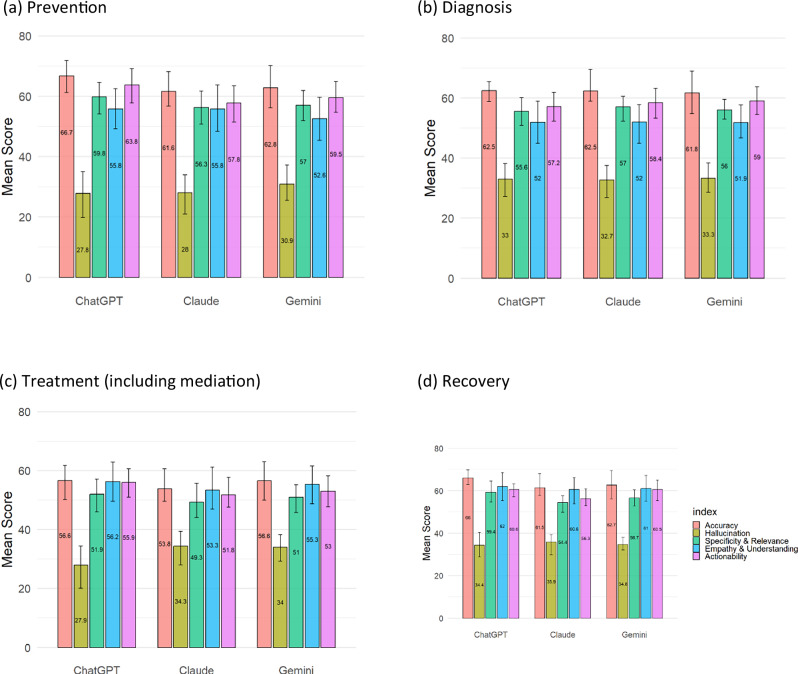
Comparison of the performance of ChatGPT, Claude, and Gemini across stroke care stages. **a** Prevention: ChatGPT attains the highest accuracy (66.73) and actionability (63.77), while Claude scores significantly lower (*p* < 0.05); hallucination scores are lowest for GPT (27.82). **b** Diagnosis: ChatGPT leads in specificity and accuracy, with Gemini performing comparably and Claude trailing slightly. **c** Treatment: Although all models show lower accuracy in this complex phase, ChatGPT again outperforms in accuracy and actionability; both Claude and Gemini have significantly higher (i.e., better) hallucination scores (*p* < 0.05). **d** Recovery: ChatGPT demonstrates superior accuracy (66.05) and actionability (60.58) with the highest specificity, whereas Claude’s performance is significantly lower (*p* < 0.05). Overall, ChatGPT consistently generates more accurate, specific, and actionable responses, despite variations in hallucination and empathy across models.

In this study, we also compared the performance of three generative language models—ChatGPT-4o, Gemini Ultra 1.0, and Claude 3 Sonnet—across five evaluation domains for stroke care across the stages of stroke care cascade. The results are presented in Fig. 3 and Supplementary Table 2.

Additionally, according to our results of the mixed-effect model, almost all models’ effect of each doctor is statistically significant (*p*-value < 0.05). That is, doctors have their own views about all domains for the 4 stages. Please note that, unless otherwise specified with a *p*-value, pairwise differences among the three models were not statistically significant (all *p* > 0.05).

Accuracy domain: GPT generally performs best in terms of accuracy across most stages of stroke care, particularly in stroke prevention (66.73) and recovery (66.05). Claude and Gemini score similarly but slightly lower in accuracy across stages, with Claude trailing the other two models, particularly in treatment and recovery stages. Overall, GPT outperforms Claude and Gemini across most domains, especially in accuracy, specificity, and actionability. Claude and Gemini show comparable but slightly lower scores, with Claude tending to have lower specificity. Gemini provides moderate performance, sometimes approaching GPT but not surpassing it, particularly in empathy and relevance. In the prevention and recovery stage, the score of the model Claude is different from ChatGPT, and is significantly lower (*p*-value < 0.05). This suggests GPT may be the most adequate model for generating accurate, specific, and actionable responses in stroke care.

Hallucination: GPT has the lowest scores in prevention (27.82) and treatment (27.92), indicating it produces the most non-factual outputs in these stages. Gemini generally has higher scores compared to GPT, especially in treatment (33.98), suggesting it generates fewer hallucinations. Claude tends to exhibit the highest score in the recovery stage (35.87), indicating it produces the least hallucinations in this context, while GPT and Gemini show relatively higher levels of non-relevant information. In the treatment (including mediation) stage, the score of the model Claude and Gemini are both different from ChatGPT, and are significantly higher (*p*-value < 0.05).

Specificity and relevance: GPT demonstrates higher specificity and relevance, particularly in prevention (59.83) and recovery (59.38), indicating it provides more focused and relevant information in these stages. Claude consistently scores slightly lower in specificity across stages, suggesting it may produce less specific content. Gemini scores moderately well, often close to GPT, but does not surpass it in any stage. In the recovery stage, the score of the model Claude is different from ChatGPT, and is significantly lower (*p*-value < 0.05).

Empathy and understanding: GPT and Claude perform similarly in most stages, with GPT scoring the highest in recovery (62.05), which may indicate better responsiveness to patient needs in this final care stage. Gemini has a lower empathy score in prevention (52.60) but performs close to the other models in treatment and recovery.

Actionability: GPT consistently provides more actionable insights across the stages, with the highest scores in prevention (63.77) and recovery (60.58). Claude has lower actionability scores, particularly in treatment (51.75) and recovery (56.28), suggesting it may struggle to generate practical advice. Gemini performs comparably to GPT in recovery (60.55), though generally lower across other stages. In the prevention stage, the score of the model Claude is different from ChatGPT, and is significantly lower (*p*-value < 0.05).

## Discussion

The study revealed that while each prompt engineering approach has its strengths, the overall performance of the LLMs across all stages of stroke care indicates significant limitations in delivering clinically relevant and actionable outputs for the public. This suggests that the health information provided by LLMs may not consistently meet clinical standards, urging patients to approach these resources with caution. Non-significant results across many domains suggest that, despite isolated strengths, prompt engineering techniques do not consistently lead to clinically meaningful improvements in LLM outputs.

Overall, TOT emerged as the most effective prompt technique for generating empathetic and actionable responses, especially in prevention and treatment stages. GPT does better across accuracy, specificity, and actionability domains, but has a risk of hallucination. However, persistent limitations in hallucination management and domain-specific relevance underscore the need for improvements across all models and techniques. These findings provide a nuanced understanding of how generative LLMs and prompt engineering methods perform in delivering clinically relevant stroke care information.

Previous studies have highlighted the potential of LLMs to support health information for the public, though their accuracy and usefulness can vary significantly across different applications^13,14^. LLMs may generate more accurate responses for general health information, disease prevention, and prognosis, but they exhibit notable limitations in specialized clinical contexts and treat-specific health inquiries, where errors are more prevalent^15,16^. These limitations raise important concerns regarding the appropriateness of relying on LLMs for health decision-making in urgent or high-stake scenarios. In time-sensitive or clinically complex situations—such as stroke onset, acute medication decisions, or symptom triage—the risk of misinformation or oversimplification could lead to delayed care and inappropriate self-management. Therefore, while LLMs may serve as a useful supplement for general health education, their application in critical or nuanced medical contexts should be approached with caution and supported by professional oversight^16^.

Various prompt engineering strategies have been developed to enhance LLM outputs by providing clear instructions and contextual framing^17^. Techniques like few-shot prompting have consistently been shown to improve accuracy over zero-shot prompting^17^. However, even with these enhancements, LLMs remain prone to generating plausible-sounding but potentially inaccurate rationales, highlighting the ongoing need for human verification and oversight in their clinical use^18^.

Our study distinguishes itself with its extensive methodology: using three LLMs and three prompt engineering techniques across four stages of health treatments, each with different prompt scenarios, and employing a five-domain grading system for LLM performance evaluations. Our comparative analysis of the three major LLMs—Claude, ChatGPT, and Gemini—reveals fundamental differences likely stemming from variations in their training data and design priorities.

The advancement in the generative LLMs presents significant opportunities for enhancing stroke care, particularly in underserved populations. While some LLMs, such as GatorTron, are specifically designed to incorporate real-world healthcare knowledge, concerns remain regarding the clinical applicability of AI in medicine^19,20^. Our study found that the performance of these models was suboptimal, displaying inconsistencies across key domains, including accuracy, hallucinations, specificity, empathy, and actionability when delivering stroke-related health information.

These findings underscore the critical need for the thoughtful integration of LLMs into both clinical practice and patient-facing health information retrieval. In high-risk fields like healthcare, it is essential to ensure that AI systems are transparent, reliable, and guided by human oversight to provide safe, accurate, and context-appropriate patient support^21^. Active involvement of clinical experts is crucial for validating LLM-generated outputs and bridging the gaps between AI-driven content and established clinical guidelines^12,22^. This should include the use of patient-centered prompts that consider individual needs, medical histories, and care goals, ensuring more context-specific guidance^23^. However, integrating patient-specific data into LLMs also introduces significant risks. These include privacy concerns, as sensitive medical data could be exposed through unintended outputs, and the potential for biased or inaccurate responses if the training data is not representative or well-curated^24^. Moreover, this integration raises critical ethical and legal challenges, including the risk of data breaches and violations of patient confidentiality. To address these concerns, robust data protection measures, clear guidelines on the appropriate use of patient data, and ongoing oversight by clinical experts are essential^25^.

There are several important caveats to our study. First, the use of a Likert rating scale introduces an element of subjectivity, as individual evaluators may interpret scores differently (e.g., 50 versus 90), even though our criteria were aligned with the medical doctor qualification exam, which uses a baseline score of 60. This variation in scoring can arise from differences in clinical judgment and personal experience, potentially affecting the consistency of the evaluations. Additionally, assessing empathy remains inherently subjective, as it depends not only on the clarity and tone of the model’s responses but also on the personal interpretation of the evaluator. While we attempted to mitigate this by providing detailed scoring rubrics, more discrete and standardized assessment frameworks might further reduce this variation in future studies. Moreover, our evaluation focused exclusively on stroke care, which, while clinically important, represents only one domain of medical practice. The performance of LLMs in other specialized fields, such as oncology or rare disease management, may differ significantly, and our findings may not generalize to those contexts. Finally, our study was conducted using prompts in English, which may limit the applicability of our findings to non-English clinical settings, where language nuances and cultural context can significantly impact communication quality and patient understanding.

In conclusion, our study highlights the current limitations of LLMs for clinical support. While LLMs offer promising applications in healthcare, the risks of inaccurate or biased outputs underscore the critical need for robust safeguards in their clinical use. Engaging clinical experts in evaluation, as shown in our study, is key to ensuring safety. Future research should continue advancing medical area-specific LLMs while also educating patients on how to frame effective prompts, ensuring that AI can deliver safe, accurate, and equitable healthcare at scale.

## Methods

We evaluated the effectiveness of three LLMs (ChatGPT-4o, Gemini Ultra 1.0, and Claude 3 Sonnet) across four stages of stroke care: (1) prevention, (2) diagnosis, (3) treatment and medication, and (4) recovery and rehabilitation. Prompts were tailored for each stage, including personalized lifestyle recommendations, symptom-checking guidance, treatment suggestions, and recovery exercises based on clinical guidelines and expert input (see Table 1).

**Table 1:** Scenarios used for different stages of stroke care continuum

Stage	Scenario description
Prevention	John, a 50-year-old man, recently learned that his father had a stroke, prompting him to reassess his own health and lifestyle choices. Determined to reduce his risk of having a stroke, John decides to take proactive measures to protect his health.
Diagnosis	While enjoying his morning coffee, John suddenly notices that something feels off. He struggles to lift his left arm to take a sip, and his speech becomes slurred as he tries to ask his wife for help. Unfortunately, she has gone on a morning run, leaving him alone at home.
Treatment and medication	John, a 50-year-old man, is being transported to the hospital for a confirmed stroke. He is experiencing marked weakness on his right side. Anxious, he wants to know how long his recovery might take.
Rehabilitation	Although making a steady recovery, John continues to feel numbness and pain in the affected areas of his body. As a former swimmer, he is concerned about his ability to regain his prior level of physical fitness.

A unique feature of LLMs is that the quality of their responses depends on how questions are framed. To investigate this, we further assessed their effectiveness using three specific and commonly used prompt engineering methods: (1) Zero-shot learning (ZSL), a machine learning approach in which the model provides answers without prior examples, recognizing patterns from minimal data—often just one example per class—by utilizing prior knowledge from training data and similarity metrics to make predictions; (2) Chain of Thought (COT), which adopts a sequential and local progression through the process of generating ideas, involving clear reasoning steps that distinguish it from other prompt engineering methods by guiding the model through step-by-step logic; and (3) Talk Out Your Thoughts (TOT), which involves verbalizing ideas and reasoning processes through conversational, exploratory prompts with unstructured questions connecting related concepts.

To ensure clinical relevance, we characterized the typical clinical presentation of a stroke patient across the care continuum. The stroke-related inquiries posed to the LLMs were based on the most common questions asked by patients in clinical settings—five questions spanning four stages of stroke care. These inquiries were developed in consultation with clinical experts, and the accompanying scenarios were used exclusively to craft prompts with a more realistic, patient-oriented tone. Details of these scenarios are provided in Table 1.

### Generative LLM platforms

We used the latest free versions of three major LLMs (retrieved May 20, 2024): ChatGPT-4o, Gemini Ultra 1.0, and Claude 3 Sonnet. Each platform offers distinct strengths—Gemini for multilingual support, Claude for user-friendliness, and GPT for comprehensive training data. Prompt questions and responses are provided in Supplementary Table 3.

### Validation methods

The LLM responses were evaluated by four senior medical doctors specializing in stroke care. The clinician assessors—C.H.L., C.T.C., V.T., and J.J.W.—bring diverse expertise: one is a stroke care specialist in surgery, two are experienced emergency physicians managing acute stroke cases, and one is a general practitioner skilled in long-term stroke management. Together, their collective expertise provides a well-rounded clinical perspective.

To align model performance with established clinical competency standards, we used the passing threshold of the medical doctor qualification exam (a score ≥ 60/100) as the minimum acceptable level for generated outputs. The LLM responses were embedded in questionnaires and presented in randomized order across prompt techniques and LLM platforms, so that the evaluators were blinded to the source of each response.

### Evaluation criteria

Performance was assessed in five domains critical to patient care: (1) accuracy (alignment with clinical guidelines^26^), (2) hallucination (frequency of false information), (3) specificity and relevance, (4) empathy and understandability, and (5) actionability. Responses were graded on a 1–100 Likert scale based on clinical competency standards (see Table 2).

**Table 2:** Generative LLM performance domain, criteria, and grading scale

Domain	Criteria	Grading (1–100 scale)
Accuracy	Alignment with current clinical guidelines and standard practices	1 = Poor alignment, 100 = Excellent alignment
Hallucination	Frequency of unverifiable or false information	1 = Frequent hallucinations, 100 = No hallucinations
Specificity and relevance	Detail in response, relevance for patient’s own situation	1 = Vague/irrelevant, 100 = Highly specific/relevant
Empathy and understandability	Tone sensitivity to patients’ possible emotional state	1 = Insensitive/confusing, 100 = Empathetic/clear
Actionability	Practical steps and clarity in recommendations	1 = Unclear/no steps, 100 = Clear/practical steps

### Statistical analysis

We employed a mixed-effect model with random intercept to assess the comparative effectiveness of various types of prompt engineering in generating responses related to stroke care, as evaluated by clinicians. This statistical approach enables us to account for the correlation structure of our data, e.g., repeated responses from a clinician. We performed the regression analysis for each outcome separately. Statistical significance was set at a *p*-value of less than 0.05, and all statistical analyses were conducted using the R software (version 4.3.3) with the package “nlme,” an open-source software package. Lastly, we performed comparisons of mean scores between every pair of models (or prompt techniques) within each domain and stage and highlighted the best or worst-performing ones; any difference not accompanied by a *p*-value < 0.05 should be interpreted as non-significant.

### Ethical considerations

The study was approved by the NTU Ethical Review Board (NTU REC-No.: 202406HM036) and used simulated scenarios without real patient data or identifiable information, in full compliance with the Declaration of Helsinki. All human participants provided written informed consent for participation, as confirmed by the NTU Ethical Review Board.

## Supplementary information

Supplementary information

## Data Availability

The statistical analysis code (R version 4.3.3) and LLM response data are available at https://github.com/johntayuleeHEPI/LLM, while the clinicians’ evaluation datasets are available upon reasonable request.

## Code availability

Statistical analysis code in R version 4.3.3 is available for download at: http://github.com/johntayuleeHEPI/LLM.
