# Supplementary material for: Evaluation of performance of generative large language models for stroke care
Source: NPJ Digit Med. 2025 Jul 29;8:481. doi: 10.1038/s41746-025-01830-9 (PMC12307639; doi:10.1038/s41746-025-01830-9)
Supplement: Supplementary file 1 — Supplementary information [file 41746_2025_1830_MOESM1_ESM.pdf]

Supplementary Table 1: Comparing the mean score of three prompt types across four stages with regards to the following five domains

		ZSL	COT	TOT	Average score of accuracy
Accuracy	Prevention	65.17	61.90	64.15	61.27
	Diagnosis	61.68	64.33	60.68	
	Treatment (including mediation)	56.07	53.17	57.83	
	Recovery	63.40	64.08	62.72	
		ZSL	COT	TOT	Average score of hallucination
Hallucination	Prevention	28.62	28.98	29.12	32.25
	Diagnosis	32.60	32.87	33.55	
	Treatment (including mediation)	33.58	32.37	30.28	
	Recovery	35.65	35.28	34.07	
		ZSL	COT	TOT	Average score of specificity and relevance
Specificity and Relevance	Prevention	59.53	55.55	58.03	55.37
	Diagnosis	55.65	57.22	55.85	
	Treatment (including mediation)	50.97	48.22	52.95	
	Recovery	57.35	57.63	55.47	
		ZSL	COT	TOT	Average score of empathy and understanding
Empathy and Understanding	Prevention	43.07*	59.60	61.55	55.73
	Diagnosis	51.42	52.03	52.45	
	Treatment (including mediation)	52.90	56.07	55.88	
	Recovery	61.00	61.10	61.65	
		ZSL	COT	TOT	Average score of actionability
Actionability	Prevention	59.58	58.73	62.73	57.81
	Diagnosis	56.38	59.88	58.35	
	Treatment (including mediation)	51.02	53.47	56.15	
	Recovery	59.25	59.52	58.65	

*Significant difference, $p < 0.05$.

Supplementary Table 2: Comparing the mean score of three generative language models across four stages with regards to the following five domains

		ChatGPT	Claude	Gemini	Average score of accuracy
Accuracy	Prevention	66.73*	61.65	62.83	61.27
	Diagnosis	62.48	62.45	61.77	
	Treatment (including mediation)	56.65	53.83	56.58	
	Recovery	66.05*	61.45	62.70	
		ChatGPT	Claude	Gemini	Average score of hallucination
Hallucination	Prevention	27.82	28.00	30.90	32.25
	Diagnosis	33.03	32.68	33.30	
	Treatment (including mediation)	27.92*	34.33	33.98	
	Recovery	34.38	35.87	34.75	
		ChatGPT	Claude	Gemini	Average score of specificity and relevance
Specificity and Relevance	Prevention	59.83	56.27	57.02	55.37
	Diagnosis	55.63	57.05	56.03	
	Treatment (including mediation)	51.92	49.27	50.95	
	Recovery	59.38*	54.40	56.67	
		ChatGPT	Claude	Gemini	Average score of empathy and understanding
Empathy and Understanding	Prevention	55.82	55.80	52.60	55.73
	Diagnosis	51.98	52.03	51.88	
	Treatment (including mediation)	56.3	53.3	55.3	
	Recovery	62.1	60.7	61.1	
		ChatGPT	Claude	Gemini	Average score of actionability
Actionability	Prevention	63.77*	57.78	59.50	57.81
	Diagnosis	57.17	58.43	59.02	
	Treatment (including mediation)	55.93	51.75	52.95	
	Recovery	60.58	56.28	60.55	

*Significant difference, $p < 0.05$.

Supplementary Table 3: Prompt questions and responses across gLLMs

GPT responses:

-Prevention

One Shot	Responses
What are the main causes of stroke?	Strokes occur when the blood supply to a part of the brain is interrupted or reduced, preventing brain tissue from getting oxygen and nutrients. The main causes of stroke can be categorized into two types: ischemic strokes and hemorrhagic strokes.<br><br>**Ischemic Stroke**<br><br>This is the most common type of stroke, accounting for about 87% of all strokes. It occurs when an artery to the brain is blocked or narrowed, often due to:<br><br>1. **Thrombosis:** Formation of a blood clot (thrombus) within one of the brain's arteries. This clot can develop in arteries damaged by atherosclerosis (buildup of fatty deposits on the artery walls).<br>2. **Embolism:** A blood clot or other debris forms away from the brain—commonly in the heart—and is swept through the bloodstream to lodge in narrower brain arteries. This can be caused by conditions such as atrial fibrillation, which causes the heart's upper chambers to beat irregularly and more rapidly.<br><br>**Hemorrhagic Stroke**<br><br>This type of stroke occurs when a blood vessel in the brain bursts, causing bleeding (hemorrhage) in or around the brain. Causes include:

	1. **Hypertension (High Blood Pressure):** Chronic high blood pressure can weaken the walls of blood vessels, making them more prone to rupture.<br>2. **Aneurysms:** Aneurysms are weakened spots on the artery walls that can balloon out and burst.<br>3. **Arteriovenous Malformations (AVMs):** These are tangles of abnormal blood vessels connecting arteries and veins, which can rupture.<br>4. **Trauma:** Head injury can cause blood vessels in the brain to break.<br>5. **Blood-thinning Medications:** Overuse or misuse of anticoagulants can increase the risk of bleeding.<br><br>**Transient Ischemic Attack (TIA)**<br><br>Sometimes called a mini-stroke, a TIA is a temporary period of symptoms similar to those of a stroke. A TIA doesn't cause permanent damage and is often caused by a temporary decrease in blood supply to part of the brain. It serves as a warning for a potential future stroke.<br><br>**Risk Factors**<br><br>Certain factors can increase the risk of stroke, including:<br><br>● **Unmodifiable Risk Factors:**<br>○ Age: Risk increases with age.<br>○ Sex: Men have a slightly higher risk, although women tend to have strokes at older ages and are more likely to die of stroke.<br>○ Family history: A family history of stroke or TIA.<br>● **Modifiable Risk Factors:**<br>○ Hypertension (High Blood Pressure)<br>○ Diabetes<br>○ High cholesterol

	◦ Smoking<br>◦ Obesity<br>◦ Physical inactivity<br>◦ Unhealthy diet<br>◦ Excessive alcohol intake<br>◦ Heart diseases (e.g., coronary artery disease, atrial fibrillation)<br><br>Addressing these risk factors through lifestyle changes, medications, and regular medical check-ups can significantly reduce the likelihood of a stroke.
Will I have stroke if my parents had one?	Having parents who have had a stroke does increase your risk of experiencing a stroke yourself, but it doesn't guarantee that you will have one. The increased risk is due to a combination of genetic factors and shared lifestyle and environmental factors. Here are some key points to consider:<br><br>**Genetic Factors**<br><br>1. **Family History**: If one or both of your parents had a stroke, especially at a young age, your risk is higher due to the genetic predisposition to conditions like high blood pressure, high cholesterol, and diabetes.<br>2. **Inherited Conditions**: Certain inherited conditions, such as some forms of cardiovascular disease, can also increase your stroke risk.<br><br>**Lifestyle and Environmental Factors**<br><br>1. **Diet and Exercise**: Families often share dietary and exercise habits. Poor diet, lack of exercise, and other unhealthy habits can increase stroke risk.<br>2. **Smoking and Alcohol**: These habits can run in families and significantly increase stroke risk.

	3. **Stress Levels**: High levels of stress can contribute to stroke risk, and stress management styles can be learned within families.<br><br>**Preventive Measures**<br><br>While you can't change your genetics, you can reduce your risk by focusing on modifiable factors:<br><br>1. **Healthy Diet**: Eat a diet rich in fruits, vegetables, whole grains, lean proteins, and healthy fats.<br>2. **Regular Exercise**: Aim for at least 150 minutes of moderate-intensity aerobic activity or 75 minutes of vigorous-intensity activity each week.<br>3. **Avoid Smoking**: If you smoke, seek help to quit. Avoid exposure to secondhand smoke.<br>4. **Limit Alcohol**: Drink alcohol in moderation, if at all.<br>5. **Manage Health Conditions**: Keep conditions like hypertension, diabetes, and high cholesterol under control with the help of your healthcare provider.<br>6. **Regular Check-ups**: Regular health screenings can help catch and manage risk factors early.<br><br>**Conclusion**<br><br>While having a family history of stroke does increase your risk, it doesn't mean a stroke is inevitable. By adopting a healthy lifestyle and managing medical conditions effectively, you can significantly reduce your risk. Regular consultations with your healthcare provider can help tailor a preventive strategy specific to your needs.
What can I do to reduce the risk of stroke?	Reducing the risk of stroke involves adopting a healthy lifestyle and managing medical conditions that contribute to stroke risk. Here are several strategies you can implement:<br><br>**Lifestyle Changes**

1. **Healthy Diet:**
    - **Eat a balanced diet:** Focus on fruits, vegetables, whole grains, lean proteins, and healthy fats.
    - **Reduce salt intake:** High sodium can increase blood pressure, a major stroke risk factor.
    - **Limit sugar and refined carbs:** These can contribute to diabetes and obesity, which are stroke risk factors.
    - **Avoid trans fats and reduce saturated fats:** These can lead to atherosclerosis (plaque buildup in arteries).
2. **Regular Exercise:**
    - **Aim for at least 150 minutes of moderate aerobic activity or 75 minutes of vigorous activity per week.**
    - **Include strength training exercises at least twice a week.**
3. **Maintain a Healthy Weight:**
    - **Losing excess weight:** Reduces strain on the heart and arteries.
4. **Avoid Smoking:**
    - **Quitting smoking:** Reduces stroke risk significantly.
5. **Limit Alcohol Consumption:**
    - **Moderate drinking:** Up to one drink per day for women and two for men.
6. **Manage Stress:**
    - **Stress-reduction techniques:** Such as meditation, yoga, and deep breathing exercises.

Medical Management

1. **Control Blood Pressure:**
    - **Regularly monitor blood pressure:** Aim for a target below 120/80 mmHg if possible.
    - **Medications:** Take antihypertensive medications as prescribed.
2. **Manage Diabetes:**
    - **Keep blood sugar levels under control:** Follow a diabetes management plan that includes diet, exercise, and medication if necessary.

	3. **Manage Cholesterol Levels:**<br>○ **Regular lipid panel checks:** Aim for LDL cholesterol levels below 100 mg/dL.<br>○ **Statins and other cholesterol-lowering medications:** As prescribed by a doctor.<br>4. **Treat Heart Diseases:**<br>○ **Manage atrial fibrillation (AFib):** This condition increases stroke risk and needs treatment with medications or procedures.<br>○ **Other heart conditions:** Follow medical advice to manage any underlying heart issues.<br>5. **Regular Medical Check-ups:**<br>○ **Annual physical exams:** To monitor and manage risk factors.<br><br>**Additional Recommendations**<br><br>● **Know Your Family History:**<br>○ **Be aware of any family history of stroke or heart disease:** Discuss this with your healthcare provider.<br>● **Stay Informed:**<br>○ **Educate yourself about stroke symptoms:** Recognize the signs (F.A.S.T.: Face drooping, Arm weakness, Speech difficulty, Time to call emergency services).<br><br>**Personalized Advice**<br><br>Consulting with a healthcare provider is crucial to create a personalized plan based on your specific risk factors and medical history. They can provide tailored recommendations, prescribe necessary medications, and monitor your progress.
What types of exercise can	Engaging in regular physical exercise can significantly decrease the risk of stroke. Various types of exercise can contribute to this

help decrease the risk of stroke?	benefit, each offering unique advantages. Here are some types of exercise that can help reduce stroke risk:<br><br>1. **Aerobic Exercise**:<br>○ **Examples**: Walking, jogging, swimming, cycling, dancing.<br>○ **Benefits**: Improves cardiovascular health, lowers blood pressure, reduces cholesterol levels, helps maintain a healthy weight, and improves blood flow.<br>2. **Strength Training**:<br>○ **Examples**: Weight lifting, resistance band exercises, bodyweight exercises like squats and push-ups.<br>○ **Benefits**: Enhances muscle strength, improves metabolism, supports weight management, and can help lower blood pressure.<br>3. **Flexibility and Stretching Exercises**:<br>○ **Examples**: Yoga, Pilates, stretching routines.<br>○ **Benefits**: Improves flexibility, reduces muscle tension, enhances joint health, and can help manage stress.<br>4. **Balance and Coordination Exercises**:<br>○ **Examples**: Tai chi, balance drills, standing on one leg.<br>○ **Benefits**: Improves stability, reduces the risk of falls (which can be particularly beneficial for older adults), and supports overall physical function.<br>5. **High-Intensity Interval Training (HIIT)**:<br>○ **Examples**: Short bursts of intense exercise followed by rest or low-intensity exercise, such as sprinting followed by walking.<br>○ **Benefits**: Improves cardiovascular fitness in a shorter amount of time, enhances insulin sensitivity, and supports weight loss.<br>6. **Recreational Sports and Activities**:<br>○ **Examples**: Tennis, basketball, soccer, hiking.<br>○ **Benefits**: Provides cardiovascular benefits, improves overall fitness, and can make exercise more enjoyable, increasing

	adherence to a regular routine.<br><br>**Recommendations for Stroke Prevention:**<br><br>- **Frequency**: Aim for at least 150 minutes of moderate-intensity aerobic activity or 75 minutes of vigorous-intensity activity per week, along with muscle-strengthening activities on two or more days per week.<br>- **Intensity**: Moderate intensity (like brisk walking) or vigorous intensity (like running) depending on fitness level and health status.<br>- **Consistency**: Regular, consistent exercise is key; even small amounts of daily activity can be beneficial.<br>- **Variety**: Incorporate different types of exercise to work various muscle groups and maintain interest.<br>- **Safety**: Start slowly if you are new to exercise or have been inactive, and consult with a healthcare provider before beginning a new exercise program, especially if you have existing health conditions.<br><br>By incorporating a mix of these exercises into your routine, you can effectively lower your risk of stroke and improve your overall health and well-being.
What should I eat to help prevent a stroke?	Preventing a stroke involves adopting a healthy diet that supports cardiovascular health. Here are some dietary recommendations to help reduce your risk of stroke:<br><br>**1. Fruits and Vegetables**<br><br>- **Aim for a variety of colors**: Different colors of fruits and vegetables provide different nutrients and antioxidants.<br>- **Leafy greens**: Spinach, kale, and other leafy greens are high in folate and potassium, which help reduce blood pressure.<br>- **Berries**: Blueberries, strawberries, and other berries are rich in antioxidants that can protect blood vessels.<br><br>**2. Whole Grains**

- **Oats, brown rice, quinoa, and whole wheat**: These grains provide fiber, which helps control blood sugar levels and reduce cholesterol.
- **Avoid refined grains**: Such as white bread and white rice, which can increase blood sugar levels.

3. Lean Proteins

- **Fish**: Especially fatty fish like salmon, mackerel, and sardines, which are high in omega-3 fatty acids that reduce inflammation and lower triglycerides.
- **Poultry**: Skinless chicken and turkey are good sources of protein without the saturated fat found in red meat.
- **Legumes**: Beans, lentils, and peas are excellent sources of protein and fiber.

4. Healthy Fats

- **Nuts and seeds**: Almonds, walnuts, flaxseeds, and chia seeds provide healthy fats and fiber.
- **Olive oil**: A great source of monounsaturated fat, which can help reduce bad cholesterol levels.
- **Avocados**: Provide monounsaturated fat and are also high in potassium.

5. Low-fat Dairy

- **Yogurt, milk, and cheese**: Opt for low-fat or fat-free versions to reduce saturated fat intake.

6. Hydration

- **Water**: Staying hydrated helps maintain proper circulation and reduce the risk of clot formation.

7. Limit Unhealthy Foods

- **Saturated and trans fats**: Found in red meat, butter, fried foods, and many processed foods, these can increase cholesterol and inflammation.
- **Salt (sodium)**: High sodium intake is linked to high blood pressure, so aim to limit it by avoiding processed foods and using herbs for seasoning instead of salt.
- **Added sugars**: Sugary drinks, sweets, and desserts can lead to weight gain and increased risk of diabetes, both of which are risk factors for stroke.

8. Moderate Alcohol Consumption

- **If you drink alcohol**: Do so in moderation. For women, this means up to one drink per day, and for men, up to two drinks per day.

Additional Tips

- **Regular exercise**: Combine a healthy diet with regular physical activity to maintain a healthy weight and improve cardiovascular health.
- **Quit smoking**: Smoking significantly increases the risk of stroke.

By focusing on these dietary principles, you can help reduce your risk of stroke and promote overall cardiovascular health. Always consult with a healthcare provider before making significant changes to your diet or lifestyle, especially if you have existing health conditions.

-Diagnosis

One shot	Responses

I've been having headaches lately. Could it be a stroke?	Experiencing headaches can be concerning, but headaches alone are not usually a sign of a stroke. Strokes typically present with more specific and severe symptoms. However, it's essential to be aware of the signs of a stroke, which are best remembered by the acronym FAST:<br><br>● **F**ace drooping: One side of the face may droop or feel numb.<br>● **A**rm weakness: One arm may feel weak or numb. When both arms are raised, one arm may drift downward.<br>● **S**peech difficulty: Speech may be slurred or hard to understand.<br>● **T**ime to call emergency services: If any of these symptoms are present, call emergency services immediately.<br><br>Other stroke symptoms can include sudden confusion, trouble seeing in one or both eyes, difficulty walking, dizziness, loss of balance or coordination, and a severe headache with no known cause.<br><br>Common causes of headaches include tension, migraines, sinus issues, dehydration, stress, and lack of sleep. If your headaches are severe, persistent, or accompanied by other concerning symptoms, it's important to seek medical advice. Only a healthcare professional can properly diagnose and determine the cause of your headaches.<br><br>If you are worried about your health, it's best to consult with a doctor who can evaluate your symptoms in detail and provide appropriate guidance and treatment.
I've felt numbness in my hand and stiffness in my neck. Could it be a stroke?	Feeling numbness in your hand and stiffness in your neck can be concerning, but they are not necessarily indicative of a stroke. Strokes often present with specific symptoms, including:<br><br>1. Sudden numbness or weakness in the face, arm, or leg, especially on one side of the body.<br>2. Sudden confusion, trouble speaking, or difficulty understanding speech.<br>3. Sudden trouble seeing in one or both eyes.

	4. Sudden trouble walking, dizziness, loss of balance, or lack of coordination.<br>5. Sudden severe headache with no known cause.<br><br>However, numbness in the hand and neck stiffness can be caused by various other conditions, including:<br><br>1. **Cervical Radiculopathy:** A pinched nerve in the neck can cause numbness and tingling in the hand, as well as neck stiffness.<br>2. **Carpal Tunnel Syndrome:** Compression of the median nerve in the wrist can cause numbness in the hand.<br>3. **Tension or Muscle Strain:** Neck stiffness can result from muscle strain or tension.<br>4. **Peripheral Neuropathy:** Conditions like diabetes can cause numbness in the hands.<br><br>Given your symptoms, it’s important to consider the context and any additional symptoms you may have. If you experience any of the classic stroke symptoms mentioned above, seek emergency medical attention immediately.<br><br>Regardless, it's important to consult with a healthcare professional to determine the cause of your symptoms and receive appropriate treatment. They can perform a detailed examination and possibly order imaging studies or other tests to diagnose the issue accurately.
I've been feeling dizzy lately. Could it be a stroke?	Feeling dizzy can be concerning, but it is not always a sign of a stroke. Dizziness can be caused by a variety of factors, ranging from benign conditions to more serious ones. Here are some potential causes of dizziness:<br><br>1. **Inner ear problems**: Issues like vertigo or Meniere’s disease can cause dizziness.<br>2. **Dehydration**: Not drinking enough fluids can lead to dizziness.<br>3. **Low blood sugar**: Especially common in people with diabetes or those who haven't eaten for a while.<br>4. **Low blood pressure**: Sudden drops in blood pressure can cause dizziness.<br>5. **Medications**: Some medications have dizziness as a side effect.<br>6. **Anxiety and stress**: These can also cause feelings of dizziness.

	However, dizziness can be a symptom of a stroke, especially when accompanied by other signs. It's crucial to be aware of the common stroke symptoms, which can be remembered with the acronym FAST:<br><br>● **F**ace drooping: One side of the face may droop or feel numb. Ask the person to smile and see if the smile is uneven.<br>● **A**rm weakness: One arm may be weak or numb. Ask the person to raise both arms and see if one arm drifts downward.<br>● **S**peech difficulty: Speech may be slurred or hard to understand. Ask the person to repeat a simple sentence.<br>● **T**ime to call 911: If someone shows any of these symptoms, even if they go away, call emergency services immediately.<br><br>Other potential stroke symptoms include sudden confusion, trouble seeing in one or both eyes, sudden trouble walking, dizziness, loss of balance or coordination, and a sudden severe headache with no known cause.<br><br>If you're experiencing persistent or severe dizziness, or if it's accompanied by other symptoms like those mentioned above, it's important to seek medical attention promptly. A healthcare professional can evaluate your symptoms and provide the appropriate care.
My blood pressure has been higher lately. Could it be a stroke?	High blood pressure can be a risk factor for a stroke, but it does not necessarily mean that you are having a stroke. Here are some signs and symptoms of a stroke to be aware of:<br><br>1. **Sudden numbness or weakness** in the face, arm, or leg, especially on one side of the body.<br>2. **Sudden confusion**, trouble speaking, or difficulty understanding speech.<br>3. **Sudden trouble seeing** in one or both eyes.<br>4. **Sudden trouble walking**, dizziness, loss of balance, or lack of coordination.<br>5. **Sudden severe headache** with no known cause.<br><br>If you experience any of these symptoms, it is crucial to seek emergency medical attention immediately. It's also important to regularly monitor your blood pressure and consult with your healthcare provider to manage and reduce your risk factors. They can provide

	personalized advice and treatment options.
What symptoms could be a sign of stroke?	Recognizing the symptoms of a stroke quickly is crucial for getting timely medical help. Here are some common signs and symptoms of a stroke:<br><br>**F.A.S.T. Symptoms:**<br><br>● **F**ace drooping: One side of the face droops or feels numb. Ask the person to smile; if their smile is uneven, it might be a stroke.<br>● **A**rm weakness: One arm becomes weak or numb. Ask the person to raise both arms; if one arm drifts downward, it might be a stroke.<br>● **S**peech difficulty: Speech may be slurred, or the person may have difficulty speaking or understanding speech. Ask the person to repeat a simple sentence to check for slurred or strange speech.<br>● **T**ime to call emergency services: If someone shows any of these symptoms, even if they go away, call emergency services immediately and note the time the symptoms first appeared.<br><br>**Other Symptoms:**<br><br>● **Sudden numbness** or weakness in the leg, arm, or face, especially on one side of the body.<br>● **Sudden confusion** or trouble understanding speech.<br>● **Sudden trouble seeing** in one or both eyes.<br>● **Sudden trouble walking**, dizziness, loss of balance, or lack of coordination.<br>● **Sudden severe headache** with no known cause.<br><br>**Less Common Symptoms:**<br><br>● Sudden nausea or vomiting.

	● Brief loss of consciousness or fainting.<br>● Seizures.<br><br>**Additional Considerations:**<br><br>● **Transient Ischemic Attack (TIA)**: Sometimes called a "mini-stroke," TIAs have stroke-like symptoms that last less than 24 hours and are often a warning sign of a future stroke.<br><br>**Immediate Action:**<br><br>● **Act quickly**: Time is critical in treating strokes. The faster the treatment, the better the chances of minimizing brain damage and other complications.<br><br>If you or someone else experiences any of these symptoms, seek medical help immediately.

-Treatment

One shot	Responses
Are there any medications or treatments that can help clear blood plaque blockages?	Yes, there are several medications and treatments that can help manage or reduce blood plaque blockages, which are crucial in the treatment of atherosclerosis. Here are some of the common approaches:<br><br>**Medications**<br><br>1. **Statins**: These drugs lower cholesterol levels in the blood, which can help reduce the formation of plaque in the arteries. Examples include atorvastatin, simvastatin, and rosuvastatin.<br>2. **Antiplatelet agents**: These medications, such as aspirin and clopidogrel, help prevent blood clots that can form in narrowed

	arteries and lead to heart attacks or strokes.<br>3. **ACE inhibitors**: Angiotensin-converting enzyme (ACE) inhibitors, such as lisinopril and enalapril, help lower blood pressure and reduce strain on the heart.<br>4. **Beta-blockers**: These drugs, including metoprolol and atenolol, can help lower blood pressure and heart rate, reducing the heart's workload.<br>5. **Calcium channel blockers**: Medications like amlodipine and diltiazem help relax the blood vessels and lower blood pressure.<br>6. **Niacin**: This vitamin can help lower cholesterol levels and improve overall cholesterol profile.<br>7. **Fibrates**: Drugs such as gemfibrozil and fenofibrate help lower triglyceride levels and can have some beneficial effects on cholesterol.<br><br>**Treatments**<br><br>1. **Lifestyle changes**: This includes a healthy diet low in saturated fats, cholesterol, and sodium, regular physical activity, quitting smoking, and maintaining a healthy weight.<br>2. **Percutaneous coronary intervention (PCI)**: Also known as angioplasty, this procedure involves inserting a catheter with a balloon at its tip into the narrowed part of the artery. The balloon is inflated to widen the artery, and a stent may be placed to keep the artery open.<br>3. **Coronary artery bypass grafting (CABG)**: This surgical procedure involves creating a bypass around the blocked artery using a vessel taken from another part of the body.<br>4. **Carotid endarterectomy**: This is a surgical procedure to remove plaque from the carotid arteries, which supply blood to the brain.<br>5. **Cardiac rehabilitation**: A structured program of exercise and education designed to improve heart health and help manage atherosclerosis.<br><br>It's important to consult with a healthcare provider to determine the best treatment plan based on individual health needs and

	conditions.
What treatments are available for acute stroke?	Acute stroke treatment aims to restore blood flow to the brain and minimize damage. The specific treatment depends on the type of stroke: ischemic or hemorrhagic. Here are the common treatments for each:<br><br>**Ischemic Stroke (caused by a blood clot blocking a blood vessel in the brain)**<br><br>1. **Thrombolytics (Clot-Busting Drugs):**<br>○ **tPA (Tissue Plasminogen Activator):** This medication helps dissolve the clot and is most effective when given within 4.5 hours of symptom onset.<br>2. **Mechanical Thrombectomy:**<br>○ A procedure in which a specialized doctor removes the clot using a device inserted into a blood vessel in the groin and threaded up to the brain. This is typically done within 6 to 24 hours of symptom onset, depending on individual circumstances.<br>3. **Antiplatelet Drugs:**<br>○ **Aspirin:** Given to prevent new clots from forming.<br>4. **Anticoagulants:**<br>○ **Heparin or Warfarin:** These are less commonly used in the acute phase but may be administered to prevent future strokes.<br>5. **Supportive Care:**<br>○ Managing blood pressure, blood sugar levels, and other vital signs.<br><br>**Hemorrhagic Stroke (caused by bleeding in or around the brain)**<br><br>1. **Surgical Intervention:**

- **Aneurysm Clipping or Coiling:** These procedures aim to stop the bleeding from an aneurysm.
- **Surgical Hematoma Evacuation:** Removing the collected blood to reduce pressure on the brain.

2. **Medications:**
   - **Drugs to Lower Blood Pressure:** Reducing the risk of further bleeding.
   - **Medications to Reduce Intracranial Pressure:** Such as mannitol or hypertonic saline.
3. **Endovascular Procedures:**
   - **Aneurysm Coiling:** A minimally invasive procedure to block the aneurysm.
   - **Arteriovenous Malformation (AVM) Repair:** Using techniques like embolization, radiosurgery, or microsurgery to repair abnormal tangles of blood vessels.
4. **Supportive Care:**
   - Maintaining airway, breathing, and circulation.
   - Managing blood pressure and other vital signs.

General Supportive Treatments for Both Types of Stroke:

1. **Rehabilitation:**
   - Physical, occupational, and speech therapy to help patients recover and regain function.
2. **Monitoring and Management of Complications:**
   - Preventing and treating complications such as pneumonia, deep vein thrombosis, and pressure sores.
3. **Lifestyle Modifications:**
   - Encouraging a healthy diet, regular exercise, smoking cessation, and managing underlying conditions like hypertension, diabetes, and hyperlipidemia to prevent future strokes.

Prompt treatment is crucial for improving outcomes and reducing the risk of long-term disability. Always seek immediate medical attention if a stroke is suspected.

What are the potential side effects of medication for stroke?	Medications for stroke treatment and prevention can have a range of potential side effects. These medications can include antiplatelets, anticoagulants, thrombolytics, antihypertensives, and cholesterol-lowering drugs. Here are some common side effects associated with each type:<br><br>**1. Antiplatelets (e.g., Aspirin, Clopidogrel)**<br><br>● **Common Side Effects:**<br>○ Upset stomach<br>○ Heartburn<br>○ Nausea<br>● **Serious Side Effects:**<br>○ Bleeding (including gastrointestinal bleeding and bleeding in the brain)<br>○ Allergic reactions<br><br>**2. Anticoagulants (e.g., Warfarin, Dabigatran, Rivaroxaban)**<br><br>● **Common Side Effects:**<br>○ Bleeding and bruising easily<br>○ Nausea<br>○ Diarrhea<br>● **Serious Side Effects:**<br>○ Severe bleeding<br>○ Hemorrhagic stroke<br>○ Allergic reactions<br>○ Liver damage

	**3. Thrombolytics (e.g., Alteplase)**<br><br>• **Common Side Effects:**<br>○ Bleeding<br>○ Hypotension (low blood pressure)<br>• **Serious Side Effects:**<br>○ Intracranial hemorrhage (bleeding in the brain)<br>○ Major bleeding in other areas of the body<br>○ Allergic reactions<br><br>**4. Antihypertensives (e.g., ACE Inhibitors, Beta Blockers, Calcium Channel Blockers)**<br><br>• **Common Side Effects:**<br>○ Dizziness<br>○ Fatigue<br>○ Headache<br>○ Cough (particularly with ACE inhibitors)<br>• **Serious Side Effects:**<br>○ Severe hypotension<br>○ Kidney dysfunction<br>○ Electrolyte imbalances<br><br>**5. Cholesterol-Lowering Drugs (e.g., Statins)**<br><br>• **Common Side Effects:**<br>○ Muscle pain

	◦ Digestive problems<br>● **Serious Side Effects:**<br>◦ Liver damage<br>◦ Muscle damage (rhabdomyolysis)<br>◦ Increased blood sugar levels (risk of diabetes)<br><br>**Additional Notes:**<br><br>● **Individual Variation:** The side effects experienced can vary significantly from person to person.<br>● **Interaction with Other Medications:** These medications can interact with other drugs, leading to increased side effects or reduced effectiveness.<br>● **Monitoring:** Regular monitoring by healthcare providers is essential to manage and mitigate these side effects effectively.<br><br>Always consult with a healthcare provider to understand the specific risks and benefits associated with any medication prescribed for stroke.
Is it possible for the brain-damaged area to recover?	The ability of a brain-damaged area to recover depends on several factors, including the extent and location of the damage, the individual's age, overall health, and the type of brain injury. Here are some key points:<br><br>1. **Neuroplasticity**: The brain has an inherent ability to reorganize itself by forming new neural connections. This is known as neuroplasticity. It allows healthy parts of the brain to take over functions from the damaged areas to some extent.<br>2. **Severity of Damage**: Minor injuries might heal more completely than severe ones. In cases of mild traumatic brain injury (TBI) or minor strokes, many patients experience significant recovery over time.<br>3. **Type of Damage**: The cause of the brain damage (e.g., stroke, traumatic injury, tumor, or neurodegenerative disease) can influence the recovery process. For example, recovery after a stroke might involve different mechanisms and outcomes

	compared to recovery after a traumatic injury.<br>4. **Age and Health**: Younger individuals often have a better capacity for recovery due to higher neuroplasticity. Overall health, including the presence of conditions like diabetes or hypertension, can also affect recovery.<br>5. **Rehabilitation**: Intensive rehabilitation, including physical therapy, occupational therapy, and speech therapy, can significantly enhance recovery. The earlier rehabilitation starts, the better the potential outcomes.<br>6. **Support and Environment**: A supportive environment and mental health care can contribute positively to recovery. Emotional support, motivation, and a stimulating environment can all play important roles.<br><br>While complete recovery may not always be possible, many individuals can regain a significant amount of function and lead fulfilling lives post-injury with appropriate medical care and rehabilitation.
What should I expect during surgery?	During surgery, the process typically involves several key steps, and understanding them can help alleviate some anxiety. Here's a general overview:<br><br>**Pre-Operative Phase**<br><br>1. **Pre-Surgery Instructions:** You'll receive specific instructions, such as fasting guidelines and any medications to avoid.<br>2. **Pre-Operative Check-In:** You'll check in at the hospital or surgical center, complete paperwork, and provide any necessary medical information.<br>3. **Preparation:** You'll change into a hospital gown and may have an intravenous (IV) line inserted for medications and fluids. The surgical area may be shaved and cleaned.<br><br>**Anesthesia**<br><br>1. **Consultation with Anesthesiologist:** You'll meet with the anesthesiologist to discuss the type of anesthesia that will be used (local, regional, or general).

2. **Administration of Anesthesia:** You'll be given anesthesia to ensure you don't feel pain during the surgery. This might be through an IV or an inhaled gas.

Intra-Operative Phase

1. **Transportation to Operating Room:** You'll be moved to the operating room and positioned on the operating table.
2. **Surgery:** The surgical team will perform the operation. The length and complexity vary depending on the type of surgery.
3. **Monitoring:** Throughout the surgery, your vital signs (heart rate, blood pressure, oxygen levels) will be closely monitored.

Post-Operative Phase

1. **Recovery Room:** After surgery, you'll be taken to a recovery room where medical staff will monitor your vital signs as the anesthesia wears off.
2. **Pain Management:** You'll receive pain relief medications as needed. Inform the staff if you're experiencing pain.
3. **Observation:** Depending on the surgery, you may need to stay in the hospital for observation, or you might be discharged on the same day.

Aftercare

1. **Post-Surgery Instructions:** You'll receive instructions on how to care for the surgical site, medications to take, and activities to avoid.
2. **Follow-Up Appointments:** These are scheduled to monitor your recovery and remove stitches if necessary.

Common Experiences

- **Grogginess and Nausea:** It's common to feel groggy and possibly nauseous as you wake up from anesthesia.
- **Soreness and Swelling:** Expect some soreness and swelling around the surgical site.

	● **Limited Activity:** You may need to limit physical activity for a period to allow proper healing.<br><br>Every surgery is different, so it's important to ask your surgeon for specific details about what to expect before, during, and after your particular procedure.

-Recovery

One shot	Responses
Can rehabilitation and hyperbaric oxygen therapy help restore brain function after a stroke?	Yes, rehabilitation and hyperbaric oxygen therapy (HBOT) can potentially help restore brain function after a stroke. Here's how they contribute to recovery:<br><br>**Rehabilitation**<br><br>Rehabilitation is a critical component of stroke recovery and typically involves a combination of:<br><br>1. **Physical Therapy**: Helps improve mobility, strength, and coordination. Exercises and activities are designed to regain physical functions.<br>2. **Occupational Therapy**: Focuses on improving daily living skills and adapting to physical limitations. It helps patients relearn tasks such as dressing, eating, and bathing.<br>3. **Speech and Language Therapy**: Aims to improve communication skills, including speaking, understanding, reading, and writing. It can also address swallowing difficulties.<br>4. **Cognitive Rehabilitation**: Works on improving memory, problem-solving skills, and other cognitive functions that may be affected by the stroke.<br><br>**Hyperbaric Oxygen Therapy (HBOT)**

HBOT involves breathing pure oxygen in a pressurized room or chamber. The increased oxygen levels can help with stroke recovery in several ways:

1. **Enhancing Oxygen Supply to the Brain**: HBOT increases the amount of oxygen in the blood, which can help improve oxygen delivery to damaged brain tissues and promote healing.
2. **Reducing Swelling**: It can reduce brain swelling and pressure, which is beneficial in the acute phase of stroke.
3. **Stimulating Neuroplasticity**: HBOT may enhance the brain's ability to reorganize and form new neural connections, which is crucial for recovery.
4. **Supporting Metabolic Functions**: Improved oxygenation can support cellular metabolism and energy production, aiding in tissue repair and recovery.

Evidence and Considerations

- **Rehabilitation**: Extensive research supports the effectiveness of rehabilitation in improving functional outcomes after a stroke. Early and intensive rehabilitation is often recommended.
- **HBOT**: The evidence for HBOT is more mixed. Some studies suggest benefits, particularly in chronic stroke patients, while others show limited or no significant improvement. HBOT is generally considered safe but should be administered under medical supervision.

Conclusion

Combining rehabilitation with HBOT may offer additional benefits for some stroke patients, but the effectiveness can vary. Consulting with healthcare professionals is essential to determine the most appropriate treatment plan based on individual needs and the specifics of the stroke.

<table>
<tr>
<td>Can rehabilitation and intravenous laser irradiation of blood help restore brain function after a stroke?</td>
<td>
Rehabilitation and intravenous laser irradiation of blood (ILIB) are two different approaches that have been explored for aiding recovery after a stroke.
<h3>Rehabilitation</h3>
Rehabilitation is a well-established method for helping stroke survivors regain function. It typically includes physical therapy, occupational therapy, and speech therapy, focusing on:

Physical Therapy: Helps improve movement, strength, and coordination.
Occupational Therapy: Assists with daily activities and regaining independence.
Speech Therapy: Aids in recovering communication skills.

Rehabilitation is crucial in the recovery process, and evidence strongly supports its effectiveness in improving outcomes for stroke patients.
<h3>Intravenous Laser Irradiation of Blood (ILIB)</h3>
ILIB involves the use of low-level laser therapy applied intravenously to irradiate blood. Proponents suggest that ILIB can improve blood circulation, enhance oxygen delivery to tissues, and have anti-inflammatory effects. However, the evidence supporting ILIB for stroke recovery is limited and less conclusive compared to traditional rehabilitation methods. Here are some points to consider:

Mechanism: ILIB is thought to work by stimulating blood cells and improving microcirculation, but the exact mechanisms are not well understood.
Research: There is some preliminary research suggesting potential benefits, but large-scale, high-quality clinical trials are needed to confirm these effects.
Regulation: ILIB is not widely accepted or regulated as a standard treatment for stroke recovery.

</td>
</tr>
</table>

	**Combining Approaches**<br><br>While rehabilitation remains the cornerstone of stroke recovery, some patients and clinicians explore additional therapies, including ILIB. However, it's essential to consult healthcare professionals before combining treatments, as they can provide personalized advice based on the latest evidence and individual patient needs.<br><br>**Conclusion**<br><br>Rehabilitation is a proven and essential component of stroke recovery. ILIB is an emerging therapy with potential benefits, but it requires more research to validate its efficacy and safety. Always seek guidance from healthcare providers when considering new or complementary therapies.
Can rehabilitation and transcranial magnetic stimulation aid in restoring brain function after a stroke?	Rehabilitation and transcranial magnetic stimulation (TMS) are promising approaches in aiding the restoration of brain function after a stroke. Here's a brief overview of how each method can help:<br><br>**Rehabilitation**<br><br>1. **Physical Therapy**: Helps regain motor skills and improve muscle strength and coordination through exercises tailored to the patient's needs.<br>2. **Occupational Therapy**: Assists in relearning daily activities and skills such as dressing, cooking, and writing.<br>3. **Speech and Language Therapy**: Aids in recovering communication skills and addressing difficulties with speaking, understanding, reading, and writing.<br>4. **Cognitive Rehabilitation**: Focuses on improving memory, attention, problem-solving, and other cognitive functions affected by the stroke.<br><br>**Transcranial Magnetic Stimulation (TMS)**

1. **Non-Invasive**: TMS is a non-invasive procedure that uses magnetic fields to stimulate nerve cells in the brain.
2. **Motor Cortex Stimulation**: It can be particularly effective in enhancing motor recovery by stimulating the motor cortex, the brain region involved in movement.
3. **Neuroplasticity**: TMS can promote neuroplasticity, the brain's ability to reorganize itself by forming new neural connections, which is crucial for recovery after a stroke.
4. **Improvement in Cognitive Functions**: Research suggests that TMS can also aid in improving cognitive functions such as attention, memory, and language skills in stroke patients.

Combining Rehabilitation and TMS

- **Synergistic Effect**: Combining traditional rehabilitation methods with TMS may have a synergistic effect, potentially enhancing the overall recovery process.
- **Customized Treatment Plans**: Individualized treatment plans that incorporate both approaches can be tailored to address the specific needs and recovery goals of each patient.

Research and Evidence

- **Clinical Studies**: Numerous studies have demonstrated the efficacy of both rehabilitation and TMS in stroke recovery, though ongoing research continues to refine these methods and explore their full potential.
- **Patient Outcomes**: Patients who receive combined treatments often show better functional outcomes and a higher quality of life compared to those receiving standard rehabilitation alone.

Conclusion

While traditional rehabilitation remains a cornerstone of stroke recovery, integrating TMS can provide additional benefits, leading to improved outcomes for many patients. Consulting with healthcare professionals specializing in stroke recovery is essential for

	developing an effective treatment plan.
What assistance does stroke rehabilitation offer?	Stroke rehabilitation provides a range of services and therapies designed to help individuals recover as much function as possible and improve their quality of life after a stroke. The specific assistance offered can vary based on the individual's needs and the severity of the stroke, but generally includes:<br><br>1. **Physical Therapy**: Focuses on improving movement, strength, and coordination. Exercises may help with walking, balance, and other daily activities.<br>2. **Occupational Therapy**: Helps individuals relearn daily activities such as dressing, eating, and bathing. It may also involve adapting the home environment for safety and ease of use.<br>3. **Speech and Language Therapy**: Assists with communication difficulties, including speaking, understanding, reading, and writing. It can also address swallowing problems.<br>4. **Psychological and Emotional Support**: Provides counseling and support to help individuals cope with the emotional impact of a stroke, including depression, anxiety, and changes in self-esteem.<br>5. **Cognitive Rehabilitation**: Focuses on improving memory, problem-solving skills, attention, and other cognitive functions that may be affected by a stroke.<br>6. **Medication Management**: Ensures proper medication use to manage conditions related to stroke recovery and prevent future strokes.<br>7. **Education and Support for Families**: Educates family members about stroke recovery, provides support, and involves them in the rehabilitation process to ensure a supportive environment at home.<br>8. **Adaptive Equipment Training**: Provides training on the use of adaptive equipment and assistive devices to enhance independence in daily activities.<br><br>Stroke rehabilitation is typically a multidisciplinary effort, involving healthcare professionals such as physiatrists, physical therapists, occupational therapists, speech therapists, psychologists, nurses, and social workers working together to create a personalized

	rehabilitation plan.
How long does stroke rehabilitation typically last?	The duration of stroke rehabilitation varies widely depending on the severity of the stroke, the areas of the brain affected, and the individual's overall health and recovery progress. Typically, stroke rehabilitation can be broken down into several phases:<br><br>1. **Acute Phase**: This begins immediately after the stroke, usually within 24-48 hours, and lasts for about 1-2 weeks. During this phase, the focus is on stabilizing the patient and preventing further complications.<br>2. **Subacute Phase**: This phase usually lasts for 2-3 months. Rehabilitation during this period is often more intensive and may occur in an inpatient rehabilitation facility, where patients receive daily therapy.<br>3. **Chronic Phase**: This phase can last from several months to years. Rehabilitation during this time often involves outpatient therapy, home-based exercises, and community-based programs. The focus is on maximizing recovery and improving quality of life.<br><br>**General Timeline:**<br><br>● **Weeks 1-2**: Initial stabilization and early rehabilitation in the hospital.<br>● **Weeks 2-8**: Intensive inpatient rehabilitation if needed.<br>● **Months 3-6**: Transition to outpatient therapy and home exercises.<br>● **Beyond 6 months**: Long-term rehabilitation and maintenance activities, with periodic reassessments.<br><br>Recovery and rehabilitation progress can vary significantly from person to person. Some individuals may regain most of their functions within a few months, while others may continue to see improvements over several years. Consistent therapy and support are crucial for optimizing recovery outcomes.

Claude responses:

-Prevention

One Shot	Responses
What are the main causes of stroke?	The main causes of stroke include:<br><br>1. Ischemic stroke (caused by a blood clot):<br>- Atherosclerosis (buildup of fatty deposits in arteries) narrowing or blocking blood flow to the brain<br>- Blood clots that form elsewhere in the body and travel to the brain<br>- Conditions like atrial fibrillation, which can cause blood clots<br><br>2. Hemorrhagic stroke (caused by bleeding in the brain):<br>- Uncontrolled high blood pressure, which can cause arteries to burst or rupture<br>- Aneurysms (weakened blood vessel walls) that rupture<br>- Blood clotting disorders<br>- Some blood thinning medications<br><br>3. Other risk factors:<br>- Advancing age<br>- Family history of stroke<br>- High cholesterol levels<br>- Diabetes<br>- Smoking<br>- Obesity

	- Excessive alcohol consumption<br>- Lack of physical activity<br>- Use of illicit drugs like cocaine<br><br>Controlling risk factors like high blood pressure, diabetes, high cholesterol, and adopting a healthy lifestyle can help prevent many strokes. Prompt treatment is crucial when stroke symptoms appear.
Will I have stroke if my parents had one?	Having a parent who had a stroke does increase your risk of also having a stroke, but there are many other factors involved as well. Here are a few key points about the connection between parental stroke history and an individual's stroke risk:<br><br>- Stroke risk tends to run in families due to shared genetic factors that influence things like blood pressure, cholesterol levels, and blood clotting.<br><br>- However, having a parent with stroke does not mean you will definitely have one yourself. Lifestyle factors like diet, exercise, smoking, etc. play a major role too.<br><br>- Your risk is higher if your parent had their stroke at a younger age (e.g. before 65).<br><br>- Your risk may be higher still if both your parents had strokes compared to just one parent.<br><br>- Other risk factors like obesity, diabetes, atrial fibrillation, etc. amplify the effects of inheriting genetic predispositions.<br><br>The most important things you can do are to be aware of the increased risk, adopt a heart-healthy lifestyle, get regular medical checkups to monitor risk factors like blood pressure and cholesterol, and work closely with your doctor on stroke prevention strategies tailored to your individual situation. With proper management, having a parental history does not have to mean an

	inevitable stroke.
What can I do to reduce the risk of stroke?	Here are some tips to help reduce your risk of stroke:<br><br>1. Control high blood pressure: High blood pressure is a major risk factor for stroke. Work with your doctor to keep your blood pressure in a healthy range through lifestyle changes like diet and exercise, and medication if needed.<br><br>2. Maintain healthy cholesterol levels: High levels of LDL "bad" cholesterol can clog arteries and raise stroke risk. Eat a diet low in saturated and trans fats, exercise regularly, and take cholesterol medication if prescribed.<br><br>3. Don't smoke: Smoking doubles the risk of ischemic stroke. If you smoke, make a plan to quit as smoking cessation immediately starts reducing your stroke risk.<br><br>4. Exercise regularly: Being physically active helps control weight, cholesterol levels and blood pressure - all factors that impact stroke risk. Aim for 30 minutes of exercise most days.<br><br>5. Eat a healthy diet: A diet rich in fruits, vegetables, whole grains and lean protein while limiting salt, sugar and unhealthy fats can reduce stroke risk factors.<br><br>6. Limit alcohol: Excessive alcohol intake raises blood pressure and stroke risk. Men should have no more than two drinks per day, women one or less.<br><br>7. Control diabetes: Having diabetes increases stroke risk, so following your treatment plan is crucial if you have this condition.<br><br>8. Treat atrial fibrillation: This irregular heartbeat can allow blood clots to form, boosting stroke risk substantially if untreated.

	The more you control and reduce your modifiable risk factors, the better you can prevent strokes. Work closely with your doctor.
What types of exercise can help decrease the risk of stroke?	Several types of exercise can help decrease the risk of stroke by improving cardiovascular health and managing risk factors such as high blood pressure, obesity, and diabetes. Here are some exercises that are particularly beneficial for reducing stroke risk:<br><br>1. Aerobic exercise: Activities that raise your heart rate and keep it elevated for an extended period, such as brisk walking, jogging, cycling, swimming, or dancing, can improve cardiovascular fitness and help manage weight, blood pressure, and cholesterol levels.<br><br>2. Strength training: Resistance exercises, such as weightlifting or bodyweight exercises, can help build muscle strength and increase metabolism, which can contribute to weight management and improve insulin sensitivity.<br><br>3. Yoga and tai chi: These mind-body practices combine physical postures, breathing exercises, and meditation, which can help reduce stress and lower blood pressure, two risk factors for stroke.<br><br>4. High-intensity interval training (HIIT): Short bursts of intense exercise followed by periods of recovery can improve cardiovascular fitness, increase metabolism, and help manage weight and blood sugar levels.<br><br>5. Flexibility exercises: Stretching and activities that improve range of motion, such as yoga or Pilates, can complement other forms of exercise and contribute to overall physical fitness.<br><br>The American Heart Association recommends at least 150 minutes of moderate-intensity aerobic activity or 75 minutes of vigorous aerobic activity per week, along with two or more days of strength training exercises. However, it's essential to consult with a healthcare professional before starting any new exercise program, especially if you have existing medical conditions or risk factors for stroke.

What should I eat to help prevent a stroke?	Here are some dietary recommendations that may help reduce your risk of having a stroke:<br><br>1. Eat plenty of fruits and vegetables: These are rich in antioxidants, vitamins, minerals, and fiber, which can help lower blood pressure and improve overall vascular health.<br><br>2. Choose whole grains: Whole grains like oats, brown rice, and whole wheat are high in fiber and can help regulate blood pressure and cholesterol levels.<br><br>3. Consume fatty fish: Fatty fish like salmon, mackerel, and sardines are high in omega-3 fatty acids, which have anti-inflammatory properties and can improve cholesterol profiles.<br><br>4. Limit sodium: High sodium intake can raise blood pressure, so aim to limit your sodium intake to no more than 2,300 mg per day.<br><br>5. Eat lean proteins: Lean proteins like poultry, legumes, and low-fat dairy products can provide essential nutrients without excess saturated fat.<br><br>6. Use healthy fats: Olive oil, avocados, and nuts are good sources of monounsaturated and polyunsaturated fats, which can help lower cholesterol levels.<br><br>7. Limit red meat and processed meats: These foods are high in saturated fat and sodium, which can increase stroke risk.<br><br>8. Stay hydrated: Drinking plenty of water can help maintain healthy blood pressure levels.<br><br>In addition to a healthy diet, regular exercise, maintaining a healthy weight, and not smoking are also important for reducing stroke

	risk.

-Diagnosis

One shot	Responses
I've been having headaches lately. Could it be a stroke?	There are several potential causes for recurring headaches, and while a stroke is possible, it is relatively uncommon and unlikely to be the cause unless accompanied by other neurological symptoms. Here are a few points to consider regarding headaches and strokes:<br><br>- Most headaches, even severe ones, are usually related to conditions like tension headaches, migraines, sinus issues, or muscle tension rather than a stroke.<br><br>- Stroke headaches are usually described as sudden, severe "thunderclap" headaches unlike anything experienced before. They reach maximum intensity rapidly.<br><br>- Stroke headaches are more likely if accompanied by other neurological symptoms like weakness/numbness on one side of the body, slurred speech, vision changes, dizziness, or confusion.<br><br>- Risk factors for stroke include high blood pressure, diabetes, smoking, obesity, atrial fibrillation and certain other heart conditions.<br><br>However, any new, severe, or changing pattern of headaches should be evaluated by a doctor, especially if accompanied by other concerning symptoms. Your doctor can assess your symptoms, risk factors, and determine if any testing like a CT scan or MRI is needed to rule out a stroke or other serious condition. Most headaches are benign but getting them checked out provides peace of mind.

I've felt numbness in my hand and stiffness in my neck. Could it be a stroke?	The symptoms you describe - numbness in the hand and stiffness in the neck - could potentially be signs of a stroke, but they can also have other causes. Here are a few key points about these symptoms and strokes:<br><br>- Numbness or weakness, especially on one side of the body, can be a warning sign of a stroke. This is because strokes disrupt blood flow to part of the brain, affecting nerve function.<br><br>- Neck pain or stiffness alone is less commonly associated with strokes, but can sometimes occur if the stroke affects the vertebral arteries that run through the neck.<br><br>- Other common stroke symptoms include sudden confusion, trouble speaking, dizziness, loss of balance/coordination, and severe headache.<br><br>- However, numbness and neck pain/stiffness can also be caused by other issues like pinched nerves, arthritis, muscle strain, etc.<br><br>Because strokes require emergency treatment to prevent permanent brain damage, it's very important to err on the side of caution with these symptoms. If the numbness/stiffness is sudden, severe, or accompanied by other neurological symptoms, you should call emergency services immediately. Even if milder, it's a good idea to get evaluated promptly by a doctor to determine the underlying cause and get proper treatment. Don't wait to see if it goes away on its own.
I've been feeling dizzy lately. Could it be a stroke?	There are several possible causes for feeling dizzy, and a stroke is one potential serious cause that should be ruled out. However, dizziness alone does not necessarily mean you are having a stroke. Here are a few points about dizziness and strokes:<br><br>- Sudden dizziness, especially when accompanied by other neurological symptoms like numbness, weakness on one side of the body,

	slurred speech, vision problems, or a severe headache, could signal a stroke. These are signs to seek immediate medical attention.<br><br>- Dizziness that comes in short spells over a period of time is less likely to be caused by a stroke and more likely related to something like vertigo, inner ear issues, low blood pressure, dehydration, or a side effect of medication.<br><br>- Risk factors for stroke include high blood pressure, diabetes, smoking, obesity, atrial fibrillation, and family history of stroke.<br><br>- If the dizziness persists for more than a day or two, it's a good idea to see your doctor to determine the underlying cause and rule out anything serious like a stroke, brain issue, or heart condition.<br><br>The onset, associated symptoms, duration, and your personal risk factors will help determine if your dizziness requires emergency evaluation for stroke or can be assessed in an outpatient setting. When in doubt, it's better to get it checked out promptly. Let your doctor know about the persistent dizziness.
My blood pressure has been higher lately. Could it be a stroke?	Having high blood pressure is a risk factor for stroke, but an elevated blood pressure reading alone does not necessarily mean you are having or about to have a stroke. Here are a few points about high blood pressure and its relation to stroke risk:<br><br>- High blood pressure (hypertension) puts extra strain on the blood vessels, which over time can damage and weaken them, leading to an increased stroke risk.<br><br>- A single high reading is not an immediate stroke danger, but chronically untreated high blood pressure does increase your risk over time.<br><br>- Symptoms of a stroke include sudden numbness/weakness on one side of the body, slurred speech, severe headache, dizziness and

	vision problems.<br><br>- If you are having any of those stroke symptoms, especially with the high BP readings, you should seek immediate medical attention as it could signify a stroke in progress.<br><br>- If no acute stroke symptoms, the high BP may just need medication adjustment or lifestyle changes like diet, exercise, salt reduction to get it back under control.<br><br>The key is monitoring your blood pressure regularly and speaking to your doctor, especially if readings remain elevated over time. Making lifestyle changes and taking medications as prescribed can help control hypertension and reduce the long-term stroke risk it poses. But any sudden stroke symptoms require urgent evaluation.
What symptoms could be a sign of stroke?	Some potential signs and symptoms of a stroke include:<br><br>- Sudden numbness or weakness in the face, arm, or leg, especially on one side of the body<br>- Sudden confusion, trouble speaking or understanding speech<br>- Sudden trouble seeing in one or both eyes<br>- Sudden trouble walking, dizziness, loss of balance or lack of coordination<br>- Sudden severe headache with no known cause<br><br>The acronym F.A.S.T. is useful for recognizing stroke symptoms:<br><br>F - Face drooping<br>A - Arm weakness<br>S - Speech difficulty

	T - Time to call emergency services immediately<br><br>Other possible symptoms include double vision, drowsiness, and nausea or vomiting. It's crucial to act quickly if you notice any signs of a stroke, as prompt treatment can be crucial to preventing brain damage and disability. Calling emergency medical services right away is advised.

-Treatment

One shot	Responses
Are there any medications or treatments that can help clear blood plaque blockages?	Yes, there are several medications and treatments that can help clear or reduce blood plaque blockages in the arteries. Here are some of the main options:<br><br>1. Cholesterol-lowering medications:<br>- Statins (e.g., atorvastatin, simvastatin, rosuvastatin) help lower LDL (bad) cholesterol and can slow the progression or even cause regression of plaque buildup.<br>- Other cholesterol drugs like fibrates, bile acid sequestrants, and PCSK9 inhibitors may also be used.<br><br>2. Antiplatelet medications:<br>- Aspirin helps prevent blood clots from forming on plaque surfaces.<br>- Other antiplatelet drugs like clopidogrel or prasugrel may be prescribed as well.<br><br>3. Invasive procedures:<br>- Angioplasty and stenting: A catheter is used to inflate a balloon to widen the artery and a stent is placed to prop it open.<br>- Atherectomy: Removal of plaque buildup using a catheter with a rotating shaver or laser.

	- Coronary artery bypass surgery: Rerouting blood flow around blocked arteries using grafted vessels.<br><br>4. Lifestyle changes:<br>- Diet low in saturated fats, trans fats, and cholesterol.<br>- Regular exercise to improve circulation.<br>- Smoking cessation.<br>- Weight loss if overweight or obese.<br><br>The choice of treatment depends on the severity of the blockage, risk factors, and the patient's overall health condition. A combination of medications, procedures, and lifestyle changes is often recommended for optimal management of plaque buildup.
What treatments are available for acute stroke?	There are several treatment options available for acute stroke, which is a medical emergency. The main goals of treatment are to restore blood flow to the affected area of the brain, minimize brain damage, and prevent complications. The specific treatment approach depends on the type of stroke (ischemic or hemorrhagic) and the time elapsed since the onset of symptoms.<br><br>For ischemic stroke (caused by a blood clot blocking blood flow to the brain):<br><br>1. Intravenous thrombolysis (IV tPA): This involves administering a clot-busting drug called tissue plasminogen activator (tPA) through an IV within 4.5 hours of symptom onset. This medication helps dissolve the clot and restore blood flow to the affected area of the brain.<br><br>2. Endovascular thrombectomy: This is a minimally invasive procedure performed within 6-24 hours of symptom onset (depending on the specific guidelines). It involves inserting a catheter into the blocked artery and using specialized devices to remove the clot mechanically.

	3. Antiplatelets: Medications like aspirin or clopidogrel may be given to prevent further clot formation.<br><br>For hemorrhagic stroke (caused by bleeding in the brain):<br><br>1. Control of bleeding: Medications or surgical interventions may be used to control the bleeding and reduce the pressure on the brain.<br><br>2. Treatment of underlying causes: If the bleeding is caused by an underlying condition like an aneurysm or arteriovenous malformation (AVM), treatment may involve surgical clipping, coiling, or other procedures to repair the vascular abnormality.<br><br>3. Management of increased intracranial pressure: Medications, drains, or surgical procedures may be used to reduce the pressure on the brain caused by the bleeding.<br><br>In addition to these specific stroke treatments, supportive care is essential, which may include:<br><br>- Airway management and respiratory support<br>- Blood pressure control<br>- Management of blood sugar levels<br>- Prevention and treatment of complications (e.g., seizures, deep vein thrombosis)<br>- Rehabilitation (physical, occupational, and speech therapy)<br><br>Early recognition of stroke symptoms and prompt medical attention are crucial for improving outcomes and minimizing long-term disability.
What are the	The potential side effects of medications used to treat or prevent strokes can vary depending on the specific drug, but some common

potential side effects of medication for stroke?	ones include:<br><br>1. Bleeding risks: Many stroke medications are anticoagulants or antiplatelets, which can increase the risk of bleeding, including gastrointestinal bleeding, nosebleeds, and bruising.<br><br>2. Hypotension (low blood pressure): Some stroke medications, such as ACE inhibitors or angiotensin receptor blockers, can cause a drop in blood pressure, which may lead to dizziness, lightheadedness, or fainting.<br><br>3. Electrolyte imbalances: Diuretics used to control high blood pressure in stroke patients may cause electrolyte disturbances, such as low potassium levels (hypokalemia).<br><br>4. Liver or kidney problems: Certain stroke medications, like statins, can potentially affect liver or kidney function, especially at higher doses or with long-term use.<br><br>5. Headaches: Medications like aspirin or clopidogrel may cause headaches in some individuals.<br><br>6. Gastrointestinal issues: Drugs like aspirin or anticoagulants can irritate the stomach lining, leading to nausea, vomiting, or stomach pain.<br><br>7. Muscle cramps or weakness: Some blood thinners and diuretics may cause muscle cramps or weakness in some patients.<br><br>8. Fatigue or dizziness: Certain stroke medications, particularly those affecting blood pressure or heart rate, may cause fatigue or dizziness.<br><br>It's important to note that not everyone experiences side effects, and the severity can vary. Discussing potential side effects with a

	healthcare professional is crucial, as they can adjust dosages or consider alternative medications if necessary.
Is it possible for the brain-damaged area to recover?	Yes, it is possible for brain-damaged areas to recover to some extent, though the degree of recovery depends on several factors:<br><br>1. Type of brain injury: Traumatic brain injuries like strokes or head trauma tend to have better potential for recovery compared to neurodegenerative diseases like Alzheimer's.<br><br>2. Location of injury: Brain regions like the cerebral cortex involved in higher cognitive functions have more potential for recovery through neuroplasticity compared to deeper structures like the brainstem.<br><br>3. Age: The brain has greater plasticity and ability to reorganize functions in younger individuals compared to older adults.<br><br>4. Time since injury: Most spontaneous recovery occurs within the first 3-6 months after the brain injury, though improvements can continue over years through rehabilitation.<br><br>5. Severity of injury: Mild to moderate injuries have better recovery outlook compared to severe, widespread brain damage.<br><br>The mechanisms of recovery involve:<br>- Undamaged brain areas taking over functions of damaged regions through forming new neural connections.<br>- Regeneration of new neurons and axons in some brain areas like the hippocampus.<br>- Resolving of brain inflammation and edema over time.<br>- Compensatory behavioral strategies learned through rehabilitation therapies.<br><br>With the right rehabilitation approach tailored to the specific injury, many patients can regain varying degrees of lost cognitive, motor, and functional abilities over time. However, significant recovery is less likely with severe, widespread brain injuries.

What should I expect during surgery?	During surgery, here are some of the key things you can expect:<br><br>Before Surgery:<br>- You'll change into a hospital gown and an IV line may be started to provide fluids and medications.<br>- You'll meet with the anesthesiologist to discuss your medical history and the anesthesia plan.<br>- The surgical area will be cleansed and hair may be clipped if needed.<br><br>During Surgery:<br>- You'll be given general anesthesia, which renders you unconscious, or regional anesthesia to numb part of your body.<br>- Medical monitors will track your vital signs like blood pressure and oxygen levels.<br>- The surgeon will make the necessary incision(s) to access the operative area.<br>- Surgical instruments, cameras or robots may be used depending on the type of surgery.<br>- The surgical team will carry out the repair, removal or reconstruction required.<br><br>After Surgery:<br>- You'll be moved to a recovery room where nurses will monitor you as the anesthesia wears off.<br>- You may have a urinary catheter, wound dressings, drains, etc. in place temporarily.<br>- Pain medication will be provided to keep you comfortable.<br>- You'll be given instructions on how to care for the surgical site and any activity restrictions.<br><br>The whole experience can seem daunting, but knowing what to expect can help ease anxiety. Be sure to discuss any specific concerns with your surgeon.

-Recovery

One shot	Responses
Can rehabilitation and hyperbaric oxygen therapy help restore brain function after a stroke?	Yes, rehabilitation and hyperbaric oxygen therapy can potentially help restore brain function after a stroke, to some extent.<br><br>Rehabilitation:<br>Physical, occupational, and speech therapy are crucial parts of post-stroke rehabilitation. These therapies can help retrain the brain to relearn skills that were lost or impaired due to the stroke. Through repetitive exercises and activities, new neural pathways can form in the brain, compensating for the damaged areas. Rehabilitation can improve mobility, strength, coordination, speech, cognition, and ability to perform daily activities.<br><br>Hyperbaric Oxygen Therapy (HBOT):<br>HBOT involves breathing pure oxygen inside a pressurized chamber. This increases the amount of oxygen dissolved in the blood, allowing more oxygen to reach damaged tissues, including the brain.<br><br>Several studies have suggested that HBOT may promote neuroplasticity (the brain's ability to reorganize and form new neural connections) and stimulate the growth of new blood vessels in the damaged areas of the brain after a stroke. It may also help reduce inflammation and oxidative stress, which can contribute to further brain injury.<br><br>While the evidence for HBOT in stroke recovery is still limited, some studies have reported improvements in neurological function, cognitive abilities, and quality of life when HBOT is used as an adjunctive therapy alongside rehabilitation.<br><br>However, it's important to note that the effectiveness of HBOT may depend on factors such as the timing of treatment initiation, the severity of the stroke, and the individual's response to therapy. Additionally, HBOT is generally considered safe when administered by trained professionals, but it may have some risks and side effects.

	Overall, while rehabilitation remains the primary treatment for stroke recovery, the combination of rehabilitation and HBOT may provide additional benefits for some individuals, but more research is still needed to establish its efficacy and optimal protocols.
Can rehabilitation and intravenous laser irradiation of blood help restore brain function after a stroke?	Yes, rehabilitation and intravenous laser irradiation of blood (also known as intravenous laser blood illumination or IVLBI) are potential therapies that may help restore brain function after a stroke. However, their effectiveness and optimal use are still being researched.<br><br>Rehabilitation:<br>Rehabilitation plays a crucial role in helping stroke survivors regain lost functions and relearn skills. Various types of rehabilitation, such as physical therapy, occupational therapy, speech therapy, and cognitive rehabilitation, can help improve mobility, speech, swallowing, and cognitive abilities, respectively.<br><br>Intravenous Laser Irradiation of Blood (IVLBI):<br>IVLBI is an experimental therapy that involves extracting a small amount of blood from the patient, exposing it to low-level laser irradiation, and then reintroducing it back into the patient's bloodstream. The proposed mechanisms of action include:<br><br>1. Improving microcirculation and blood flow<br>2. Reducing inflammation<br>3. Stimulating stem cell mobilization and tissue repair processes<br><br>Several studies have investigated the use of IVLBI in stroke patients, with some promising results reported, such as improvements in neurological function, reduced disability, and enhanced brain perfusion. However, the evidence is still limited, and larger, well-designed clinical trials are needed to establish the efficacy and safety of this therapy.<br><br>It's important to note that stroke rehabilitation and treatment should be guided by healthcare professionals based on the individual's

	specific condition and needs. While IVLBI is an intriguing experimental therapy, its role in stroke recovery is still being explored, and it should be considered complementary to established rehabilitation practices and conventional stroke treatments.
Can rehabilitation and transcranial magnetic stimulation aid in restoring brain function after a stroke?	Yes, rehabilitation and transcranial magnetic stimulation (TMS) can potentially aid in restoring brain function after a stroke. Here's an overview of how these approaches can help:<br><br>1. Rehabilitation:<br>- Physical therapy: Exercises and activities that target improving motor functions, strength, coordination, and mobility can help rewire neural pathways and promote brain plasticity.<br>- Occupational therapy: Training in daily living activities and adaptive techniques can improve functional independence and encourage neural reorganization.<br>- Speech and language therapy: For individuals with communication difficulties, speech therapy can help retrain language skills and facilitate brain reorganization.<br><br>2. Transcranial Magnetic Stimulation (TMS):<br>- TMS is a non-invasive technique that uses magnetic fields to stimulate specific regions of the brain.<br>- In stroke rehabilitation, TMS is often used to modulate cortical excitability and facilitate neuroplasticity.<br>- Repetitive TMS (rTMS) can be applied to the affected brain region or its contralateral counterpart to enhance neural plasticity and promote functional recovery.<br>- TMS can be combined with rehabilitation therapies to potentially augment the therapeutic effects.<br><br>The brain has a remarkable ability to reorganize and rewire neural connections, a process known as neuroplasticity. Rehabilitation therapies and TMS aim to harness and enhance this neuroplastic potential by providing targeted stimulation and practice, which can facilitate the formation of new neural pathways and improve functional recovery after a stroke.

	It's important to note that the effectiveness of these approaches may vary depending on factors such as the severity and location of the stroke, the individual's age, and the timing of intervention. Rehabilitation and TMS are often used in combination with other therapies, such as medications or constraint-induced movement therapy, for comprehensive stroke management and recovery.
What assistance does stroke rehabilitation offer?	Stroke rehabilitation plays a crucial role in helping stroke survivors regain functionality, independence, and quality of life. It involves a comprehensive and coordinated approach to address the various physical, cognitive, emotional, and social challenges that individuals may face after a stroke. Here are some key aspects of stroke rehabilitation:<br><br>1. Physical therapy: Physical therapists work with stroke survivors to improve their mobility, balance, strength, and coordination. They design individualized exercises and activities to enhance motor function, gait training, and relearning everyday movements.<br><br>2. Occupational therapy: Occupational therapists focus on helping stroke survivors relearn and adapt to the activities of daily living (ADLs) such as dressing, grooming, eating, and performing household tasks. They may use adaptive equipment, environmental modifications, and task-specific training to promote independence and safety.<br><br>3. Speech and language therapy: Speech-language pathologists assist stroke survivors with communication difficulties, such as aphasia (impaired language abilities), dysarthria (slurred speech), and cognitive-communication disorders. They work on improving speech, language comprehension, reading, writing, and cognitive skills.<br><br>4. Cognitive rehabilitation: Cognitive therapists address cognitive impairments that may result from a stroke, including memory, attention, problem-solving, and executive functioning deficits. They utilize strategies, exercises, and techniques to improve cognitive abilities and compensate for cognitive challenges.<br><br>5. Psychological support: Stroke rehabilitation often includes counseling and support services to help individuals and their families cope with the emotional and psychological impacts of stroke, such as depression, anxiety, and adjustment issues.

	6. Education and caregiver training: Rehabilitation teams provide education and training to stroke survivors and their caregivers, equipping them with knowledge and skills to manage various aspects of stroke recovery, including medication management, exercise programs, and home safety measures.<br><br>7. Assistive technology and adaptive equipment: Rehabilitation specialists may recommend and train stroke survivors in the use of assistive devices, such as canes, walkers, wheelchairs, or specialized communication aids, to promote independence and improve functional abilities.<br><br>Stroke rehabilitation is typically provided by a multidisciplinary team of healthcare professionals, including physicians, nurses, therapists, psychologists, and social workers. The goal is to maximize the individual's potential for recovery, improve their quality of life, and facilitate their reintegration into the community.
How long does stroke rehabilitation typically last?	The duration of stroke rehabilitation can vary significantly depending on several factors, including the severity of the stroke, the individual's age, overall health condition, and response to therapy. However, here is a general overview of how long stroke rehabilitation typically lasts:<br><br>1. Acute rehabilitation (inpatient): This phase typically begins within a few days after the stroke and may last for several weeks, usually 2-4 weeks. During this time, the focus is on regaining basic functions, such as mobility, self-care, and communication skills.<br><br>2. Subacute rehabilitation (inpatient or outpatient): After the acute phase, many stroke survivors transition to subacute rehabilitation, which can last anywhere from a few weeks to several months, depending on the individual's progress and goals. This phase focuses on more intensive therapy to improve functional abilities and prepare the individual for community reintegration.<br><br>3. Outpatient rehabilitation: Once discharged from inpatient care, stroke survivors often continue with outpatient rehabilitation

	services, such as physical therapy, occupational therapy, and speech therapy. This phase can last for several months to years, depending on the individual's needs and goals.<br><br>4. Long-term rehabilitation: For some stroke survivors, rehabilitation may be an ongoing process that continues for years, especially if they experience persistent deficits or complications. Long-term rehabilitation may involve periodic therapy sessions, home exercises, and lifestyle modifications.<br><br>It's important to note that stroke recovery is a highly individualized process, and the duration of rehabilitation can vary greatly. Some individuals may recover most of their function within a few months, while others may require ongoing rehabilitation for years. Regular assessments and adjustments to the rehabilitation plan are typically made based on the individual's progress and changing needs.

Gemini responses:

-Prevention

One Shot	Responses
What are the main causes of stroke?	Strokes are caused by disruptions of blood flow to the brain. There are two main types of stroke:<br><br>● Ischemic stroke (8 out of 10 strokes): This is caused by a blood clot blocking a blood vessel that supplies blood to the brain.

Blood clots typically form in areas where arteries have been narrowed or blocked over time by fatty deposits (plaques). This process is known as atherosclerosis.

- Hemorrhagic stroke (less common): This is caused by bleeding in the brain. This bleeding can be caused by high blood pressure, a weakened blood vessel, or an aneurysm (a ballooning or bulging of a blood vessel wall).

Several medical conditions can increase your risk of stroke. Some of these conditions can be controlled with medication and lifestyle changes. Here are some of the main causes of stroke:

- High blood pressure
- High cholesterol
- Diabetes
- Cigarette smoking
- Obesity
- Physical inactivity
- Certain heart conditions, such as atrial fibrillation

	• Sleep apnea
Will I have stroke if my parents had one?	Having a parent with a history of stroke does increase your risk of stroke, but it doesn't mean you will definitely have one. Studies suggest it can increase your risk by 36-44% Healthline [invalid URL removed].<br><br>There are two main reasons for this increased risk:<br><br>• **Genetics:** You may inherit genes from your parents that increase your risk of stroke.<br>• **Shared lifestyle factors:** Families often share similar lifestyles, such as diet, exercise habits, and smoking habits. These lifestyle factors can also contribute to stroke risk.<br><br>Here are some steps you can take to lower your risk of stroke:<br><br>• **Maintain a healthy weight:** Obesity is a major risk factor for stroke.<br>• **Eat a healthy diet:** A diet rich in fruits, vegetables, and whole grains can help lower your blood pressure and cholesterol levels.<br>• **Exercise regularly:** Aim for at least 30 minutes of moderate-intensity exercise most days of the week.

	• **Don't smoke:** Smoking damages your blood vessels and increases your risk of stroke.<br>• **Control your blood pressure:** High blood pressure is a major risk factor for stroke.<br>• **Manage other medical conditions:** If you have diabetes, high cholesterol, or sleep apnea, it's important to control these conditions to reduce your risk of stroke.<br>• **See your doctor regularly:** Your doctor can help you identify and manage your risk factors for stroke.<br><br>By following these steps, you can significantly reduce your risk of stroke, even if you have a family history of the condition.
What can I do to reduce the risk of stroke?	There are several things you can do to reduce your risk of stroke, which focus on overall healthy lifestyle habits:<br><br>• **Maintain a healthy diet:** This means eating plenty of fruits, vegetables, and whole grains while limiting unhealthy fats, processed foods, and added sugar. Look into dietary plans like the DASH (Dietary Approaches to Stop Hypertension) diet which is designed to help control blood pressure https://www.mayoclinic.org/healthy-lifestyle/nutrition-and-healthy-eating/in-depth/dash-diet/art-20048456.<br>• **Exercise regularly:** Aim for at least 150 minutes of moderate-intensity aerobic activity each week. This could be brisk walking,

	swimming, biking, or anything that gets your heart rate up.<br>● **Manage your weight:** Being overweight or obese increases your risk of stroke. Shedding some pounds can significantly improve your health.<br>● **Control your blood pressure:** High blood pressure is a major risk factor for stroke. If you have high blood pressure, work with your doctor to develop a plan to bring it under control. This may involve medication and lifestyle changes.<br>● **Reduce cholesterol:** High cholesterol can lead to fatty deposits in your arteries, which can increase your risk of stroke. If you have high cholesterol, your doctor can recommend ways to lower it, such as medication, diet, and exercise.<br>● **Limit alcohol consumption:** Excessive alcohol consumption can increase your blood pressure and risk of stroke.<br>● **Don't smoke:** Smoking is a major risk factor for stroke. If you smoke, quitting is one of the best things you can do for your health.<br>● **Manage other health conditions:** If you have diabetes, sleep apnea, or other health conditions, it's important to manage them effectively, as they can also increase your risk of stroke.<br>● **See your doctor regularly:** Regular checkups can help identify risk factors for stroke early on, so you can take steps to reduce your risk.

	Remember, these are general recommendations, and it's always best to talk to your doctor about your individual risk factors and the best ways to reduce your risk of stroke.
What types of exercise can help decrease the risk of stroke?	There are many types of exercise that can help decrease the risk of stroke. Here are a few of the most beneficial:<br><br>• **Aerobic exercise** strengthens your heart and lungs and improves blood circulation. Examples of aerobic exercise include brisk walking, running, swimming, biking, and dancing.<br>• **Strength training** helps to build strong muscles, which can help to lower blood pressure and improve cholesterol levels. Examples of strength training exercises include weightlifting, bodyweight exercises, and resistance band exercises.<br>• **Balance exercises** can help to improve your coordination and stability, which can help to reduce your risk of falls. Examples of balance exercises include standing on one leg, tai chi, and yoga.<br><br>Even small amounts of exercise can be beneficial. The American Heart Association recommends that adults get at least 150 minutes of moderate-intensity aerobic activity or 75 minutes of vigorous-intensity aerobic activity each week.

	If you're new to exercise, it's important to start slowly and gradually increase the intensity and duration of your workouts. You should also talk to your doctor before starting any new exercise program.
What should I eat to help prevent a stroke?	Here are some food categories that can be part of a stroke-preventive diet:<br><br>● **Fruits and Vegetables:** Aim for a variety! These are packed with vitamins, minerals, and fiber, all helpful for heart and brain health. They can help lower blood pressure and inflammation. Particularly beneficial fruits include berries, citrus fruits, and melons, while leafy greens and cruciferous vegetables like broccoli are good choices for vegetables [1].<br>● **Fish:** Especially fatty fish like salmon, mackerel, and tuna are great sources of omega-3 fatty acids. Omega-3s can help lower blood pressure and reduce inflammation, reducing stroke risk [2].<br>● **Whole Grains:** Whole grains are higher in fiber than refined grains, and fiber helps manage cholesterol levels. Examples include brown rice, quinoa, and oats [3].<br>● **Low-Fat Dairy:** Low-fat dairy products like yogurt and milk can provide calcium, potassium, and vitamin D, all important for heart health [2].

	• **Foods rich in Potassium and Magnesium:** Look for bananas, potatoes, spinach, and legumes. Potassium helps regulate blood pressure, while magnesium may also play a role in stroke prevention [2, 3].<br><br>Remember, these are just some guidelines. It's best to consult a doctor or registered dietitian for a personalized dietary plan to fit your needs.

-Diagnosis

One shot	Responses
I've been having headaches lately. Could it be a stroke?	Headaches can be a symptom of a stroke, but it's also a very common occurrence that usually has a less serious cause. Here's how to tell the difference:<br><br>• **Stroke-related headaches are usually sudden and severe.** They may be the worst headache you've ever had.<br>• Regular headaches may be throbbing, aching, or a pressure sensation. They may worsen with activity or head movement.<br>• **Stroke headaches are often accompanied by other symptoms of stroke.** These include:<br>○ Weakness or numbness on one side of the face or body

	○ Slurred speech or difficulty speaking<br>○ Vision problems in one or both eyes<br>○ Dizziness or trouble walking<br><br>**If you are experiencing any of these symptoms in addition to your headache, call emergency services immediately.**<br><br>Here are some other things to consider:<br><br>● **Have you had a headache like this before?** If so, it's probably not a stroke.<br>● **Do you have any other risk factors for stroke?** These include high blood pressure, high cholesterol, diabetes, and smoking.<br><br>**If you are concerned that your headache may be a stroke, err on the side of caution and seek medical attention immediately.** Early treatment of stroke can minimize brain damage and improve your chances of a full recovery.
I've felt numbness in my hand and stiffness in my	Numbness in a hand and stiffness in the neck could be a stroke, but it's also possible it's caused by something else. Here's why:<br><br>● **Stroke symptoms:** Numbness on one side of the body, along with other stroke symptoms like sudden weakness, trouble

neck. Could it be a stroke?	speaking, confusion, or facial drooping, can be a sign of stroke. These symptoms typically come on very suddenly. If you are experiencing these other symptoms, you should call 911 immediately.<br>• **Other causes:** There are many other reasons why you might have numbness in your hand and stiffness in your neck, such as sleeping on your arm wrong, a pinched nerve, or carpal tunnel syndrome.<br><br>**Here's what to do:**<br><br>• If you are experiencing other stroke symptoms along with the numbness and stiffness, call 911 immediately.<br>• If you are only experiencing numbness and stiffness, it's still a good idea to see a doctor to get a diagnosis and rule out any serious causes.<br><br>I can't give medical advice, so I recommend you refer to the following resources to learn more about stroke symptoms and when to seek medical attention:<br><br>• American Stroke Association<br>• National Institutes of Health

I've been feeling dizzy lately. Could it be a stroke?	Dizziness is a common feeling that can be caused by many things, including dehydration, inner ear problems, and certain medications. However, it can also be a symptom of a stroke.<br><br>Here are some of the other symptoms of stroke:<br><br>● Sudden numbness or weakness in the face, arm, or leg, especially on one side of the body<br>● Sudden confusion, trouble speaking or understanding speech<br>● Sudden trouble seeing in one or both eyes<br>● Sudden trouble walking, dizziness, loss of balance, or coordination<br>● Severe headache with no known cause<br><br>If you are experiencing any of these symptoms, it is important to call 911 immediately. A stroke is a medical emergency, and prompt treatment is critical to minimize brain damage.<br><br>**Dizziness itself is not usually a cause for concern, but if you are also experiencing any of the other symptoms of stroke, it is important to seek medical attention immediately.**

	Here are some tips to help you determine if your dizziness is a cause for concern:<br><br>● Have you had dizziness before? If so, was it similar to what you are experiencing now?<br>● Are you experiencing any other symptoms, such as nausea, vomiting, or headache?<br>● Do you have any risk factors for stroke, such as high blood pressure, high cholesterol, or diabetes?<br><br>If you are not sure whether or not your dizziness is a cause for concern, it is always best to err on the side of caution and see a doctor.
My blood pressure has been higher lately. Could it be a stroke?	High blood pressure can be a risk factor for stroke, but it doesn't necessarily mean you're having one right now. Stroke symptoms come on very suddenly and are severe.<br><br>Here are some of the warning signs of stroke:<br><br>● Sudden numbness or weakness in face, arm, or leg, especially on one side of the body<br>● Sudden confusion, trouble speaking or understanding speech<br>● Sudden trouble seeing in one or both eyes

	- Sudden trouble walking, dizziness, loss of balance or coordination - Sudden severe headache **If you are experiencing any of these symptoms, call 911 immediately.**<br><br>However, it is important to see a doctor as soon as possible if your blood pressure has been high lately. Early diagnosis and treatment of high blood pressure can help reduce your risk of stroke and other serious health problems.<br><br>Here are some resources that you may find helpful: - American Heart Association [invalid URL removed] on high blood pressure and stroke - Mayo Clinic [invalid URL removed] on stroke symptoms
What symptoms could be a sign of stroke?	A stroke is a medical emergency that happens when blood flow to part of the brain is interrupted. Brain cells can't get the oxygen and nutrients they need, and they start to die. The longer a stroke goes untreated, the more brain cells die.<br><br>Here are the main symptoms of stroke to remember using the acronym FAST:

- **F - Face Drooping** Does one side of the face droop or is it numb? Ask the person to smile. If one side of the face droops, it could be a sign of a stroke.
- **A - Arm Weakness** Is one arm weak or numb? Ask the person to raise both arms. If one arm drifts downward, it could be a sign of a stroke.
- **S - Speech Difficulty** Is speech slurred or difficult to understand? Ask the person to repeat a simple sentence. If their speech is slurred or difficult to understand, it could be a sign of a stroke.
- **T - Time to call emergency services** If you see any of these signs, call emergency services immediately. Every minute counts. Getting treatment quickly can help prevent lasting damage.

Other possible symptoms of stroke include:

- Sudden numbness or weakness of the face, arm or leg on one side of the body
- Sudden confusion, trouble speaking or understanding speech
- Sudden trouble seeing in one or both eyes
- Sudden trouble walking, dizziness, loss of balance or coordination

	• Sudden severe headache with no known cause<br><br>**Remember:** Not everyone will experience all of these symptoms. If you think you or someone you know may be having a stroke, **call emergency services immediately**.

-Treatment

One shot	Responses
Are there any medications or treatments that can help clear blood plaque blockages?	There is currently no medication that can directly dissolve or remove existing plaque buildup in arteries. However, there are medications and procedures that can help prevent plaque buildup from worsening and reduce the risk of complications like heart attack and stroke.<br><br>• **Cholesterol-lowering medications:** These medications, such as statins, can help lower LDL ("bad") cholesterol levels and raise HDL ("good") cholesterol levels. This can help slow down the buildup of plaque in the arteries.<br>• **Blood thinners:** These medications, such as aspirin, can help prevent blood clots from forming on plaque buildup in the arteries.

	• **Angiotensin-converting enzyme (ACE) inhibitors or angiotensin II receptor blockers (ARBs):** These medications can help lower blood pressure and relax blood vessels, which can improve blood flow and reduce the risk of plaque buildup.<br><br>In some cases, medical procedures may be necessary to address significant plaque buildup:<br><br>• **Angioplasty and stenting:** This procedure involves inserting a thin tube with a balloon into the blocked artery. The balloon is inflated to open the artery, and a stent (a small metal tube) may be inserted to help keep the artery open.<br>• **Bypass surgery:** This surgery involves creating a new passage for blood to flow around the blocked artery. A blood vessel from another part of the body is grafted (attached) to the blocked artery above and below the blockage.<br><br>**It is important to note that these medications and procedures should only be done under the supervision of a doctor.** If you are concerned about plaque buildup in your arteries, please see a doctor to discuss your risk factors and treatment options.<br><br>Here are some lifestyle changes that can also help prevent plaque buildup:<br><br>• Eat a healthy diet low in saturated and trans fats, cholesterol, and sodium.<br>• Get regular exercise.

	● Maintain a healthy weight.<br>● Don't smoke.<br>● Manage stress.
What treatments are available for acute stroke?	There are two main types of stroke: ischemic stroke and hemorrhagic stroke. The most common type of stroke is ischemic stroke, which is caused by a blood clot blocking blood flow to the brain. Hemorrhagic stroke is caused by a weakened blood vessel in the brain that bursts.<br><br>The main treatment for ischemic stroke is a clot-busting medication called tissue plasminogen activator (tPA). This medication is most effective if given within 4.5 hours of the stroke. Other treatments for ischemic stroke may include blood thinners, surgery to remove the clot, and rehabilitation.<br><br>There is no clot-busting medication for hemorrhagic stroke. Treatment for this type of stroke focuses on controlling the bleeding and reducing pressure in the brain. This may include medications to lower blood pressure, surgery to repair the weakened blood vessel, or embolization to block off the bleeding vessel.

	Here are some of the treatments available for acute stroke:<br><br>● **Clot-busting medication (tPA):** This medication is only effective for ischemic stroke and must be given within 4.5 hours of the stroke.<br>● **Blood thinners:** These medications can help to prevent new blood clots from forming.<br>● **Surgery:** Surgery may be necessary to remove a blood clot or to repair a weakened blood vessel.<br>● **Rehabilitation:** Rehabilitation can help people who have had a stroke to regain their strength, coordination, and speech.<br><br>If you think someone is having a stroke, it is important to call 911 immediately. The sooner treatment is started, the better the chance of a full recovery.
What are the potential side effects of medication for stroke?	There are many different medications used to treat and prevent stroke, and each one can have its own set of side effects. Some of the most common side effects of stroke medications include:<br><br>● **Bleeding** is a common side effect of many stroke medications, especially blood thinners such as aspirin, warfarin, and clopidogrel. These medications help to prevent blood clots from forming, but they can also increase your risk of bleeding.

	• **Low blood pressure** can be a side effect of medications that are used to lower blood pressure, such as ACE inhibitors, ARBs, and diuretics.<br>• **Dizziness** can be a side effect of many stroke medications, including blood thinners, blood pressure medications, and cholesterol-lowering medications.<br>• **Headache** is another common side effect of stroke medications.<br>• **Nausea and vomiting** can occur with some stroke medications.<br>• **Upset stomach** can be a side effect of some stroke medications.<br>• **Diarrhea** can be a side effect of some stroke medications.<br>• **Muscle cramps** can occur with some stroke medications.<br>• **Skin rash** can be a side effect of some stroke medications.<br><br>It is important to be aware of the potential side effects of any medication you are taking, including stroke medications. If you experience any side effects that are bothersome or that you think may be serious, be sure to talk to your doctor.<br><br>Here are some additional things to keep in mind about the side effects of stroke medications:

	• The severity of side effects can vary from person to person.<br>• Some side effects may go away on their own over time.<br>• There may be ways to reduce the risk of side effects, such as taking your medication with food or at a different time of day.<br>• Your doctor can help you weigh the benefits of taking stroke medication against the risks of side effects.
Is it possible for the brain-damaged area to recover?	Brain damage can be caused by a variety of factors, including stroke, traumatic brain injury (TBI), and tumors. The brain has a remarkable ability to heal itself, and the extent of recovery depends on the severity of the damage and the specific area of the brain that is affected.<br><br>Neurons, the brain's messaging cells, cannot regenerate. However, the brain can recover from damage through a process called neuroplasticity. Neuroplasticity allows the brain to reorganize itself by forming new connections between neurons and strengthening existing ones. This process can help the brain to compensate for the loss of function caused by damage.<br><br>The brain's ability to neuroplasticity is greatest in childhood, but it continues throughout life. This means that it is always possible for the brain to recover from damage, even in adulthood.

	Rehabilitation therapies, such as physical therapy, occupational therapy, and speech therapy, can help to improve function after brain damage. These therapies can help to strengthen existing neural pathways and to create new ones.<br><br>The prognosis for recovery from brain damage varies depending on the severity of the damage and the specific area of the brain that is affected. Some people make a full recovery, while others experience permanent impairments. However, even in cases of severe brain damage, there is always the potential for improvement.
What should I expect during surgery?	Surgery can involve several stages, and what you experience will depend on the specific procedure you're having. Here's a general outline of what to expect:<br><br>**Before Surgery:**<br><br>● **Consultation:** You'll discuss your medical history, medications, and the surgery itself with your doctor. This is a chance to ask questions and voice any concerns you have.<br>● **Pre-surgical tests:** Depending on the surgery, you may need blood tests, imaging tests (X-rays, CT scans), or an EKG (electrocardiogram) to assess your health and ensure surgery is safe.

- **Anesthesia consultation:** You'll meet with an anesthesiologist to discuss the type of anesthesia you'll receive (general, regional, local) to manage pain during surgery.

Day of Surgery:

- **Check-in:** You'll be admitted to the hospital and change into a gown. A nurse will take your vital signs and start an IV line for fluids and medications.
- **Operating room:** Once prepped, you'll be wheeled into the operating room. The environment will be sterile, and the surgical team will be wearing protective gear.
- **Anesthesia:** Depending on the type chosen, you'll be given medication to put you to sleep (general), numb a specific area (regional), or stay awake but relaxed (local).
- **The surgery:** The surgeon and their team will perform the operation as planned. You won't feel pain during surgery while under anesthesia.

After Surgery:

- **Recovery room:** After surgery, you'll be monitored in a recovery room until awake and stable.

	• **Hospital stay:** Depending on the surgery, you may go home the same day or stay in the hospital for a few days.<br>• **Recovery at home:** You'll likely have pain and discomfort after surgery. Your doctor will prescribe medication and provide instructions for pain management, wound care, and recovery.<br><br>It's important to remember that this is a general overview. Always talk to your doctor about what to specifically expect during your surgery. They can address your individual situation and answer any questions you have.

-Recovery

One shot	Responses
Can rehabilitation and hyperbaric oxygen therapy help restore brain function after a stroke?	Yes, both rehabilitation and hyperbaric oxygen therapy (HBOT) have shown promise in helping restore brain function after a stroke.<br><br>**Rehabilitation** is the cornerstone of stroke recovery. It involves a variety of therapies designed to help you relearn lost skills and improve your function. Rehabilitation can include:<br><br>• Physical therapy: This helps you improve your movement, strength, and coordination.<br>• Occupational therapy: This helps you learn or relearn how to do everyday activities, such as dressing, bathing, and eating.

- Speech therapy: This helps you improve your communication skills, such as speaking, understanding language, and swallowing.

Rehabilitation is most effective when started early and continued for a long period of time.

Hyperbaric oxygen therapy (HBOT) is a treatment that involves breathing 100% oxygen in a pressurized chamber. HBOT is thought to work by increasing the amount of oxygen that reaches the damaged tissues in the brain. This may help to reduce inflammation, promote healing, and improve blood flow.

The research on the effectiveness of HBOT for stroke recovery is mixed. Some studies have shown that HBOT can improve motor function, speech, and cognitive function. However, other studies have not found any benefit.

Here's a table summarizing the findings on rehabilitation and HBOT for stroke recovery:

Intervention	Effect
Rehabilitation	Promising results for

	restoring brain function<br><br>Hyperbaric Oxygen Therapy — Limited evidence, more research needed<br><br>**Overall, rehabilitation is the best-established treatment for stroke recovery. HBOT may be a promising option, but more research is needed to determine its effectiveness.**<br><br>If you are considering HBOT for stroke recovery, it is important to talk to your doctor. HBOT is not right for everyone, and it can be expensive.
Can rehabilitation and intravenous laser irradiation of blood help restore brain function after a stroke?	Yes, both rehabilitation and intravenous laser irradiation of blood (ILIB) have shown promise in helping to restore brain function after a stroke.<br><br>**Rehabilitation** is the cornerstone of stroke recovery. It involves a variety of therapies designed to help you relearn lost skills and

	improve your function. These therapies may include:<br><br>• Physical therapy: This helps you improve your movement, coordination, and strength.<br>• Occupational therapy: This helps you relearn how to do everyday activities, such as dressing, bathing, and eating.<br>• Speech therapy: This helps you improve your communication skills, such as speaking, understanding, and swallowing.<br><br>**Intravenous laser irradiation of blood (ILIB)** is a newer treatment that is still being studied. It involves shining a low-level laser into a vein to irradiate the blood. This is thought to improve blood flow, reduce inflammation, and promote healing in the brain.<br><br>**Studies on ILIB**<br><br>While research is ongoing, some studies have shown positive results for ILIB:<br><br>• A study published in the journal **Photobiomodulation, Photomedicine, and Laser Surgery** found that ILIB improved functional independence in subacute stroke patients [1].<br>• Another study reported a case where ILIB helped improve cognitive function in a stroke survivor [2].

	**Important to Consider**<br><br>● ILIB is considered an **investigational treatment**. More research is needed to confirm its effectiveness and safety.<br>● ILIB is not a replacement for standard stroke rehabilitation. It should be used in conjunction with other therapies.<br>● ILIB is not widely available and may not be covered by insurance.<br><br>**If you are considering ILIB, it is important to talk to your doctor about the risks and benefits.**<br><br>Here are some resources where you can learn more about stroke rehabilitation and ILIB:<br><br>● **National Institute of Neurological Disorders and Stroke:** https://www.ninds.nih.gov/<br>● **American Stroke Association:** https://www.stroke.org/en/
Can rehabilitation and transcranial magnetic stimulation aid in restoring brain function after a	Yes, both rehabilitation and transcranial magnetic stimulation (TMS) can aid in restoring brain function after a stroke.<br><br>**Rehabilitation** is the cornerstone of stroke recovery. It helps people relearn skills they may have lost due to the stroke, such as movement, speech, and swallowing. Rehabilitation can also help improve strength, coordination, and balance. There are many

stroke?	different types of rehabilitation, and the best type of rehabilitation for a person will depend on the individual's needs.<br><br>**Transcranial magnetic stimulation (TMS)** is a newer treatment that is being used to help people recover from stroke. TMS is a non-invasive procedure that uses magnetic fields to stimulate the brain. TMS is thought to work by helping to rewire the brain after a stroke. Studies have shown that TMS can improve motor function, language function, and cognitive function in people who have had a stroke.<br><br>**Here's a summary of the benefits of rehabilitation and TMS for stroke recovery:**<br><br>● **Rehabilitation:**<br>○ Improves motor function, speech, swallowing, strength, coordination, and balance<br>○ Helps people relearn skills they may have lost due to the stroke<br>● **TMS:**<br>○ Improves motor function, language function, and cognitive function<br>○ May help to rewire the brain after a stroke<br><br>**It is important to note that both rehabilitation and TMS are most effective when used together.** Rehabilitation helps people to

	practice and use their recovered skills, while TMS may help to improve the brain's ability to learn and recover.<br><br>If you have had a stroke, talk to your doctor about whether rehabilitation and/or TMS may be right for you.
What assistance does stroke rehabilitation offer?	Stroke rehabilitation is a crucial part of recovery after a stroke. It helps people regain independence and improve their quality of life. Here are some of the ways stroke rehabilitation can assist people:<br><br>• **Physical therapy:** This helps improve movement, balance, and coordination. Physical therapists will work with you on exercises to help you relearn how to move your body.<br>• **Occupational therapy:** This helps you practice daily tasks such as eating, bathing, and dressing. Occupational therapists will help you find ways to do these tasks safely and independently.<br>• **Speech-language therapy:** This helps you improve your ability to speak, understand language, and swallow. Speech-language pathologists will work with you on exercises to improve your communication skills.<br>• **Cognitive therapy:** This helps you improve your thinking skills, such as memory, problem-solving, and attention. Cognitive therapists will teach you strategies to help you cope with cognitive problems.

	• **Emotional support:** Stroke can be a very emotional experience. Rehabilitation can provide emotional support to help you cope with the changes in your life. This may include individual counseling or support groups.<br><br>Stroke rehabilitation is a personalized process. The type of therapy you receive will depend on your specific needs and goals. Rehabilitation can take place in a variety of settings, such as hospitals, outpatient clinics, and home care.<br><br>Remember, it is important to start rehabilitation as soon as possible after a stroke. The sooner you start, the better your chances of recovery.
How long does stroke rehabilitation typically last?	Stroke rehabilitation is a long-term process that can last for months or even years. The duration of rehabilitation depends on a number of factors, including the severity of the stroke, the person's age and overall health, and the amount of brain damage that occurred.<br><br>• **The first 3 months** after a stroke are considered the most important for recovery. This is when the brain is most receptive to rehabilitation and people are likely to see the most improvement.<br>• **After 6 months**, recovery typically slows down, but some people may continue to make progress for up to a year or more.

	Even though recovery eventually plateaus, stroke rehabilitation can still be beneficial in helping people maintain their independence and improve their quality of life. There is also some evidence that people can continue to make gains in function even years after a stroke.<br><br>Here are some tips for getting the most out of stroke rehabilitation:<br><br>● Start rehabilitation as soon as possible after a stroke.<br>● Participate in all therapy sessions as prescribed by your doctor.<br>● Practice rehabilitation exercises at home on a regular basis.<br>● Set realistic goals for your recovery.<br>● Be patient and don't get discouraged if you don't see results immediately.

GPT responses:

-Prevention

COT	Responses

My dad recently suffered a stroke. I’m not really sure what caused this. Can you give me some ideas?	I'm sorry to hear about your dad. A stroke can have several causes, and understanding them can be helpful in preventing future occurrences. Here are some common causes and risk factors for strokes:<br><br>1. **High Blood Pressure (Hypertension)**: The most significant risk factor for stroke. High blood pressure can damage blood vessels and make them more prone to blockages or bursting.<br>2. **Atherosclerosis**: This condition involves the buildup of fatty deposits (plaque) in the arteries, which can lead to blockages that reduce blood flow to the brain.<br>3. **Heart Disease**: Conditions like atrial fibrillation (an irregular heartbeat) can cause blood clots that travel to the brain.<br>4. **Diabetes**: This condition can damage blood vessels and nerves, increasing the risk of a stroke.<br>5. **Smoking**: Smoking can damage blood vessels, raise blood pressure, and increase the risk of blood clots.<br>6. **High Cholesterol**: High levels of cholesterol can lead to the buildup of plaques in the arteries.<br>7. **Obesity and Physical Inactivity**: Being overweight and not engaging in regular physical activity can increase the risk of stroke.<br>8. **Excessive Alcohol Consumption**: Drinking too much alcohol can raise blood pressure and contribute to stroke risk.<br>9. **Age and Gender**: The risk of stroke increases with age, and men are generally at higher risk than women, although women tend to have more severe strokes.<br>10. **Family History**: A family history of stroke or cardiovascular disease can increase the risk.<br>11. **Previous Stroke or Transient Ischemic Attack (TIA)**: Having had a stroke or TIA in the past increases the likelihood of having another stroke.<br>12. **Other Medical Conditions**: Certain conditions like sickle cell disease, blood clotting disorders, and autoimmune diseases can increase stroke risk.<br><br>Understanding these risk factors can help in managing and potentially reducing the risk of future strokes. If your dad's doctors haven't already discussed these factors with you, it might be helpful to have a conversation with them to understand his specific situation better and to get personalized advice.

My parents recently suffered a stroke. Does it mean that I will have one too?	I'm sorry to hear about your parents. Having a family history of stroke can increase your risk, but it doesn't mean you will definitely have one. Several factors influence stroke risk, including genetics, lifestyle, and other health conditions. Here are some key points:<br><br>1. **Genetics**: A family history of stroke can increase your risk, especially if close relatives had strokes at a young age.<br>2. **Lifestyle Factors**: Smoking, excessive alcohol consumption, a poor diet, and lack of exercise can increase stroke risk. Adopting a healthy lifestyle can significantly reduce this risk.<br>3. **Health Conditions**: Conditions like high blood pressure, diabetes, high cholesterol, and atrial fibrillation can increase stroke risk. Managing these conditions with medication and lifestyle changes is crucial.<br>4. **Preventive Measures**: Regular check-ups, maintaining a healthy weight, eating a balanced diet, exercising regularly, not smoking, and limiting alcohol can help lower your risk.<br><br>It's essential to discuss your family history and any concerns with your healthcare provider. They can provide personalized advice and recommend strategies to reduce your risk.
My dad recently suffered a stroke. Maybe by changing my lifestyle, I can reduce the risk of stroke. Do you have any ideas?	I'm sorry to hear about your dad. Reducing the risk of stroke involves making several lifestyle changes that can significantly improve your overall health. Here are some key strategies:<br><br>**1. Maintain a Healthy Diet**<br><br>● **Eat More Fruits and Vegetables:** Aim for at least 5 servings a day.<br>● **Reduce Salt Intake:** High salt intake can raise blood pressure, increasing stroke risk.<br>● **Choose Whole Grains:** Whole grain foods such as oatmeal, brown rice, and whole wheat bread can help lower cholesterol.<br>● **Limit Saturated and Trans Fats:** These fats can increase cholesterol levels. Opt for healthy fats like those found in olive oil, nuts, and avocados.

- **Consume Omega-3 Fatty Acids:** Found in fish like salmon and mackerel, these can help reduce stroke risk.

2. Exercise Regularly

- **Aim for at Least 150 Minutes of Moderate Exercise Per Week:** Activities like walking, swimming, or cycling can improve cardiovascular health.
- **Include Strength Training:** Two or more days a week can help maintain muscle mass and improve metabolism.

3. Maintain a Healthy Weight

- **Calculate Your Body Mass Index (BMI):** Aim for a BMI within the normal range (18.5–24.9).
- **Monitor Waist Circumference:** A high waist circumference can indicate higher risk; aim for less than 40 inches (102 cm) for men and 35 inches (88 cm) for women.

4. Control Blood Pressure

- **Regularly Monitor Blood Pressure:** Aim for a target of less than 120/80 mm Hg.
- **Follow a DASH Diet:** The Dietary Approaches to Stop Hypertension (DASH) diet is rich in fruits, vegetables, and low-fat dairy.

5. Manage Cholesterol Levels

- **Get Regular Blood Tests:** Keep an eye on your cholesterol levels.
- **Incorporate Fiber:** Foods high in soluble fiber can help lower cholesterol levels.

6. Limit Alcohol Consumption

- **Drink in Moderation:** Up to one drink per day for women and up to two drinks per day for men.

<table>
<tr><td></td><td>

7. Avoid Smoking

Quit Smoking: Smoking doubles the risk of stroke. Seek support groups or counseling if needed.

8. Manage Diabetes

Control Blood Sugar Levels: Keep them within a target range through diet, exercise, and medication if necessary.

9. Reduce Stress

Practice Relaxation Techniques: Activities such as yoga, meditation, or deep breathing exercises can help reduce stress.

10. Regular Health Check-Ups

Schedule Regular Doctor Visits: Regular check-ups can help detect and manage health issues early on.

11. Stay Hydrated

Drink Plenty of Water: Aim for at least 8 glasses a day, or more if you are physically active.

12. Get Adequate Sleep

Aim for 7-9 Hours of Sleep per Night: Poor sleep can negatively affect cardiovascular health.

By incorporating these lifestyle changes, you can significantly reduce your risk of stroke and improve your overall health. If you have any specific concerns or conditions, it’s best to consult with a healthcare professional for personalized advice.

</td></tr>
<tr><td>My dad recently</td><td>I'm sorry to hear about your dad. Exercise can indeed play a crucial role in preventing strokes and improving overall cardiovascular</td></tr>
</table>

suffered a stroke. Perhaps I can prevent this by doing more exercise. Do you have any ideas?	health. Here are some exercise ideas that can help reduce the risk:<br><br>**Aerobic Exercises**<br><br>1. **Walking**: Aim for at least 30 minutes of brisk walking most days of the week.<br>2. **Jogging/Running**: Start with short distances and gradually increase as your fitness improves.<br>3. **Cycling**: Both outdoor cycling and stationary biking are excellent options.<br>4. **Swimming**: A low-impact, full-body workout that's great for cardiovascular health.<br><br>**Strength Training**<br><br>1. **Weightlifting**: Use free weights or weight machines at the gym.<br>2. **Bodyweight Exercises**: Push-ups, squats, lunges, and planks.<br>3. **Resistance Bands**: Versatile and can be used anywhere for strength training.<br><br>**Flexibility and Balance**<br><br>1. **Yoga**: Improves flexibility, balance, and mental well-being.<br>2. **Tai Chi**: A gentle exercise that also helps with balance and stress reduction.<br><br>**Other Tips**<br><br>1. **Consistency**: Aim for at least 150 minutes of moderate-intensity or 75 minutes of vigorous-intensity aerobic activity each week.<br>2. **Mix It Up**: Vary your workouts to keep things interesting and work different muscle groups.<br>3. **Stay Hydrated**: Drink plenty of water before, during, and after exercise.<br>4. **Warm-Up and Cool Down**: Start with a warm-up and end with a cool down to prevent injury.

	Before starting any new exercise regimen, it's a good idea to consult with a healthcare provider, especially if you have any existing health conditions or concerns.
My dad recently suffered a stroke. Perhaps I can be healthier by changing the way I eat. Can taking fish oil or other health supplements help?	I'm sorry to hear about your dad. Making healthier dietary choices can indeed have a positive impact on your health and potentially reduce your risk of conditions like strokes. Here are some ways fish oil and other health supplements might help, along with general dietary advice:<br><br>**Fish Oil**<br><br>Fish oil is rich in omega-3 fatty acids, which have been shown to:<br><br>● Reduce inflammation<br>● Lower blood pressure<br>● Decrease triglyceride levels<br>● Reduce the risk of heart disease<br><br>**Other Health Supplements**<br><br>● **Vitamin D**: Supports overall health, immune function, and may help reduce the risk of chronic diseases.<br>● **Magnesium**: Important for muscle and nerve function, blood sugar levels, and blood pressure regulation.<br>● **Coenzyme Q10 (CoQ10)**: May help improve heart health and reduce the risk of heart-related conditions.<br><br>**General Dietary Advice**<br><br>● **Increase Fruits and Vegetables**: They are high in vitamins, minerals, and antioxidants.<br>● **Choose Whole Grains**: Opt for whole grains over refined grains to get more fiber and nutrients.

	• **Reduce Saturated and Trans Fats**: Limit intake of fatty meats, full-fat dairy products, and processed foods.<br>• **Eat Lean Protein**: Include sources like fish, poultry, beans, and legumes.<br>• **Limit Salt**: Reducing sodium intake can help lower blood pressure.<br>• **Stay Hydrated**: Drink plenty of water throughout the day.<br><br>**Lifestyle Changes**<br><br>• **Regular Physical Activity**: Aim for at least 150 minutes of moderate-intensity exercise per week.<br>• **Quit Smoking**: Smoking significantly increases the risk of stroke and other cardiovascular diseases.<br>• **Manage Stress**: Practice stress-reducing techniques such as mindfulness, meditation, or yoga.<br><br>It's always a good idea to consult with a healthcare provider before starting any new supplements, especially if you have existing health conditions or are taking other medications. They can provide personalized advice based on your specific health needs.

-Diagnosis

COT	Responses
I've been having headaches quite often these days, especially on cold days. They seem to be getting worse with time. Could it be a sign	I'm not a medical professional, but frequent headaches can be caused by a variety of factors, not necessarily indicating a stroke. Common causes include tension, migraines, sinus issues, and changes in weather, among others. Cold weather can sometimes trigger headaches due to changes in blood pressure or sinus congestion.<br><br>However, it's essential to be aware of stroke symptoms, which can include:<br><br>- Sudden numbness or weakness, particularly on one side of the body<br>- Confusion, trouble speaking, or understanding speech

of stroke?	- Sudden vision problems in one or both eyes<br>- Difficulty walking, dizziness, loss of balance or coordination<br>- Severe headache with no known cause<br><br>If you experience any of these symptoms, seek medical attention immediately.<br><br>Given your recurring headaches and their worsening nature, it would be best to consult a healthcare provider to rule out any serious conditions and get a proper diagnosis. They can perform a thorough evaluation and recommend appropriate treatment or further testing if needed.
I woke up this morning with my hand feeling numb. After a while, I noticed that my neck was sort of stiff too. Could these be the symptoms of a stroke?	While numbness in the hand and a stiff neck can be concerning, they are not necessarily indicative of a stroke. Stroke symptoms typically include sudden onset of the following:<br><br>1. Weakness or numbness, especially on one side of the body<br>2. Confusion, trouble speaking, or understanding speech<br>3. Difficulty seeing in one or both eyes<br>4. Trouble walking, dizziness, or loss of balance and coordination<br>5. Severe headache with no known cause<br><br>Your symptoms could also be related to other conditions, such as sleeping in an awkward position, poor posture, or nerve compression. However, if you are concerned or if you experience any of the more typical stroke symptoms mentioned above, it is important to seek medical attention immediately.<br><br>It's always best to consult a healthcare professional for an accurate diagnosis and appropriate care.

I've been feeling dizzy for several weeks already. It seems to be getting worse with time. Could it be a stroke?	I'm sorry to hear that you're experiencing dizziness. While dizziness can be a symptom of a variety of conditions, including inner ear issues, dehydration, low blood sugar, and anxiety, it can also be a sign of more serious conditions, such as a stroke.<br><br>**Symptoms of a Stroke to Watch For:**<br><br>1. **Sudden numbness or weakness** in the face, arm, or leg, especially on one side of the body.<br>2. **Sudden confusion**, trouble speaking, or understanding speech.<br>3. **Sudden trouble seeing** in one or both eyes.<br>4. **Sudden trouble walking**, dizziness, loss of balance, or coordination.<br>5. **Sudden severe headache** with no known cause.<br><br>If you are experiencing any of these symptoms, it is important to seek medical attention immediately. Stroke is a medical emergency, and prompt treatment is crucial.<br><br>**Other Common Causes of Dizziness:**<br><br>● **Inner ear issues**: Such as vertigo or Meniere's disease.<br>● **Dehydration**: Not drinking enough fluids.<br>● **Low blood sugar**: Especially if you haven't eaten for a while.<br>● **Low blood pressure**: Sudden drops can cause dizziness.<br>● **Medications**: Some medications have dizziness as a side effect.<br>● **Anxiety and stress**: Can also cause dizziness.<br><br>**Steps You Can Take in the Meantime:**<br><br>● **Stay Hydrated**: Ensure you're drinking enough water throughout the day.

	● **Eat Regular Meals**: To maintain your blood sugar levels.<br>● **Avoid Sudden Movements**: Such as standing up too quickly.<br>● **Rest**: Get plenty of sleep and avoid stress.<br><br>**When to See a Doctor:**<br><br>Dizziness alone is not enough to diagnose a stroke, and it's best to consult with a healthcare professional who can evaluate your symptoms in detail, perform necessary tests, and provide a proper diagnosis. Please consider seeing a doctor as soon as possible to discuss your symptoms and get a thorough evaluation.
My blood pressure has been higher than usual these days. So high that my head actually hurts even when I go for a walk. Could it be a stroke?	I'm not a doctor, but I can provide some general information. High blood pressure can be a serious condition, and persistent headaches, especially when associated with elevated blood pressure, can be a warning sign that something needs to be addressed.<br><br>**Potential Causes and Considerations:**<br><br>1. **Hypertension (High Blood Pressure):**<br>○ **Persistent Headaches:** Consistently high blood pressure can lead to headaches and other symptoms. It's important to monitor your blood pressure regularly.<br>○ **Monitoring:** Keep track of your blood pressure readings and discuss them with your healthcare provider.<br>2. **Hypertensive Crisis:**<br>○ **Severe Increase in Blood Pressure:** This is a severe increase in blood pressure that can lead to a stroke.<br>○ **Symptoms:** Severe headache, confusion, vision problems, chest pain, and difficulty breathing.<br>○ **Immediate Action:** This requires immediate medical attention. If you suspect a hypertensive crisis, seek emergency medical care.<br>3. **Stroke:**

	◦ **Sudden Severe Headaches:** A stroke can cause sudden and severe headaches, along with other symptoms.<br>◦ **Additional Symptoms:** Weakness, numbness (especially on one side of the body), trouble speaking, vision problems, dizziness, and loss of balance.<br>◦ **Emergency Response:** A stroke is a medical emergency. Call emergency services immediately if you suspect a stroke.<br><br>**Immediate Steps:**<br><br>• **Seek Medical Attention:** Given your symptoms, it’s very important to seek medical attention as soon as possible. A healthcare professional can provide an accurate diagnosis and appropriate treatment.<br>• **Monitor Symptoms:** If you are experiencing severe symptoms or if your condition worsens, go to the emergency room immediately.<br><br>High blood pressure and associated symptoms should not be taken lightly. Consulting with a healthcare professional is crucial to address and manage your condition effectively.
Since my dad's stroke, I've been alert for any signs.I understand that recognizing symptoms early can be crucial. Could you list the exact symptoms?	Recognizing the symptoms of a stroke early is crucial for getting timely medical help. Here are the main signs and symptoms to watch for, often remembered by the acronym FAST:<br><br>1. **Face Drooping**: One side of the face may droop or feel numb. Ask the person to smile; if the smile is uneven, it could be a sign of a stroke.<br>2. **Arm Weakness**: One arm may be weak or numb. Ask the person to raise both arms; if one arm drifts downward, it may indicate a stroke.<br>3. **Speech Difficulty**: The person may have slurred speech or be unable to speak or understand speech. Ask them to repeat a simple sentence; difficulty doing so could be a warning sign.<br>4. **Time to Call Emergency Services**: If any of these symptoms are present, it's important to call emergency services immediately

	and note the time when the symptoms first appeared.<br><br>**Other Symptoms Can Include:**<br><br>● **Sudden Confusion or Trouble Understanding**: Difficulty thinking clearly or understanding spoken language.<br>● **Sudden Trouble Seeing**: Blurred, blackened, or double vision in one or both eyes.<br>● **Sudden Trouble Walking**: Loss of balance, dizziness, or lack of coordination.<br>● **Sudden Severe Headache**: A sudden, intense headache with no known cause.<br><br>**Immediate Action:**<br><br>If you notice any of these signs, it's essential to seek medical attention immediately. Quick action can greatly improve the chances of recovery.

-Treatment

COT	Responses
I've just been diagnosed with stroke. The doctor says that there is severe blood plaque blockage. Are there any medications or treatments that	I'm really sorry to hear about your diagnosis. The treatment for stroke and blood plaque blockage typically involves a combination of medications, lifestyle changes, and sometimes surgical procedures. Here are some common treatments:<br><br>**Medications**<br><br>1. **Antiplatelet Drugs**: Medications like aspirin or clopidogrel help prevent new clots from forming.<br>2. **Anticoagulants**: Medications such as warfarin, heparin, or newer agents like dabigatran can help prevent blood clots.<br>3. **Statins**: These are used to lower cholesterol levels and help stabilize plaques. Examples include atorvastatin and simvastatin.<br>4. **Blood Pressure Medications**: Controlling blood pressure is crucial to reduce the risk of further strokes. Common medications

can help clear it?	include ACE inhibitors, beta-blockers, and calcium channel blockers.<br>5. **Thrombolytic Therapy**: For some types of stroke, especially ischemic stroke, tissue plasminogen activator (tPA) can dissolve clots if administered within a certain time window.<br><br>**Lifestyle Changes**<br><br>1. **Diet**: A heart-healthy diet that is low in saturated fats, trans fats, cholesterol, and sodium.<br>2. **Exercise**: Regular physical activity can help improve overall cardiovascular health.<br>3. **Smoking Cessation**: Quitting smoking is one of the most important steps to improve heart health.<br>4. **Weight Management**: Maintaining a healthy weight reduces the strain on your heart and blood vessels.<br><br>**Surgical and Other Procedures**<br><br>1. **Angioplasty and Stent Placement**: A procedure to open narrowed or blocked blood vessels that supply blood to the heart.<br>2. **Carotid Endarterectomy**: Surgery to remove plaque buildup from the carotid arteries in the neck, which supply blood to the brain.<br>3. **Bypass Surgery**: Creating a new pathway for blood to flow around blocked arteries.<br><br>**Rehabilitation and Support**<br><br>1. **Physical Therapy**: Helps regain motor skills and improve muscle strength and coordination through exercises tailored to the patient’s needs.<br>2. **Occupational Therapy**: Assists in relearning daily activities and skills such as dressing, cooking, and writing.<br>3. **Speech and Language Therapy**: Aids in recovering communication skills and addressing difficulties with speaking, understanding, reading, and writing.<br>4. **Cognitive Rehabilitation**: Focuses on improving memory, attention, problem-solving, and other cognitive functions affected

	by the stroke.<br><br>It's important to work closely with your healthcare team to determine the best treatment plan for your specific condition. They can provide tailored advice based on your overall health, the severity of the blockage, and other individual factors.
I've just been diagnosed with acute stroke. Perhaps with the right treatment I will be able to get better. Do you have any suggestions?	I'm sorry to hear about your diagnosis. Acute stroke requires immediate and comprehensive medical attention. Here are some steps and suggestions that might help:<br><br>**Immediate Medical Attention**<br><br>1. **Follow Medical Advice**: It's crucial to follow the treatment plan prescribed by your healthcare provider. This may include medications to dissolve blood clots, lower blood pressure, and prevent future clots.<br><br>**Rehabilitation**<br><br>2. **Physical Therapy**: Helps regain motor skills and improve muscle strength and coordination through exercises tailored to your needs.<br>3. **Occupational Therapy**: Assists in relearning daily activities and skills such as dressing, cooking, and writing.<br>4. **Speech and Language Therapy**: Aids in recovering communication skills and addressing difficulties with speaking, understanding, reading, and writing.<br>5. **Cognitive Rehabilitation**: Focuses on improving memory, attention, problem-solving, and other cognitive functions affected by the stroke.<br><br>**Lifestyle Changes**<br><br>6. **Healthy Diet**: Adopt a heart-healthy diet rich in fruits, vegetables, whole grains, and lean proteins. Reducing salt and fat

	intake can help manage blood pressure and cholesterol levels.<br>7. **Exercise**: Under your doctor's guidance, engage in regular physical activity. Exercise can improve cardiovascular health and help in recovery.<br>8. **Manage Risk Factors**: Control risk factors such as hypertension, diabetes, and high cholesterol. Regular check-ups and medication adherence are key.<br>9. **Avoid Smoking and Limit Alcohol**: If you smoke, quitting is crucial. Limiting alcohol intake is also important for overall health.<br>10. **Stay Hydrated and Rest**: Drink plenty of fluids and ensure you get enough rest. Your body needs to recover, and proper hydration and sleep are essential.<br><br>**Support and Monitoring**<br><br>11. **Support System**: Lean on family, friends, and support groups. Emotional and mental support is vital during recovery.<br>12. **Monitor Symptoms**: Keep track of any changes in your condition and report them to your healthcare provider. This includes any new symptoms or worsening of existing ones.<br><br>**Education and Awareness**<br><br>13. **Education and Awareness**: Educate yourself and those around you about stroke signs and symptoms to ensure prompt action in case of future episodes.<br><br>**Long-Term Care**<br><br>14. **Regular Follow-Ups**: Schedule regular follow-up appointments with your healthcare provider to monitor your progress and make any necessary adjustments to your treatment plan.<br>15. **Medication Management**: Take your medications as prescribed and discuss any side effects or concerns with your doctor.<br><br>**Mental Health**

	16. **Mental Health Support**: Consider counseling or therapy to help cope with the emotional impact of having a stroke. Anxiety and depression are common, and professional support can be very beneficial.<br><br>**Patience and Persistence**<br><br>Recovery can vary from person to person, so patience and persistence are key. Stay positive and proactive in your recovery journey. Celebrate small victories along the way and keep a hopeful outlook on your progress.<br><br>By incorporating these steps and staying in close communication with your healthcare team, you can optimize your recovery and improve your overall well-being.
I've just been prescribed some medication for stroke. However, there seem to be some potential side effects. How long do I need to keep taking them?	The duration for which you need to take stroke medication depends on the specific type of medication, your overall health, the severity of your stroke, and your doctor's recommendations. Common types of medication prescribed for stroke include:<br><br>1. **Antiplatelet agents (e.g., aspirin, clopidogrel):** These are often taken long-term to prevent blood clots. Your doctor will determine the duration based on your condition.<br>2. **Anticoagulants (e.g., warfarin, dabigatran):** These are also typically taken long-term to prevent further strokes, especially if you have conditions like atrial fibrillation.<br>3. **Blood pressure medications (e.g., ACE inhibitors, beta-blockers):** These may be prescribed to control blood pressure and are usually taken long-term.<br>4. **Cholesterol-lowering drugs (e.g., statins):** These are often taken for life to manage cholesterol levels and reduce stroke risk.<br><br>It's important to follow your doctor's instructions regarding medication duration. Never stop taking your medication without consulting your doctor, even if you experience side effects. If you do experience side effects, inform your healthcare provider, as they may adjust your dosage or prescribe an alternative.

	Would you like more detailed information on any specific medication you're taking?
I've just been diagnosed with a severe stroke in the brain. Is it possible for the damaged area to recover?	I'm really sorry to hear about your diagnosis. Recovery from a stroke can vary greatly from person to person and depends on several factors, including the severity of the stroke, the specific area of the brain affected, and how quickly treatment was received.<br><br>1. **Neuroplasticity**: The brain has a remarkable ability to reorganize itself by forming new neural connections. This ability, called neuroplasticity, can help compensate for damaged areas. Rehabilitation therapies often focus on stimulating neuroplasticity.<br>2. **Early Intervention**: The sooner treatment begins, the better the chances for recovery. This can include medication, physical therapy, occupational therapy, and speech therapy, depending on the stroke's effects.<br>3. **Rehabilitation**: Intensive rehabilitation can significantly improve outcomes. This might involve a combination of therapies aimed at improving mobility, strength, coordination, speech, and daily living skills.<br>4. **Support Systems**: Having a strong support system, including family, friends, and healthcare professionals, can greatly influence recovery outcomes.<br>5. **Lifestyle Changes**: Adopting a healthy lifestyle, including a balanced diet, regular exercise, quitting smoking, and managing underlying health conditions (like hypertension or diabetes), can help support recovery and prevent future strokes.<br>6. **Patience and Persistence**: Recovery from a stroke can be a long and challenging process. Progress may be slow, and it's important to stay patient and persistent with rehabilitation efforts.<br><br>Each stroke and recovery journey is unique. Working closely with your healthcare team to develop a personalized rehabilitation plan is essential. They can provide specific guidance and support tailored to your needs.
I am about to undergo surgery for stroke. What should I expect in	I'm sorry to hear that you need surgery for a stroke. Understanding what to expect can help ease some anxiety. Here's a general overview of what you might experience:

the operating room?	**Before Surgery**<br><br>1. **Preparation**:<br>○ You will be asked to change into a hospital gown.<br>○ Medical staff will check your vital signs (blood pressure, heart rate, oxygen levels).<br>○ An intravenous (IV) line will be inserted to administer fluids and medications.<br>○ You may receive medications to help you relax.<br>2. **Anesthesia**:<br>○ Depending on the type of surgery, you may receive general anesthesia (which puts you to sleep) or local anesthesia (which numbs the area but keeps you awake).<br><br>**During Surgery**<br><br>1. **Positioning**:<br>○ You will be positioned on the operating table in a way that provides the surgeons the best access to the area they need to work on.<br>2. **Monitoring**:<br>○ Throughout the procedure, your vital signs will be continuously monitored.<br>○ There will be a team of medical professionals, including surgeons, anesthesiologists, and nurses.<br>3. **The Procedure**:<br>○ The specifics of the procedure will depend on the type of surgery. Common types of stroke-related surgeries include:<br>■ **Carotid Endarterectomy**: Removing plaque from the carotid artery to prevent future strokes.<br>■ **Mechanical Thrombectomy**: Removing a blood clot from a large artery in the brain.<br>■ **Aneurysm Clipping or Coiling**: For brain aneurysms, either clipping the aneurysm or filling it with coils to prevent rupture.<br>■ **Decompressive Craniectomy**: Removing part of the skull to relieve pressure on the brain.

	4. **Duration**:<br>○ The length of the surgery can vary greatly depending on the procedure. Your surgical team can give you a more specific time frame.<br><br>**After Surgery**<br><br>1. **Recovery Room**:<br>○ You will be taken to a recovery room where you will be closely monitored as you wake up from anesthesia.<br>○ Nurses will check your vital signs and neurological status frequently.<br>2. **Pain Management**:<br>○ You will be given pain medication as needed.<br>○ It's important to communicate with the medical staff about your pain levels.<br>3. **Hospital Stay**:<br>○ The length of your hospital stay will depend on the type of surgery and how well you are recovering.<br>○ You may be moved to an intensive care unit (ICU) for close monitoring.<br>4. **Rehabilitation**:<br>○ Depending on your condition, you may begin physical, occupational, or speech therapy while still in the hospital.<br>○ Long-term rehabilitation might continue after you leave the hospital to help you regain function and independence.<br><br>**Emotional Support**<br><br>● It's normal to feel anxious or scared. Don’t hesitate to talk to your medical team about any concerns you have.<br>● Support from family and friends can also be very comforting.<br><br>Please let me know if you have any specific questions or need more detailed information.

-Recovery

COT	Responses
I am trying to recover from stroke. I wonder if rehabilitation and hyperbaric oxygen therapy can help restore brain function.	I'm sorry to hear about your stroke. Rehabilitation and hyperbaric oxygen therapy (HBOT) are two approaches that have been explored to help with stroke recovery.<br><br>**Rehabilitation**<br><br>1. **Physical Therapy (PT)**: Helps restore movement and function. Includes exercises to improve strength, coordination, and balance.<br>2. **Occupational Therapy (OT)**: Focuses on improving daily living skills and independence. Includes tasks like dressing, eating, and bathing.<br>3. **Speech and Language Therapy**: Assists with communication and swallowing difficulties.<br>4. **Psychological Therapy**: Addresses emotional and cognitive challenges, providing support and strategies to cope with changes.<br>5. **Neurological Rehabilitation**: Comprehensive programs often include a combination of the above therapies tailored to individual needs.<br><br>**Hyperbaric Oxygen Therapy (HBOT)**<br><br>HBOT involves breathing pure oxygen in a pressurized chamber. The idea is that increasing oxygen levels in the blood can help repair damaged tissues and promote healing. Here's what research says:<br><br>1. **Potential Benefits**:<br>○ **Improved Brain Function**: Some studies suggest HBOT can enhance neuroplasticity, which is the brain's ability to form new connections and repair itself.

- **Reduced Inflammation**: HBOT may help reduce inflammation and swelling in brain tissue.
- **Enhanced Recovery**: There is evidence that HBOT can improve cognitive and motor functions in some stroke patients.

2. **Research Evidence**:
   - **Mixed Results**: While some studies show promising results, others find no significant benefit. More research is needed to determine the effectiveness and optimal protocols.
   - **Safety**: HBOT is generally considered safe, but it's essential to undergo this therapy under medical supervision to manage potential risks like barotrauma (injury caused by pressure changes) or oxygen toxicity.

Combining Therapies

Combining traditional rehabilitation with HBOT might offer the best outcomes for some patients. Here are steps to consider:

1. **Consult Healthcare Providers**: Discuss with your neurologist or stroke specialist about the potential benefits and risks of HBOT in your specific case.
2. **Personalized Rehabilitation Plan**: Work with your rehabilitation team to create a tailored plan that addresses your unique needs.
3. **Monitor Progress**: Regularly assess your progress and adjust therapies as needed.

Support and Resources

- **Stroke Support Groups**: Connecting with others who have experienced similar challenges can provide emotional support and practical advice.
- **Educational Materials**: Access resources from organizations like the American Stroke Association for more information on stroke recovery.

Would you like more detailed information on any specific therapy or additional resources for stroke recovery?

I am trying different methods to recover from a stroke. I wonder if rehabilitation and intravenous laser irradiation of blood help restore brain function.	I'm sorry to hear about the situation. Rehabilitation and intravenous laser irradiation of blood (ILIB) are two approaches that have been explored for stroke recovery. Here's some information about each:<br><br>**Rehabilitation**<br><br>**1. Physical Therapy:**<br>● Focuses on improving mobility, strength, and coordination. Techniques include exercises, walking practice, and balance training.<br><br>**2. Occupational Therapy:**<br>● Aims to improve daily living skills such as dressing, eating, and using the bathroom. Therapists help patients relearn activities of daily living (ADLs).<br><br>**3. Speech Therapy:**<br>● Assists with language, communication, and swallowing difficulties. Speech therapists work on regaining speech and language skills.<br><br>**4. Cognitive Rehabilitation:**<br>● Targets cognitive functions like memory, attention, and problem-solving. Therapists use exercises and tasks to improve cognitive skills.

	**5. Psychological Support:**<br><br>● Addressing emotional and mental health is crucial. Counseling and support groups can help patients and their families cope with the emotional aftermath of a stroke.<br><br>**Intravenous Laser Irradiation of Blood (ILIB)**<br><br>ILIB is a relatively newer and less common approach. It involves exposing the blood to low-level laser light, which is believed to have various therapeutic effects:<br><br>**1. Anti-inflammatory Effects:**<br><br>● ILIB is thought to reduce inflammation, which can be beneficial after a stroke.<br><br>**2. Enhanced Blood Flow:**<br><br>● It may improve microcirculation and oxygen delivery to tissues, potentially aiding in recovery.<br><br>**3. Immune Modulation:**<br><br>● The therapy might help modulate the immune response, reducing damage and promoting healing.<br><br>**4. Cellular Energy Production:**<br><br>● ILIB is suggested to enhance the energy production of cells (mitochondrial function), which can support the repair processes.<br><br>**Effectiveness and Research**<br><br>**Rehabilitation:**

	• There is strong evidence supporting the effectiveness of various rehabilitation therapies in improving functional outcomes after a stroke. Consistent and intensive rehabilitation can significantly enhance recovery.<br><br>**ILIB:**<br><br>• Research on ILIB for stroke recovery is still emerging. Some studies suggest potential benefits, but the evidence is not as robust as for conventional rehabilitation therapies. More clinical trials and studies are needed to establish its efficacy and safety.<br><br>**Recommendations**<br><br>• **Consult Healthcare Professionals:** Always discuss with a neurologist or a rehabilitation specialist to tailor a recovery plan specific to the patient's needs.<br>• **Combined Approach:** A combination of traditional rehabilitation and exploring newer therapies like ILIB, under medical guidance, might offer comprehensive recovery support.<br><br>Each stroke patient's recovery journey is unique, and a multifaceted approach often yields the best results.
I am trying to recover from stroke. I wonder if rehabilitation and transcranial magnetic stimulation aid in restoring brain	Rehabilitation and transcranial magnetic stimulation (TMS) can indeed play significant roles in stroke recovery. Here's how each can aid in restoring brain function:<br><br>**Rehabilitation**<br><br>1. **Physical Therapy**: Focuses on improving motor skills, strength, and coordination. Exercises and activities are designed to enhance mobility and physical function.<br>2. **Occupational Therapy**: Helps in regaining the ability to perform daily activities independently, such as dressing, eating, and

function.	bathing. It also includes exercises to improve fine motor skills and cognitive functions.<br>3. **Speech and Language Therapy**: Assists in recovering communication skills, including speaking, understanding language, reading, and writing. This is crucial if the stroke has affected areas of the brain responsible for language.<br>4. **Cognitive Rehabilitation**: Involves exercises and activities that help improve memory, attention, problem-solving skills, and other cognitive functions that may be impaired after a stroke.<br>5. **Psychological Support**: Addresses emotional and psychological challenges post-stroke, such as depression and anxiety. Counseling and support groups can be beneficial.<br><br>**Transcranial Magnetic Stimulation (TMS)**<br><br>TMS is a non-invasive procedure that uses magnetic fields to stimulate nerve cells in the brain. It has shown promise in aiding stroke recovery by:<br><br>1. **Enhancing Neural Plasticity**: TMS can promote brain plasticity, helping the brain reorganize and form new connections to compensate for damaged areas.<br>2. **Improving Motor Function**: Studies have shown that TMS can improve motor skills and hand function in stroke survivors by stimulating the motor cortex.<br>3. **Aiding Cognitive Function**: TMS can also enhance cognitive functions, such as memory and attention, which may be affected by a stroke.<br>4. **Reducing Depression**: TMS has been used to treat depression, which is common in stroke survivors. By alleviating depression, it can improve overall well-being and motivation for rehabilitation.<br><br>**Comprehensive Rehabilitation Plan**<br><br>It's essential to work with a healthcare team to develop a comprehensive rehabilitation plan tailored to your specific needs. They can guide you on the best combination of therapies, including whether TMS is suitable for your situation. Regular follow-ups and

	adjustments to your rehabilitation plan are crucial to ensure optimal recovery.<br><br>By combining traditional rehabilitation methods with innovative treatments like TMS, stroke survivors can maximize their recovery potential and improve their quality of life.
I would like to know how useful rehabilitation is for stroke patients. What assistance does doing so provide?	Rehabilitation is crucial for stroke patients as it helps them regain as much independence as possible and improve their quality of life. Here are the key benefits and types of assistance provided by stroke rehabilitation:<br><br>**Benefits of Stroke Rehabilitation**<br><br>1. **Restoring Function**: Rehabilitation helps restore lost motor and cognitive functions. It can significantly improve the ability to perform daily activities.<br>2. **Preventing Complications**: It helps prevent complications such as muscle atrophy, joint stiffness, and pressure sores.<br>3. **Improving Mobility**: Patients can regain mobility through exercises and physical therapy, which helps them move more easily and safely.<br>4. **Enhancing Speech and Communication**: Speech therapy can improve communication skills and address issues such as aphasia (difficulty with speaking or understanding language).<br>5. **Boosting Mental Health**: Rehabilitation can help reduce feelings of depression and anxiety, providing psychological support.<br>6. **Increasing Independence**: With improved physical and cognitive functions, patients can become more independent in their daily lives.<br><br>**Types of Assistance in Stroke Rehabilitation**<br><br>1. **Physical Therapy**: Focuses on improving strength, coordination, and mobility through exercises and activities.<br>2. **Occupational Therapy**: Helps patients relearn daily activities, such as dressing, bathing, and cooking, and adapt to physical limitations.

	3. **Speech and Language Therapy**: Addresses communication issues, helping patients improve speech, language, and swallowing functions.<br>4. **Cognitive Therapy**: Aims to improve memory, attention, problem-solving skills, and other cognitive functions.<br>5. **Psychological Support**: Provides counseling and support to deal with the emotional impact of a stroke.<br>6. **Adaptive Equipment**: Introduction and training in the use of devices like wheelchairs, walkers, or communication aids.<br>7. **Nutritional Counseling**: Ensures patients receive a proper diet to support recovery and overall health.<br><br>**Personalized Rehabilitation Programs**<br><br>Rehabilitation programs are tailored to each patient's specific needs, considering the severity of the stroke, the areas affected, and the patient's overall health and personal goals. This individualized approach maximizes the effectiveness of the rehabilitation process.<br><br>**Continuous and Long-term Rehabilitation**<br><br>Rehabilitation is often a long-term process, requiring continuous effort and support. Regular follow-ups and adjustments to the rehabilitation plan ensure that patients continue to make progress and address any new challenges that arise.<br><br>By participating in rehabilitation, stroke patients can make significant strides in their recovery, regain independence, and improve their overall well-being.
I was recently discharged from hospital after being treated for stroke. The doctor	The duration of stroke rehabilitation can vary widely depending on the severity of the stroke and the individual's specific needs. Generally, stroke rehabilitation can last for several weeks to several months. Here are some general timelines:<br><br>1. **Initial Phase:** The first few weeks after a stroke are critical for starting rehabilitation. This phase typically takes place in a hospital or an inpatient rehabilitation facility and may last 1 to 3 weeks.

says that I need to go back for rehabilitation on a weekly basis. How long does it usually last?	2. **Outpatient Rehabilitation:** After the initial phase, many patients continue rehabilitation on an outpatient basis. This can last from a few weeks to several months, with sessions typically occurring several times a week.<br>3. **Long-term Rehabilitation:** Some individuals may need long-term rehabilitation, which can last for several months to years. This often involves less frequent sessions and may include home-based exercises and therapies.<br><br>The exact duration and frequency of your rehabilitation will be determined by your healthcare team based on your progress and specific needs. It's essential to follow your doctor's recommendations and attend all scheduled rehabilitation sessions to achieve the best possible recovery outcomes.

Claude responses:

-Prevention

COT	Responses
My dad recently suffered a stroke. I'm not really sure what caused this. Can you give me some ideas?	I'm very sorry to hear that your dad suffered a stroke. A stroke can be a devastating event, and I understand this must be an incredibly difficult and worrying time for you and your family. There are several potential causes and risk factors for strokes:<br><br>- High blood pressure (hypertension) is one of the leading causes of strokes. Over time, uncontrolled high blood pressure can damage blood vessel walls.<br><br>- High cholesterol levels can lead to buildup of fatty deposits or plaques in the arteries, which can block blood flow to the brain.<br><br>- Smoking significantly increases stroke risk by damaging blood vessels and promoting clot formation.<br><br>- Diabetes raises stroke risk as it can contribute to narrowed arteries and high blood pressure.

	- Being overweight or obese strains the circulatory system.<br><br>- Atrial fibrillation, an irregular heartbeat, allows blood to pool and clot, which can travel to the brain.<br><br>- Heavy alcohol use, lack of exercise, and stress can also increase risk.<br><br>Sometimes strokes occur due to genetic factors or other underlying conditions as well. The most important next steps are ensuring your dad receives proper medical treatment, diagnostic tests to determine the cause, and management of risk factors. Don't hesitate to have open conversations with his doctors. I wish you and your family all the best during this difficult period.
My parents recently suffered a stroke. Does it mean that I will have one too?	I'm sorry to hear about your parents suffering strokes. That must be a difficult and concerning situation for your family. While stroke risk does have some hereditary factors, having a parent who has had a stroke does not necessarily mean you will definitely have one too. There are several key points about stroke risk that may help provide some reassurance:<br><br>- Stroke risk is multi-factorial - it depends on a combination of genetic and lifestyle/environmental factors like diet, exercise, smoking, managing conditions like high blood pressure, diabetes, etc.<br><br>- Making healthy lifestyle choices like not smoking, eating a nutritious diet, exercising regularly, and managing chronic conditions can significantly reduce your stroke risk even with a family history.<br><br>- If you are concerned about your stroke risk based on your parents' history, it's a good idea to discuss it with your doctor. They may recommend earlier screening for risk factors and provide personalized prevention guidance.<br><br>- Only a portion of the risk is genetic/hereditary - having an affected parent increases your risk, but it does not inevitably mean you

	will have a stroke as well.<br><br>The key is being proactive about understanding your overall risk profile, getting appropriate screenings, and taking steps to control any modifiable risk factors you can through a healthy lifestyle. With proper management, having a parental history does not have to mean your risk is uncontrollable. I'd suggest discussing your specific concerns with your doctor.
My dad recently suffered a stroke. Maybe by changing my lifestyle, I can reduce the risk of stroke. Do you have any ideas?	I'm sorry to hear about your dad's stroke. Dealing with a loved one's stroke can be very difficult. Making lifestyle changes is indeed one of the best ways to reduce your own risk of stroke. Here are some tips that may help:<br><br>1. Manage blood pressure: High blood pressure is a major risk factor for stroke. Adopt a diet low in sodium and rich in fruits, vegetables, and whole grains. Exercise regularly, maintain a healthy weight, and don't smoke. If prescribed, take blood pressure medications as directed.<br><br>2. Control cholesterol: High cholesterol contributes to plaque buildup in arteries. Eat a diet low in saturated and trans fats. Get regular exercise and take cholesterol-lowering medications if needed.<br><br>3. Keep diabetes in check: Diabetes increases stroke risk. If diabetic, follow your doctor's advice on using medications, diet, and exercise to control blood sugar levels.<br><br>4. Exercise regularly: Aerobic exercise like brisk walking can lower blood pressure, increase good cholesterol, and improve circulation.<br><br>5. Eat a healthy diet: Focus on fruits, vegetables, whole grains, lean proteins, and healthy fats like olive oil and nuts. Limit sodium, sweets, and red/processed meats.

	6. Maintain a healthy weight: Being overweight or obese increases stroke risk factors like high blood pressure.<br><br>7. Don't smoke: Smoking doubles the risk of stroke by damaging blood vessel walls.<br><br>8. Limit alcohol: Heavy or binge drinking can lead to stroke.<br><br>Making positive lifestyle changes can significantly reduce your stroke risk over time. Be patient and consistent. Consult your doctor as well for personalized guidance.
My dad recently suffered a stroke. Perhaps I can prevent this by doing more exercise. Do you have any ideas?	I'm sorry to hear about your dad's stroke. Strokes can certainly be devastating events. You're wise to consider lifestyle changes that may help reduce your own risk.<br><br>Exercise is one of the best things you can do to prevent stroke and other cardiovascular issues. Some exercise tips that may help:<br><br>- Aim for at least 150 minutes per week of moderate aerobic activity like brisk walking, or 75 minutes per week of vigorous aerobic activity like running or swimming laps. Aerobic exercise improves cardiovascular health.<br><br>- Do strength training 2-3 times per week to build muscle and keep bones strong. This can be weightlifting, resistance bands, yoga, etc.<br><br>- If you have a sedentary job, take frequent breaks to stand up, stretch, and move around a bit every hour.<br><br>- Consider taking up an enjoyable active hobby like hiking, cycling, dancing, or playing a sport.<br><br>- Build more walking into your daily routine - park further away, take the stairs instead of elevator, etc.

	In addition to exercise, other key stroke prevention strategies include not smoking, eating a healthy diet rich in fruits/veggies and low in saturated fat, managing conditions like high blood pressure and diabetes, and maintaining a healthy weight. But exercise is one of the most important controllable factors. Start slow and build up a routine you can stick with long-term.
My dad recently suffered a stroke. Perhaps I can be healthier by changing the way I eat. Can taking fish oil or other health supplements help?	I'm sorry to hear about your dad's stroke. Strokes can be very serious, and seeing a loved one go through that must be difficult. It's understandable that you want to take steps to improve your own health in light of this experience.<br><br>When it comes to fish oil and other supplements for general health, the evidence is somewhat mixed. Here's a quick overview:<br><br>Fish Oil:<br>- Fish oil is rich in omega-3 fatty acids which have anti-inflammatory properties.<br>- Some studies suggest fish oil may help reduce risk of heart disease and stroke when combined with a healthy lifestyle.<br>- However, the evidence that fish oil supplements alone provide significant benefits is not conclusive.<br><br>Other Supplements:<br>- A daily multivitamin is reasonable for filling any nutrient gaps in your diet.<br>- Vitamin D may be helpful if you don't get enough from sunlight exposure.<br>- Antioxidants like vitamin C, E, etc. have not been proven to provide significant benefits from supplements.<br><br>The most important things are to focus on an overall healthy dietary pattern and lifestyle:<br><br>- Eat plenty of fruits, vegetables, whole grains, lean protein and healthy fats from plant and fish sources.<br>- Limit processed foods, saturated fats, sodium and added sugars.<br>- Get regular physical activity and maintain a healthy weight.

	- Don't smoke and limit alcohol intake.<br><br>Supplements can be useful in some cases, but making long-term dietary and lifestyle changes is typically advisable for disease prevention. I'd recommend discussing any supplements with your doctor as well. Let me know if you need any other suggestions!

-Diagnosis

COT	Responses
I've been having headaches quite often these days, especially on cold days. They seem to be getting worse with time. Could it be a sign of stroke?	Frequent or worsening headaches can potentially be a concerning symptom, but they do not necessarily indicate an impending stroke. Here are a few points about headaches and their relation to stroke risk:<br><br>- Headaches alone are very rarely a sign of an imminent stroke. Strokes typically cause other neurological symptoms like weakness on one side of the body, speech difficulties, vision problems, dizziness, or loss of coordination.<br><br>- However, a sudden, severe "thunderclap" headache can be a warning sign of a hemorrhagic stroke caused by bleeding in the brain. These are differentiated from typical headaches or migraines by their maximal intensity at onset.<br><br>- Chronic headaches that worsen over time or change in pattern can sometimes be caused by underlying conditions like brain tumors or structural issues that may increase stroke risk if left untreated.<br><br>- Migraines with aura may slightly increase the long-term risk of stroke in some individuals, especially women on birth control pills or with other vascular risk factors.<br><br>The fact that your headaches seem to worsen in cold weather suggests they may have an environmental trigger like changes in

	atmospheric pressure common with cold fronts. This pattern is more typical of migraines or cluster headaches rather than a stroke warning sign.<br><br>However, any new, persistent or progressively worsening headache warrants medical evaluation to identify the underlying cause and ensure no serious condition is present. Discussing your symptoms with your doctor is advisable for proper diagnosis and management. Sudden neurological deficits require emergency medical care to rule out stroke.
I woke up this morning with my hand feeling numb. After a while, I noticed that my neck was sort of stiff too. Could these be the symptoms of a stroke?	The symptoms you describe - numbness/tingling in the hand and stiff/painful neck - could potentially be signs of a stroke, but they are quite non-specific and can have other causes as well. Here are a few key points about stroke symptoms:<br><br>- Classic stroke symptoms include sudden weakness/numbness on one side of the body, slurred speech, drooping on one side of the face, sudden vision problems, dizziness, and a severe headache.<br><br>- Neck pain or stiffness alone is not a typical stroke symptom, though it can sometimes occur if the stroke affects circulation to the neck muscles.<br><br>- Numbness/tingling in just one hand is less common for a stroke, which more typically affects an entire arm or side of the body.<br><br>While it's certainly possible these could be early stroke symptoms, there are many other potential causes like a pinched nerve, sleeping in an awkward position, or muscle strain/spasm. However, if the symptoms persist or worsen, it's a good idea to get evaluated by a doctor, especially if any more classic stroke signs develop.<br><br>The most concerning thing would be if the numbness/weakness spreads further, your speech becomes garbled, or you develop severe headache, dizziness or vision changes. In that case, seeking immediate medical care is advisable as quick treatment is crucial for strokes. But your current symptoms alone don't necessarily indicate an emergency situation.

I’ve been feeling dizzy for several weeks already. It seems to be getting worse with time. Could it be a stroke?	There could be several potential causes for persistent dizziness that lasts for weeks. While a stroke is one possibility, there are some other more common causes as well. Here are a few considerations:<br><br>- Vertigo - This is a spinning sensation and is often caused by an inner ear disorder like benign paroxysmal positional vertigo (BPPV), Meniere's disease, or vestibular neuritis. Vertigo can last for days or weeks.<br><br>- Dehydration - Being dehydrated can lead to dizziness and light-headedness. Make sure you are drinking enough fluids.<br><br>- Low blood pressure - Orthostatic hypotension (low blood pressure upon standing) can make someone feel dizzy.<br><br>- Medication side effects - Certain medications like blood pressure drugs, antidepressants, etc. can have dizziness as a side effect.<br><br>- Neurological conditions - While less common, problems with the brain like a stroke, tumor, or multiple sclerosis lesions could potentially cause prolonged dizziness.<br><br>The fact that your dizziness has persisted for weeks and seems to be worsening is concerning. A stroke typically causes a sudden onset of symptoms. I would recommend seeing a doctor, especially if you have any other stroke symptoms like slurred speech, arm/leg weakness, vision changes or headache. They can evaluate if further testing like an MRI is needed to check for a stroke or other neurological cause. But there are also other treatable causes they can investigate.
My blood pressure has been higher than usual	A sudden, persistent headache accompanied by high blood pressure could potentially be a sign of a stroke or other serious medical condition. However, it's difficult to self-diagnose based just on those symptoms. Here are a few points to consider:

these days. So high that my head actually hurts even when I go for a walk. Could it be a stroke?	- Stroke symptoms usually come on suddenly and may include weakness/numbness on one side of the body, slurred speech, vision problems, dizziness, and severe headache.<br><br>- High blood pressure alone usually does not cause headaches unless it is extremely high (over 180/120 mmHg).<br><br>- Risk factors like age, diabetes, high cholesterol, smoking increase the chances of stroke occurring with high blood pressure.<br><br>Rather than trying to diagnose yourself, it would be prudent to seek medical evaluation as soon as possible, especially if you are experiencing additional neurological symptoms beyond just the headache and high BP. Call emergency services or go to the ER if the headache is severe, sudden onset or accompanied by concerning symptoms like vision changes, weakness, etc.<br><br>Even if it is not an emergency, make an appointment with your doctor to have your persistently high BP evaluated and treated properly. Untreated hypertension can lead to stroke, heart disease and other complications over time. Don't ignore worrisome symptoms - get them checked out right away.
Since my dad's stroke, I've been alert for any signs.I understand that recognizing symptoms early can be crucial. Could you list the exact symptoms?	Certainly, it's important to be aware of the signs and symptoms of a stroke, which can help ensure prompt medical attention and improve outcomes. Here are the main symptoms to watch out for:<br><br>1. Sudden numbness or weakness in the face, arm, or leg, especially on one side of the body.<br>2. Sudden confusion, trouble speaking, or understanding speech.<br>3. Sudden trouble seeing in one or both eyes.<br>4. Sudden difficulty walking, dizziness, loss of balance, or lack of coordination.<br>5. Sudden severe headache with no known cause.<br><br>The acronym "FAST" is often used to help remember the key signs:

	F - Face drooping<br>A - Arm weakness<br>S - Speech difficulty<br>T - Time to call emergency services<br><br>It's crucial to act quickly if you notice any of these signs, as prompt treatment can be crucial in minimizing brain damage and improving recovery. Even if the symptoms seem to fluctuate or go away, it's still important to seek immediate medical attention.<br><br>Additionally, it's wise to be aware of risk factors for stroke, such as high blood pressure, diabetes, smoking, and heart disease, and work with your healthcare provider to manage these conditions effectively.

-Treatment

COT	Responses
I've just been diagnosed with stroke. The doctor says that there is severe blood plaque blockage. Are there any medications or treatments that can help clear it?	I'm sorry to hear about your stroke diagnosis and the plaque buildup causing a blockage. There are several treatment options available that may help clear the blockage, depending on the location and severity:<br><br>1. Medications:<br>- Antiplatelet drugs (e.g., aspirin, clopidogrel) can help prevent further clot formation.<br>- Anticoagulants (e.g., warfarin, apixaban) can help thin the blood and reduce clot risk.<br>- Statins are cholesterol-lowering drugs that can help stabilize and reduce plaque buildup over time.<br><br>2. Procedures:<br>- Carotid endarterectomy: A surgical procedure to remove plaque buildup from the carotid arteries in the neck, which supply

	blood to the brain.<br>- Carotid artery stenting: A minimally invasive procedure where a small mesh tube (stent) is inserted to open a narrowed carotid artery.<br>- Angioplasty and stenting: A catheter-based procedure to open blocked arteries and insert a stent to help keep the artery open.<br><br>3. Lifestyle changes:<br>- Dietary modifications: Adopting a heart-healthy diet low in saturated fats, trans fats, and cholesterol can help prevent further plaque buildup.<br>- Regular exercise: Engaging in regular physical activity can improve blood flow and help manage risk factors like high blood pressure and obesity.<br>- Smoking cessation: Quitting smoking is crucial as it significantly increases the risk of plaque buildup and stroke.<br><br>It's important to discuss these options with your doctor, who can evaluate your specific situation and recommend the most appropriate course of action. Early intervention and treatment can help prevent further complications and potentially reduce the risk of future stroke events.
I’ve just been diagnosed with acute stroke. Perhaps with the right treatment I will be able to get better. Do you have any suggestions?	I'm so sorry to hear about your stroke diagnosis. That must be very difficult and scary news to receive. Acute stroke is a serious medical emergency that requires prompt treatment to try to minimize long-term disability. Here are some suggestions based on expert medical guidance:<br><br>- Call 911 immediately if you haven't already received emergency medical care. Getting evaluated and treated at a hospital as quickly as possible is crucial with acute stroke.<br><br>- Time is brain when it comes to stroke. The sooner treatments like clot-busting drugs or surgical thrombectomy can be provided, the better the potential outcome.

	- At the hospital, doctors will likely order tests like CT scans or MRI to determine the type of stroke (ischemic or hemorrhagic) and location.<br><br>- For ischemic strokes caused by a blood clot, IV tPA medication may be given to try to dissolve the clot if administered within a few hours of symptom onset.<br><br>- Thrombectomy is another treatment possibility for large clots, where a catheter is used to mechanically remove the clot.<br><br>- Controlling risk factors like high blood pressure, diabetes, high cholesterol through medications and lifestyle changes is important.<br><br>- Rehabilitation involving physical, occupational and speech therapy is usually necessary after a stroke to regain functions.<br><br>The most important things are getting emergency care immediately and following your stroke team's guidance. With prompt care, many stroke patients can make significant recoveries. Let me know if you need any other stroke-related information.
I've just been prescribed some medication for stroke. However, there seem to be some potential side effects. How long do I need to keep taking them?	I don't actually have enough specific information about your medical condition, the prescribed medication, or potential side effects to provide advice on how long you should continue taking the medication. Decisions around medication regimens need to be made in close consultation with your prescribing doctor or pharmacist. They have details on your health history, the intended therapeutic effects, risks and benefits of the particular drug.<br><br>In general, medications prescribed after a stroke are often needed long-term to help prevent another stroke from occurring. However, the ideal duration can vary depending on the situation. Your doctor will weigh the potential side effects against the protective benefits when determining an appropriate course length.

	My recommendation would be to have an open discussion with your doctor about your concerns over the potential side effects. They can best evaluate if the side effects warrant adjusting the dosage or exploring alternative treatments for your specific case. Don't stop taking the prescribed medication without first consulting them, as that could increase your stroke risk. Follow their instructions carefully until any changes are recommended by your physician.
I’ve just been diagnosed with a severe stroke in the brain. Is it possible for the damaged area to recover?	Yes, it is possible for damaged areas of the brain to recover function to some degree after a stroke. The brain has an amazing ability called neuroplasticity that allows undamaged parts to potentially take over functions previously handled by the damaged regions.<br><br>Some key points about brain recovery after a stroke:<br><br>- The earlier rehabilitation starts, the better the potential for recovery. Early physical, occupational, and speech therapy can help retrain the brain.<br><br>- With time and therapy, nearby undamaged brain cells can gradually re-wire themselves and take over responsibilities of the stroke-impacted area.<br><br>- Recovery is best in the few months right after a stroke as the brain is most plastic during this period. However, meaningful gains are still possible years later.<br><br>- Younger brains tend to have better neuroplastic capabilities compared to older brains after a stroke.<br><br>- The location and size of the stroke plays a role - smaller strokes allow more potential for recovery than very large strokes.<br><br>- Everyone's recovery is different based on the severity of the stroke, rehabilitation intensity, age, motivation, and other factors.

	The key is getting the appropriate rehabilitative therapies started as soon as medically allowed after the stroke. With time, repetitive practice, and the brain's plasticity, many stroke survivors can regain capabilities that were initially lost. Maintaining hope and diligence with therapies is important.
I am about to undergo surgery for stroke. What should I expect in the operating room?	When undergoing surgery for a stroke, here's what you can typically expect in the operating room:<br><br>Pre-Op:<br>- You'll change into a hospital gown and have an IV line inserted to receive fluids and medications.<br>- The surgical area will be clipped or shaved to prepare the site.<br>- You may receive anti-nausea, antibiotic, and other medications through your IV before surgery.<br><br>During Surgery:<br>- You'll be taken to the operating room and positioned on the operating table.<br>- Anesthesia will be administered, putting you into a temporary deep sleep.<br>- Monitors will track your vital signs like blood pressure, oxygen levels, and heart rate/rhythm.<br>- The neurosurgeon will make an incision, usually in your skull, to access the area of stroke damage.<br>- Depending on the type of stroke, procedures like clot removal, repairing blood vessel malformations, or relieving pressure on the brain may be performed.<br>- The surgery can take several hours depending on the complexity.<br><br>Post-Op:<br>- You'll be moved to a recovery room and monitored closely as you wake up from anesthesia.<br>- You may have a urinary catheter, drainage tubes, and dressings on the surgical site.<br>- Pain medications will be provided to keep you comfortable.<br>- Neurological exams will check for any deficits or changes from before surgery.

	The surgical team will keep you informed before, during, and after about what to expect. Feel free to ask them any other specific questions or concerns you may have.

-Recovery

COT	Responses
I am trying to recover from stroke. I wonder if rehabilitation and hyperbaric oxygen therapy can help restore brain function.	Yes, both rehabilitation and hyperbaric oxygen therapy can potentially help restore brain function after a stroke.<br><br>Rehabilitation:<br>- Physical, occupational, and speech therapy are key components of stroke rehabilitation.<br>- Physical therapy helps restore movement, balance, and walking ability.<br>- Occupational therapy focuses on relearning daily living skills like dressing, bathing, and cooking.<br>- Speech therapy aims to improve speech, language, and cognitive skills affected by the stroke.<br>- Starting rehab soon after a stroke is important for maximizing recovery.<br><br>Hyperbaric Oxygen Therapy (HBOT):<br>- HBOT involves breathing 100% oxygen inside a pressurized chamber.<br>- It is thought to increase oxygen levels in tissues, which may promote healing and help revive damaged brain cells.<br>- Some studies suggest HBOT can improve neurological function when started within a certain timeframe after stroke.<br>- However, the evidence on HBOT's efficacy is still quite limited and more research is needed.<br><br>The combination of traditional rehabilitation and HBOT may provide synergistic benefits for some stroke survivors. However, it's important to discuss the potential risks and benefits with your doctor, as HBOT is not universally recommended. Adhering to an

	intensive, multi-disciplinary rehabilitation program is currently considered one of the most crucial aspects of post-stroke recovery.
I am trying different methods to recover from a stroke. I wonder if rehabilitation and intravenous laser irradiation of blood help restore brain function.	Stroke rehabilitation and intravenous laser irradiation of blood are two different approaches that may potentially help restore brain function after a stroke. Here's an overview of each method:<br><br>1. Stroke Rehabilitation:<br>Stroke rehabilitation is a well-established and widely used approach that aims to help individuals regain functions lost due to brain damage caused by a stroke. It typically involves a combination of therapies, including:<br><br>a. Physical Therapy: Exercises and activities to improve mobility, strength, balance, and coordination.<br>b. Occupational Therapy: Training to improve daily living skills, such as dressing, eating, and grooming.<br>c. Speech Therapy: Exercises to improve communication skills, including speech, language, and swallowing.<br>d. Cognitive Therapy: Activities and exercises to improve memory, problem-solving, and other cognitive functions.<br><br>Rehabilitation is often tailored to the individual's specific needs and deficits. It can help the brain reorganize and rewire neural connections, a process known as neuroplasticity, which can lead to functional recovery over time.<br><br>2. Intravenous Laser Irradiation of Blood:<br>Intravenous laser irradiation of blood, also known as intravenous laser blood irradiation (ILBI) or photobiomodulation, is a relatively new and experimental approach to stroke treatment. It involves exposing a patient's blood to low-level laser light while it circulates through an intravenous line.<br><br>The proposed mechanism behind ILBI is that the laser light interacts with blood components, such as red blood cells and platelets, triggering various biochemical reactions. These reactions may potentially:

	a. Increase oxygen delivery to tissues, including the brain.<br>b. Reduce inflammation and oxidative stress.<br>c. Stimulate the production of growth factors and cytokines that may promote neuronal repair and regeneration.<br><br>ILBI is still considered an experimental therapy, and more research is needed to establish its efficacy and safety in stroke recovery. Some preliminary studies have reported potential benefits, such as improved neurological function and reduced disability, but larger and more rigorous clinical trials are necessary to confirm these findings.<br><br>It's important to note that both stroke rehabilitation and ILBI should be conducted under the guidance and supervision of qualified healthcare professionals, such as neurologists, physical therapists, and rehabilitation specialists. They can evaluate your specific condition and recommend the most appropriate treatment plan.
I am trying to recover from stroke. I wonder if rehabilitation and transcranial magnetic stimulation aid in restoring brain function.	Yes, rehabilitation and transcranial magnetic stimulation (TMS) can help aid in restoring brain function after a stroke.<br><br>Rehabilitation:<br>- Physical, occupational, and speech therapy are common forms of stroke rehabilitation.<br>- Physical therapy helps improve mobility, strength, balance, and coordination.<br>- Occupational therapy focuses on relearning daily living activities like dressing, bathing, and cooking.<br>- Speech therapy works on communication skills like speaking, understanding speech, reading, and writing.<br>- Rehabilitation takes advantage of the brain's neuroplasticity to rewire neural pathways and regain lost functions.<br><br>Transcranial Magnetic Stimulation (TMS):<br>- TMS is a non-invasive technique that uses magnetic fields to stimulate nerve cells in the brain.<br>- It can increase or decrease the activity of specific brain regions.<br>- For stroke recovery, repetitive TMS (rTMS) is often used on the affected brain areas.

	- rTMS aims to restore normal levels of brain activity and facilitate reorganization of neural networks.<br>- Studies have shown rTMS combined with rehabilitation can improve motor function, language skills, and cognitive abilities post-stroke.<br><br>The combination of rehabilitation exercises and therapies along with techniques like TMS can help the brain reshape its neural connections and recover functions impaired by the stroke. An individualized treatment plan is ideal for maximizing recovery potential.
I would like to know how useful rehabilitation is for stroke patients. What assistance does doing so provide?	Rehabilitation is crucial for stroke patients as it can help them regain functions that were impaired or lost due to the stroke and improve their overall quality of life. Here are some ways rehabilitation can assist stroke patients:<br><br>1. Physical therapy: This helps patients relearn basic motor skills like walking, standing, and maintaining balance. Physical therapists use exercises, stretches, and assistive devices to improve strength, coordination, and mobility.<br><br>2. Occupational therapy: Occupational therapists work on improving the patient's ability to perform daily living activities like bathing, dressing, eating, and grooming. They may use adaptive equipment and techniques to compensate for impaired functions.<br><br>3. Speech and language therapy: Strokes can affect a person's ability to speak, understand language, and swallow. Speech and language therapists help patients regain these abilities through exercises and strategies.<br><br>4. Cognitive rehabilitation: Some strokes can impair cognitive functions like memory, attention, and problem-solving. Cognitive rehabilitation aims to retrain these skills through various exercises and techniques.<br><br>5. Psychological support: Strokes can lead to emotional challenges like depression, anxiety, and frustration. Counseling and support groups can help patients cope with the psychological impact of their condition.

	6. Education and training: Rehabilitation also involves educating patients and their families about stroke, its effects, and how to manage the condition effectively.<br><br>The benefits of rehabilitation for stroke patients include improved functional abilities, increased independence, better quality of life, and reduced risk of complications like muscle contractures or pressure sores. Early and intensive rehabilitation has been shown to lead to better outcomes and recovery.
I was recently discharged from hospital after being treated for stroke. The doctor says that I need to go back for rehabilitation on a weekly basis. How long does it usually last?	The duration of stroke rehabilitation can vary significantly depending on several factors, including the severity of the stroke, the areas of the brain affected, the individual's age, overall health, and rate of recovery. However, here's a general overview of what you can expect:<br><br>1. Inpatient rehabilitation:<br>- This typically occurs immediately after being discharged from the hospital for the stroke.<br>- It involves intensive therapy sessions (physical, occupational, and speech therapy) for several hours per day, 5-7 days a week.<br>- The length of stay can range from a few weeks to a few months, depending on the individual's progress and goals.<br><br>2. Outpatient rehabilitation:<br>- This is the stage you are currently in, where you will be attending therapy sessions on an outpatient basis, usually 1-3 times per week.<br>- Outpatient rehabilitation can last anywhere from several months to over a year, depending on the individual's needs and progress.<br>- The intensity and frequency of therapy sessions may be gradually reduced as you regain more function and independence.<br><br>It's important to note that stroke recovery is a highly individualized process, and the duration of rehabilitation can vary greatly from

	person to person. Some individuals may recover more quickly, while others may require longer-term or even lifelong therapy and support.<br><br>Your doctor and rehabilitation team will closely monitor your progress and adjust your therapy plan accordingly. It's crucial to actively participate in the rehabilitation process, follow the prescribed exercises and recommendations, and communicate openly with your healthcare team about your goals and concerns.<br><br>Remember, stroke recovery is a journey, and with dedication, perseverance, and the right support, many individuals can regain significant function and improve their quality of life.

Gemini responses:

-Prevention

COT	Responses
My dad recently suffered a stroke. I'm not really sure what caused this. Can you give me some ideas?	I am sorry to hear about your dad. There are two main causes of stroke:<br><br>● **Ischemic stroke:** This is the most common type of stroke, accounting for about 87% of all strokes. It is caused by a blood clot that blocks blood flow to the brain. The clot can form in an artery in the brain itself or travel to the brain from another part of the body, such as the heart.

- **Hemorrhagic stroke:** This type of stroke occurs when a blood vessel in the brain bursts or leaks. This can happen due to high blood pressure, a weakened blood vessel, or an aneurysm (a bulge in a blood vessel wall).

Several factors can increase the risk of stroke, including:

- High blood pressure
- High cholesterol
- Diabetes
- Smoking
- Obesity
- A family history of stroke
- Certain medical conditions, such as atrial fibrillation (irregular heartbeat) and sleep apnea

If you are concerned about your dad's risk of stroke, it is important to talk to his doctor. They can assess his risk factors and recommend ways to reduce his risk.

	Here are some resources that you may find helpful:<br><br>• National Stroke Association: https://www.stroke.org/en/<br>• American Stroke Association: https://www.stroke.org/en/
My parents recently suffered a stroke. Does it mean that I will have one too?	A family history of stroke does increase your risk, but it doesn't mean you will definitely have one. There are many risk factors for stroke, and some are controllable. Here's a breakdown:<br><br>• **Uncontrollable risk factors:** Age (risk increases with age), race (African Americans and Hispanics are at higher risk), family history.<br>• **Controllable risk factors:** High blood pressure, high cholesterol, diabetes, obesity, physical inactivity, smoking, unhealthy diet, sleep apnea.<br><br>By focusing on healthy lifestyle choices, you can significantly reduce your risk of stroke. This includes:<br><br>• Eating a healthy diet low in saturated fat, sodium, and added sugar<br>• Getting regular exercise

	- Maintaining a healthy weight - Managing stress - Not smoking - Following your doctor's recommendations for managing any underlying health conditions, like high blood pressure or diabetes If you're concerned about your risk, talk to your doctor. They can assess your individual risk factors and create a personalized plan to help you stay healthy.
My dad recently suffered a stroke. Maybe by changing my lifestyle, I can reduce the risk of stroke. Do you have any ideas?	I'm sorry to hear about your dad. There are definitely lifestyle changes you can make to reduce your risk of stroke. Here are some key areas to focus on: **Diet:** A healthy diet can significantly reduce your risk factors for stroke. Focus on fruits, vegetables, whole grains, and lean protein. Limit unhealthy fats, processed foods, and added sugar. Reducing salt intake is especially important, as high blood pressure is a major risk factor for stroke.

	● **Exercise:** Regular physical activity helps keep your heart healthy and lowers blood pressure and cholesterol. Aim for at least 30 minutes of moderate-intensity exercise most days of the week.<br>● **Weight management:** Being overweight or obese increases your risk of stroke. Shedding extra pounds can significantly improve your health.<br>● **Smoking cessation:** Smoking is a major risk factor for stroke. Quitting smoking is one of the best things you can do for your health.<br>● **Alcohol moderation:** Limit alcohol consumption. Excessive alcohol intake can raise blood pressure and increase your risk of stroke.<br>● **Stress management:** Chronic stress can contribute to unhealthy habits and high blood pressure. Find healthy ways to manage stress, such as exercise, relaxation techniques, or spending time in nature.<br>● **Regular checkups:** See your doctor regularly to monitor your blood pressure, cholesterol, and blood sugar levels. These are all important risk factors for stroke.<br><br>It's important to note that these are general recommendations, and it's always best to talk to your doctor about the best approach

	for you. They can consider your individual risk factors and create a personalized plan to help you reduce your risk of stroke.<br><br>Here are some resources that you may find helpful:<br><br>• American Stroke Association: https://www.stroke.org/en/<br>• Centers for Disease Control and Prevention: https://www.cdc.gov/stroke/about/index.html
My dad recently suffered a stroke. Perhaps I can prevent this by doing more exercise. Do you have any ideas?	I'm sorry to hear about your dad's stroke. While exercise is a great way to improve your overall health and can help reduce the risk of stroke, it's important to speak with a doctor before starting a new exercise program, especially if you have a family history of stroke.<br><br>Here's why talking to a doctor is important:<br><br>• **They can advise on a safe exercise plan:** They'll consider any underlying health conditions you may have and recommend exercises suitable for your fitness level.<br>• **They can help you manage existing conditions:** Exercise can be very beneficial for conditions like high blood pressure and high cholesterol, which are risk factors for stroke. A doctor can help you create an exercise plan that works alongside your

	current medications.<br><br>In the meantime, you can find general information about stroke prevention through exercise online at resources from reputable health organizations like the Stroke Association https://healthcare.utah.edu/healthfeed/2022/06/top-five-steps-preventing-stroke and the American Heart Association https://www.heart.org/en/healthy-living/fitness/fitness-basics/aha-recs-for-physical-activity-in-adults.<br><br>Again, I am sorry to hear about your dad, and I hope he recovers fully.
My dad recently suffered a stroke. Perhaps I can be healthier by changing the way I eat. Can taking fish oil or other health supplements help?	I'm sorry to hear about your dad's stroke. Diet can definitely play a role in stroke recovery and reducing the risk of future strokes. Here's what we know about fish oil and stroke recovery:<br><br>• **Fish oil contains omega-3 fatty acids:** These have some promise for stroke recovery, with research suggesting potential benefits like reducing inflammation and improving brain function after a stroke [1].<br>• **The evidence for fish oil is not conclusive:** More research is needed to determine the definitive impact of fish oil on stroke recovery in humans [2, 3].

	**Here's what to do:**<br><br>• **Talk to your dad's doctor:** They can advise on the best course of action for his specific recovery plan, including dietary changes and any potential supplements.<br>• **Consider a heart-healthy diet:** This emphasizes fruits, vegetables, whole grains, and lean protein, all of which can benefit your health and potentially aid your dad's recovery<br>• **Omega-3s from food sources:** You can increase your omega-3 intake through fatty fish like salmon, tuna, and mackerel.<br><br>**For reliable information on stroke recovery:**<br><br>• American Stroke Association: https://www.stroke.org/en/<br><br>Remember, I can't provide medical advice. It's important to consult with a doctor to get personalized recommendations for your dad and yourself.

-Diagnosis

<table>
<tr><th>COT</th><th>Responses</th></tr>
<tr><td>I’ve been having headaches quite often these days, especially on cold days. They seem to be getting worse with time. Could it be a sign of stroke?</td><td>It's definitely understandable to be worried about stroke, especially if you're experiencing worsening headaches. However, there are other more likely explanations for headaches that get worse with cold weather, such as:

● Sinus headaches: Cold weather can cause the sinuses to become inflamed due to changes in air pressure and dryness. This inflammation can lead to headaches with facial pressure and congestion.
● Migraines: Changes in barometric pressure, a common occurrence with cold fronts, can trigger migraines in some people.
● Tension headaches: Cold weather can also lead to muscle tension in the head and neck, which can contribute to tension headaches.

Here are some signs of stroke to be aware of, which are typically different from headaches:

● Sudden and severe headache, often described as the "worst headache of my life"
● Weakness or numbness on one side of the body
● Difficulty speaking or understanding speech
● Vision problems in one or both eyes</td></tr>
</table>

	• Dizziness or trouble walking<br><br>If you are experiencing any of these symptoms, it's important to seek immediate medical attention.<br><br>For your regular headaches, it would be best to consult with a doctor to get a proper diagnosis and discuss treatment options. They can help you determine the cause of your headaches and recommend the best course of action. In the meantime, keeping a headache diary to track the frequency, severity, and any potential triggers (like cold weather) can be helpful for your doctor.
I woke up this morning with my hand feeling numb. After a while, I noticed that my neck was sort of stiff too. Could these be the symptoms of a stroke?	Numbness in the hand and neck stiffness can be symptoms of a stroke, especially if they appeared suddenly. However, it's important to note that there are other possible explanations for these symptoms as well.<br><br>Here's why a stroke is a possibility:<br><br>• Strokes can cause numbness on one side of the body, and sometimes that numbness can affect the hands.<br>• Neck stiffness can also occur with a stroke, especially if the stroke affects the brainstem.<br><br>Here are some other reasons why you might be experiencing these symptoms:

	• Pinched nerve in your neck<br>• Poor sleeping position<br>• Repetitive stress injury<br><br>**Here's what to do:**<br><br>• Pay attention to other symptoms. Classic stroke symptoms include sudden weakness or numbness in one side of the face, arm, or leg, trouble speaking or understanding speech, confusion, drooping face, sudden trouble seeing, dizziness, or loss of balance. If you experience any of these other symptoms, call 911 immediately.<br>• If you are only experiencing numbness in your hand and neck stiffness, and the symptoms are not severe, you can monitor them for a while. If the symptoms improve within a few hours, it's likely not a stroke. However, if the symptoms worsen, or if you are concerned, it's always best to err on the side of caution and seek medical attention.<br><br>**It is important to remember that I am not a medical professional and cannot diagnose you. If you are concerned that you may be having a stroke, call 911 immediately.**

I’ve been feeling dizzy for several weeks already. It seems to be getting worse with time. Could it be a stroke?	Dizziness for several weeks that's worsening is definitely a cause for concern, but it doesn't necessarily mean a stroke. There are many reasons for dizziness, some more serious than others. Here are some possibilities:<br><br>• Inner ear problems like benign paroxysmal positional vertigo (BPPV) or Meniere's disease [WebMD inner ear dizziness]<br>• Dehydration [WebMD dizziness]<br>• Low blood sugar [WebMD dizziness]<br>• Medication side effects [WebMD dizziness]<br>• Anxiety or stress [Mayo Clinic dizziness]<br><br>A stroke is a serious medical emergency, so it's important to get checked out by a doctor to rule it out, especially if you're experiencing other symptoms like:<br><br>• Sudden, severe headache<br>• Chest pain<br>• Difficulty breathing<br>• Numbness or paralysis

- Fainting
- Double vision
- Rapid or irregular heartbeat
- Confusion or slurred speech
- Difficulty walking
- Ongoing vomiting
- Seizures
- Hearing loss
- Facial droop

If you are experiencing any of these along with dizziness, call emergency services immediately.

For non-emergency cases, make an appointment with your doctor as soon as possible. They can do a physical exam and ask you questions about your medical history and symptoms to determine the cause of your dizziness.

In the meantime, there are some things you can do to try to reduce your dizziness, such as staying hydrated, avoiding alcohol and

	caffeine, and getting enough sleep. You should also avoid straining yourself or lifting heavy objects.
My blood pressure has been higher than usual these days. So high that my head actually hurts even when I go for a walk. Could it be a stroke?	It's definitely a cause for concern that your blood pressure is high and you're experiencing headaches. While headaches aren't a typical symptom of high blood pressure, they can occur, especially if your blood pressure readings are very high. However, there are other reasons for headaches and high blood pressure, and a stroke is a serious possibility that needs to be ruled out.<br><br>**Here's what to do:**<br><br>1. **Don't ignore it.** Call your doctor and explain your symptoms. They may want you to come in right away or monitor your blood pressure at home for a bit.<br>2. **If your blood pressure is very high (above 180/120mmHg)**, or you're experiencing other concerning symptoms like chest pain, blurred vision, or difficulty breathing, **go to the emergency room immediately**. This could be a sign of a hypertensive crisis, which is a medical emergency.<br><br>Here are some resources that you might find helpful:

	• American Heart Association on High Blood Pressure: https://www.heart.org/en/health-topics/high-blood-pressure<br>• National Institute of Neurological Disorders and Stroke on Stroke: https://www.ninds.nih.gov/
Since my dad's stroke, I've been alert for any signs.I understand that recognizing symptoms early can be crucial. Could you list the exact symptoms?	Recognizing the signs of stroke early is crucial because it can significantly improve treatment outcomes and potentially minimize long-term complications. Here are the main symptoms to watch for according to the National Institute on Aging https://www.ninds.nih.gov/health-information/stroke/signs-and-symptoms:<br><br>• **Sudden numbness or weakness in the face, arm, or leg, especially on one side of the body.** This can manifest as difficulty raising both arms or having one side of the face droop.<br>• **Sudden confusion, trouble speaking, or understanding speech.** This could include slurred speech, difficulty finding words, or struggling to understand what others are saying.<br>• **Sudden problems seeing in one or both eyes.** This can include blurred vision, blackened vision in one eye, or double vision.<br>• **Sudden dizziness, loss of balance or coordination, or trouble walking.** A person having a stroke may have difficulty walking or maintaining balance.

	**Additional symptoms to be aware of include:**<br><br>● Sudden severe headache with no known cause<br>● Difficulty swallowing<br><br>**It's important to remember that not everyone will experience all of these symptoms, and some symptoms may be milder than others. However, if you notice any of these signs in yourself or someone else, call 911 immediately.** Early action can save brain cells and significantly improve the chances of a full recovery.

-Treatment

COT	Responses
I've just been diagnosed with stroke. The doctor says that there is severe blood plaque blockage. Are there any medications or treatments that can	I am sorry to hear about your stroke diagnosis. Blood plaque blockage, also known as atherosclerosis, is a serious condition. There are medications and treatments that can help prevent future strokes and manage your condition, but unfortunately there isn't a medication that can directly dissolve existing plaque buildup.<br><br>Here are some medications that your doctor might prescribe:

help clear it?	● Statins: These are the most common cholesterol-lowering medications. They work by blocking a substance your liver needs to make cholesterol.<br>● Aspirin: This medication helps thin the blood and prevent blood clots.<br>● Other cholesterol-lowering medications: If statins don't lower your LDL (bad) cholesterol enough, your doctor may prescribe other medications, such as ezetimibe (Zetia) or PCSK9 inhibitors.<br>● Blood pressure medications: High blood pressure can damage your arteries and make atherosclerosis worse. There are many different types of blood pressure medications, and your doctor will choose the one that's right for you.<br><br>In some cases, medical procedures might be necessary to address the blockage:<br><br>● Angioplasty and stenting: This procedure involves inserting a thin tube (catheter) into a blocked artery and inflating a tiny balloon to open it up. A stent, which is a small mesh tube, may be inserted to help keep the artery open.<br>● Bypass surgery: In this procedure, the surgeon creates a new pathway for blood to flow around the blocked artery. This is typically done with a blood vessel from another part of your body.<br><br>Lifestyle changes are also crucial in managing plaque buildup:

	• Healthy diet: Eating a healthy diet that's low in saturated and unhealthy fats, cholesterol, and sodium can help lower your LDL cholesterol and blood pressure.<br>• Exercise: Regular exercise can help lower your LDL cholesterol and blood pressure and improve your overall cardiovascular health.<br>• Weight management: If you're overweight or obese, losing weight can help lower your LDL cholesterol and blood pressure.<br>• Smoking cessation: Smoking damages your arteries and increases your risk of stroke. Quitting smoking is one of the best things you can do for your health.<br><br>Remember, I am not a medical professional and this information does not constitute medical advice. Please consult with your doctor about the best course of treatment for you.
I’ve just been diagnosed with acute stroke. Perhaps with the right treatment I will be able to get better.	I'm sorry to hear that you've been diagnosed with an acute stroke. This is a serious medical condition and getting the right treatment as soon as possible is crucial. **I cannot give any medical advice**. However, I can tell you that there are effective treatments available for stroke, and the sooner you receive treatment, the better your chances of recovery.

Do you have any suggestions?	Here's some general information:<br><br>● The most common treatment for ischemic stroke is a clot-busting medication called tissue plasminogen activator (tPA). This medication is most effective if given within 3-4.5 hours of stroke symptom onset.<br>● There are also minimally invasive surgical procedures that can be used to remove blood clots from blocked arteries.<br><br>**Call emergency services immediately** or proceed to the nearest emergency room. Explain to medical professionals that you suspect you are having a stroke and provide details about when your symptoms began.<br><br>Here are some resources that you may find helpful:<br><br>● The National Stroke Association https://www.stroke.org/en/<br>● The American Stroke Association https://www.stroke.org/en/<br>● The National Institutes of Health Stroke page https://www.nhlbi.nih.gov/
I've just been prescribed some medication for	I can't give medical advice, and how long you need to take stroke medication depends entirely on your specific situation and the

stroke. However, there seem to be some potential side effects. How long do I need to keep taking them?	doctor's prognosis.<br><br>There are different types of stroke medication, and each has its own risk-reward profile. Here's some general information:<br><br>● **Blood thinners** like warfarin or heparin are prescribed to prevent future strokes, but they increase bleeding risk. You might need close monitoring and regular blood tests.<br>● **Antiplatelet medications** like aspirin or clopidogrel also prevent clots but with a lower bleeding risk.<br><br>The best course of action is to discuss your concerns with the doctor who prescribed the medication. They can explain the potential side effects in detail, weigh them against the benefits, and determine an appropriate treatment plan, including how long you need to take the medication.
I've just been diagnosed with a severe stroke in the brain. Is it possible for the damaged area to recover?	I'm sorry to hear about your stroke diagnosis. While it's true that some areas of the brain can't regenerate after a stroke, there's still a good chance for recovery. The brain has a remarkable ability called neuroplasticity, which allows it to reorganize and form new connections between nerve cells https://www.ncbi.nlm.nih.gov/books/NBK557811/. This can help compensate for the

	damaged area and restore function.<br><br>Here's some information on stroke recovery:<br><br>• **Timeline:** Most improvement happens in the first 3 months, but recovery can continue for months or even years after a stroke https://www.hopkinsmedicine.org/health/conditions-and-diseases/stroke/stroke-recovery-timeline.<br>• **Factors affecting recovery:** Severity of the stroke, how quickly you received treatment, and the intensity of rehabilitation all play a role https://www.mayoclinic.org/diseases-conditions/stroke/in-depth/stroke-rehabilitation/art-20045172.<br>• **Rehabilitation:** This is crucial for regaining function and includes physiotherapy, speech therapy, and occupational therapy https://www.nhs.uk/conditions/stroke/recovery/.<br><br>Here are some resources that you might find helpful:<br><br>• **National Stroke Association:** https://www.stroke.org/en/<br>• **American Stroke Association:** https://www.stroke.org/en/<br><br>Remember, it's important to talk to your doctor about your specific case and what kind of recovery you can expect. They can

	create a personalized rehabilitation plan to help you regain as much independence as possible.
I am about to undergo surgery for stroke. What should I expect in the operating room?	The operating room can seem like a stressful environment, but knowing what to expect can help ease some anxiety. Here's a general idea of what to expect:<br><br>**Before surgery:**<br><br>● **The surgical team:** You'll be greeted by a team of medical professionals, including surgeons, nurses, and anesthesiologists.<br>● **Anesthesia:** The anesthesiologist will discuss anesthesia options (general vs. local) and monitor your vitals.<br><br>**During surgery:**<br><br>● **Monitoring:** You'll have an IV inserted for fluids and medication. Machines will monitor your heart rate, blood pressure, and oxygen levels.<br>● **The procedure:** The exact procedure will depend on the nature of your stroke, but the team will ensure you're comfortable and unaware of the surgery itself.

	**After surgery:**<br><br>● **Recovery room:** Once the surgery is complete, you'll be transferred to a recovery room for close monitoring until you wake up from anesthesia.<br>● **Transfer to room:** When stable, you'll be transferred to a hospital room for continued recovery.<br><br>Remember, this is a general outline. It's important to talk to your doctor about the specifics of your surgery and what to expect. They can address any concerns you have and answer questions about the procedure itself.

-Recovery

COT	Responses
I am trying to recover from stroke. I wonder if rehabilitation and hyperbaric oxygen therapy can help restore brain	Absolutely! Stroke rehabilitation is the cornerstone of regaining function after a stroke. It is a personalized program that helps you relearn lost skills and improve your ability to perform daily activities. Rehabilitation typically involves a team of healthcare professionals, including physical therapists, occupational therapists, speech-language pathologists, and cognitive therapists.

function.	Hyperbaric oxygen therapy (HBOT) is a treatment that involves breathing pure oxygen in a pressurized chamber. While research on its effectiveness for stroke recovery is ongoing, some studies suggest that HBOT may offer benefits such as:<br><br>● Increased oxygen supply to the brain<br>● Reduced brain swelling<br>● Improved cognitive function<br>● Enhanced motor function<br><br>**Here's a breakdown of how each can help restore brain function:**<br><br>● **Rehabilitation:** Through repetitive practice and targeted exercises, rehabilitation helps the brain create new neural pathways to compensate for the damaged areas. This process, called neuroplasticity, is essential for regaining lost skills and functions.<br>● **Hyperbaric Oxygen Therapy (HBOT):** By delivering extra oxygen to the brain, HBOT may help reduce inflammation, promote healing, and stimulate the growth of new blood vessels. This can potentially improve the brain's environment and support its recovery process.

	**It is important to note that:**<br><br>• HBOT is not a cure for stroke, but it may be a helpful adjunct to traditional rehabilitation.<br>• HBOT is not covered by all insurance plans, and it can be expensive.<br>• There can be some side effects associated with HBOT, such as ear pain and claustrophobia.<br><br>**If you are considering HBOT, it is important to talk to your doctor to see if it is right for you.** They can help you weigh the risks and benefits and determine if it is an appropriate addition to your rehabilitation plan.<br><br>Here are some resources that you may find helpful:<br><br>• American Stroke Association: https://www.stroke.org/en/<br>• National Institute of Neurological Disorders and Stroke: https://www.ninds.nih.gov/<br>• Undersea and Hyperbaric Medical Society: https://www.uhms.org/
I am trying different methods to recover from a stroke. I	Stroke rehabilitation is absolutely crucial for regaining function after a stroke, and it should be the cornerstone of your recovery

wonder if rehabilitation and intravenous laser irradiation of blood help restore brain function.	plan. Rehabilitation helps the brain create new neural pathways to compensate for the damaged areas, a process called neuroplasticity. This can help you regain movement, speech, thinking, and daily living skills.<br><br>Intravenous laser irradiation of blood (LIIL) is an emerging therapy that's still under investigation for stroke recovery. Early studies show promise, but more research is needed to determine its effectiveness and safety for humans. Here's a breakdown of what we know so far:<br><br>• **LIIL procedure:** It involves irradiating your blood with laser light.<br>• **Potential benefits:** It's thought to work by improving blood flow and reducing inflammation in the brain, which could promote healing and recovery.<br><br>**While LIIL shows promise, it's important to consider:**<br><br>• **Limited research in humans:** More studies are needed to confirm its effectiveness and safety for stroke recovery.<br>• **Talk to your doctor:** If you're considering LIIL, discuss it with your doctor to see if it's right for you. They can help you weigh the risks and benefits and determine if it's an appropriate addition to your rehabilitation plan.

	Here are some resources that you may find helpful:<br><br>• American Stroke Association: https://www.stroke.org/en/<br>• National Institute of Neurological Disorders and Stroke: https://www.ninds.nih.gov/
I am trying to recover from stroke. I wonder if rehabilitation and transcranial magnetic stimulation aid in restoring brain function.	Yes, both rehabilitation and transcranial magnetic stimulation (TMS) are showing promise in aiding post-stroke recovery.<br><br>**Stroke rehabilitation** is the cornerstone of treatment after a stroke. It is an individualized program designed to help you regain movement, speech, language, and other abilities lost due to the stroke. Rehabilitation typically includes physical therapy, occupational therapy, speech therapy, and cognitive therapy.<br><br>**Transcranial magnetic stimulation (TMS)** is a non-invasive technique that uses magnetic fields to stimulate the brain. It is a relatively new treatment for stroke, but research suggests that it may be helpful in improving motor function, language, and neglect (a condition that causes people to be unaware of one side of their body).<br><br>Here's a summary of the benefits of each:

	**Rehabilitation**<br><br>• Improves motor function<br>• Improves speech and language<br>• Improves cognitive function<br>• Increases independence<br>• Reduces the risk of complications<br><br>**TMS**<br><br>• May improve motor function in the affected limb<br>• May improve language function<br>• May improve neglect<br><br>**How they work together**<br><br>Rehabilitation and TMS can be used together to enhance stroke recovery. Rehabilitation helps the brain to relearn how to control

	the affected parts of the body, while TMS may help to make the brain more receptive to rehabilitation.<br><br>**If you are interested in learning more about stroke rehabilitation or TMS, talk to your doctor.** They can help you decide if these treatments are right for you.<br><br>Here are some resources that you may find helpful:<br><br>• The American Stroke Association: https://www.stroke.org/en/<br>• The National Institute of Neurological Disorders and Stroke: https://www.ninds.nih.gov/
I would like to know how useful rehabilitation is for stroke patients. What assistance does doing so provide?	Rehabilitation is very useful for stroke patients. It can help them regain independence and improve their quality of life. Here are some of the ways that rehabilitation can assist stroke patients:<br><br>• **Physical therapy** can help patients improve their movement, balance, and coordination. This can help them with activities such as walking, dressing, and bathing.<br>• **Occupational therapy** can help patients relearn daily living skills, such as cooking, cleaning, and driving.<br>• **Speech-language therapy** can help patients improve their communication skills. This can include helping them to speak

	more clearly, understand speech better, and swallow safely.<br>• **Cognitive therapy** can help patients improve their thinking skills, such as memory, problem-solving, and attention.<br><br>Rehabilitation can also help to reduce stroke patients' risk of complications, such as pneumonia and blood clots. It can also help them to cope with the emotional effects of stroke, such as depression and anxiety.<br><br>The American Stroke Association recommends that all stroke patients receive rehabilitation. The amount and type of rehabilitation that a patient needs will vary depending on the severity of their stroke and their individual goals.<br><br>If you are a stroke survivor, or if you know someone who is, talk to your doctor about rehabilitation. Rehabilitation can help you or your loved one to live a more independent and fulfilling life.
I was recently discharged from hospital after being treated for stroke. The doctor says that I need to go back for rehabilitation on a	Stroke rehabilitation is highly individualized, and there's no one-size-fits-all answer for duration. However, here's a general idea:<br><br>• **Most improvement:** Typically happens within the first 3 months [Mayo Clinic]. This is when intensive rehab programs are common [Johns Hopkins Medicine].

weekly basis. How long does it usually last?	• **Long-term rehab:** Even after those initial months, some form of rehabilitation is often recommended, potentially for months or even years [Mayo Clinic].<br><br>Here are some factors affecting rehab duration:<br><br>• **Stroke severity:** More severe strokes tend to require longer rehabilitation periods [Mayo Clinic].<br>• **Initial treatment:** Faster medical intervention after a stroke can improve recovery chances [Johns Hopkins Medicine].<br>• **Rehab intensity:** More frequent and intensive therapy sessions can lead to faster progress [National Institutes of Health].<br><br>The good news is that continued progress can be made even after a year [Mayo Clinic].<br><br>**Moving forward:**<br><br>• Discuss a specific rehab plan with your doctor, including frequency and duration.<br>• Inquire about the possibility of progress evaluations to gauge your recovery.<br><br>Remember, consistency is key in rehab. By following your doctor's recommendations, you're giving yourself the best chance for a

	full recovery.

GPT responses:

-Prevention

TOT	Responses
My dad recently suffered a stroke. Now I am really worried about myself too. What if I suddenly get a stroke and die? I need to know how to prevent this from happening.	I'm sorry to hear about your father's stroke, and it's understandable to be concerned about your own health. While it's not possible to eliminate all risk, there are several steps you can take to reduce your risk of having a stroke:<br><br>**Lifestyle Changes**<br><br>1. **Healthy Diet**:<br>○ Eat plenty of fruits and vegetables.<br>○ Choose whole grains over refined grains.<br>○ Limit intake of saturated fats, trans fats, and cholesterol.<br>○ Reduce salt (sodium) intake to help lower blood pressure.<br>○ Eat lean proteins, such as fish, poultry, beans, and nuts.

2. **Regular Exercise**:
   - Aim for at least 150 minutes of moderate-intensity aerobic activity or 75 minutes of vigorous-intensity activity each week.
   - Include muscle-strengthening activities on 2 or more days a week.
3. **Maintain a Healthy Weight**:
   - Being overweight increases your risk of stroke. A healthy diet and regular exercise can help you maintain a healthy weight.
4. **Stop Smoking**:
   - Smoking significantly increases the risk of stroke. Quitting smoking can greatly reduce your risk.
5. **Limit Alcohol Consumption**:
   - Drink in moderation. For women, this means no more than one drink per day, and for men, no more than two drinks per day.

Medical Management

1. **Manage Blood Pressure**:
   - High blood pressure is a major risk factor for stroke. Keep your blood pressure under control through diet, exercise, and medication if needed.
2. **Control Diabetes**:
   - If you have diabetes, manage your blood sugar levels. This can be done through diet, exercise, and medication.
3. **Monitor Cholesterol Levels**:
   - High cholesterol can lead to the buildup of plaques in blood vessels, increasing the risk of stroke. Manage your cholesterol through diet, exercise, and medication if necessary.
4. **Take Medications as Prescribed**:
   - If your doctor prescribes medications to control high blood pressure, diabetes, or cholesterol, take them as

	directed.<br><br>**Regular Check-ups**<br><br>● **Routine Health Screenings**:<br>○ Regular check-ups with your doctor can help identify and manage any health issues that could increase your risk of stroke.<br><br>**Awareness**<br><br>1. **Know the Signs of a Stroke**:<br>○ Be aware of the symptoms of a stroke (sudden numbness or weakness in the face, arm, or leg, especially on one side of the body; sudden confusion, trouble speaking, or understanding speech; sudden trouble seeing in one or both eyes; sudden trouble walking, dizziness, loss of balance, or lack of coordination; sudden severe headache with no known cause).<br>2. **Act Quickly**:<br>○ If you or someone else shows any signs of a stroke, call emergency services immediately. Time is critical in stroke treatment.<br><br>Taking these steps can help you significantly reduce your risk of stroke. It's also important to maintain a positive mindset and not let fear dominate your daily life. Regular medical check-ups and a healthy lifestyle are your best defenses against stroke.
My parents recently suffered a stroke. I've recently found	The fact that your parents have had a stroke can indicate an increased risk for you, but it does not mean you are certain to have one. Several factors contribute to stroke risk, including genetics, lifestyle, and underlying health conditions. Here are some key

an article on the internet saying that it is hereditary. Does it mean that I will get one too?	points to consider:<br><br>**1. Genetic Factors**<br><br>Family history can play a role in stroke risk, particularly if the stroke was caused by conditions that have a genetic component, such as hypertension, diabetes, or high cholesterol.<br><br>**2. Lifestyle Factors**<br><br>Your lifestyle choices significantly impact your stroke risk. Maintaining a healthy diet, regular exercise, avoiding smoking, and limiting alcohol consumption can reduce your risk.<br><br>**3. Medical Conditions**<br><br>Conditions like high blood pressure, diabetes, atrial fibrillation, and high cholesterol increase stroke risk. Managing these conditions with the help of healthcare professionals can reduce your risk.<br><br>**4. Regular Check-Ups**<br><br>Regular medical check-ups can help identify and manage risk factors early. Monitoring blood pressure, cholesterol levels, and blood sugar levels is crucial.<br><br>**5. Preventive Measures**<br><br>Following preventive measures, such as taking prescribed medications, maintaining a healthy weight, and managing stress, can further reduce your risk.<br><br>It’s important to discuss your concerns with a healthcare professional who can provide personalized advice based on your medical

	history and lifestyle. They can help you understand your specific risk and guide you on the best preventive measures to take.
My dad recently suffered a stroke. Perhaps it’s because he often stays up late for work. I seem to have the same problem too. I wonder how I can change my lifestyle to prevent this.	I’m sorry to hear about your parents. Stroke can indeed have a hereditary component, meaning that having a family history of stroke can increase your risk. However, it doesn't mean you will definitely have a stroke. Many factors contribute to stroke risk, including lifestyle, medical conditions, and genetics.<br><br>To reduce your risk, you can:<br><br>1. **Maintain a Healthy Diet**: Eat plenty of fruits, vegetables, and whole grains. Limit salt, sugar, and saturated fats.<br>2. **Exercise Regularly**: Aim for at least 150 minutes of moderate-intensity aerobic activity or 75 minutes of vigorous-intensity activity each week.<br>3. **Manage Blood Pressure**: Keep it within the recommended range.<br>4. **Control Diabetes**: If you have diabetes, manage your blood sugar levels.<br>5. **Avoid Smoking**: Smoking significantly increases your risk of stroke.<br>6. **Limit Alcohol**: Drink alcohol in moderation, if at all.<br>7. **Monitor Cholesterol Levels**: Keep your cholesterol in check.<br><br>It's also essential to regularly visit your doctor for check-ups and to discuss your specific risk factors. They can provide personalized advice and, if necessary, prescribe medications to help manage conditions like hypertension or high cholesterol.<br><br>If you have any specific concerns, it would be best to discuss them with your healthcare provider. They can give you more detailed information based on your personal health history and risk factors.
My dad recently suffered a stroke. I	I'm sorry to hear about your father's stroke. Regular physical activity is indeed an important factor in reducing the risk of stroke.

<table>
<tr>
<td>think it’s because he seldom exercises. What kinds of exercise can I do to decrease the risk of stroke?</td>
<td>Here are some types of exercises that can help:

Aerobic Exercises

Aerobic exercises are beneficial for cardiovascular health, which in turn can reduce the risk of stroke. Examples include:

● Walking: Brisk walking for 30 minutes a day can significantly improve heart health.
● Jogging or Running: For those who are more fit, jogging or running can provide more intense cardiovascular benefits.
● Cycling: Both stationary and outdoor cycling are excellent for heart health.
● Swimming: This is a low-impact exercise that can be particularly good for those with joint issues.

Strength Training

Building muscle through strength training can help improve overall health and support cardiovascular function.

● Weight Lifting: Using free weights or weight machines.
● Bodyweight Exercises: Push-ups, squats, lunges, and other exercises that use your body weight as resistance.

Flexibility and Balance Exercises

These exercises can help improve overall fitness and reduce the risk of falls, which can be crucial for stroke prevention and recovery.

● Yoga: Enhances flexibility, balance, and strength.
● Tai Chi: Focuses on slow, deliberate movements and balance, which can be especially beneficial for older adults.

High-Intensity Interval Training (HIIT)</td>
</tr>
</table>

	For those who are already physically active, HIIT can offer substantial cardiovascular benefits in a shorter amount of time. It involves short bursts of intense exercise followed by rest or low-intensity exercise.<br><br>**Daily Activity**<br><br>Incorporating more movement into your daily routine can also help:<br><br>● **Taking the stairs instead of the elevator**<br>● **Walking or biking for short trips instead of driving**<br>● **Doing household chores like gardening, cleaning, or washing the car**<br><br>**Tips for Safe Exercise**<br><br>● **Start Slowly**: If you or your father are not used to regular exercise, start with shorter, less intense sessions and gradually increase the duration and intensity.<br>● **Consult a Doctor**: Before starting any new exercise regimen, especially if there are existing health conditions, it's important to consult with a healthcare provider.<br>● **Stay Hydrated**: Drink plenty of water before, during, and after exercise.<br>● **Listen to Your Body**: Pay attention to how your body feels during and after exercise. If something doesn't feel right, stop and seek medical advice if necessary.<br><br>Regular exercise, along with a healthy diet, maintaining a healthy weight, and avoiding smoking, can significantly reduce the risk of stroke.
My dad recently suffered a stroke. I	I'm sorry to hear about your father's stroke. Making dietary changes can significantly reduce the risk of stroke. Here are some

have a feeling it's because he enjoys eating fast foods. How can I reduce the risk of stroke by changing my diet?	dietary recommendations to help:<br><br>**1. Eat More Fruits and Vegetables:**<br><br>● Aim for at least 5 servings of fruits and vegetables daily.<br>● Include a variety of colors to ensure a range of nutrients.<br><br>**2. Choose Whole Grains:**<br><br>● Opt for whole-grain bread, pasta, and cereals instead of refined grains.<br>● Brown rice, quinoa, and oats are good choices.<br><br>**3. Limit Saturated and Trans Fats:**<br><br>● Reduce intake of red and processed meats, butter, and full-fat dairy products.<br>● Avoid trans fats found in many fried and baked goods.<br><br>**4. Increase Healthy Fats:**<br><br>● Include sources of omega-3 fatty acids such as fish, flaxseeds, and walnuts.<br>● Use olive oil or canola oil instead of butter or margarine.<br><br>**5. Reduce Sodium Intake:**<br><br>● Limit processed and packaged foods that are high in sodium.<br>● Cook with herbs and spices instead of salt.<br><br>**6. Limit Added Sugars:**

	● Cut down on sugary drinks, desserts, and snacks.<br>● Read labels to identify hidden sugars in packaged foods.<br><br>**7. Eat Lean Protein:**<br><br>● Choose lean meats, poultry, fish, beans, and legumes.<br>● Consider plant-based protein sources like tofu and tempeh.<br><br>**8. Stay Hydrated:**<br><br>● Drink plenty of water throughout the day.<br>● Limit sugary drinks and high-caffeine beverages.<br><br>**9. Moderate Alcohol Consumption:**<br><br>● If you drink alcohol, do so in moderation.<br>● For men, this means up to two drinks per day, and for women, up to one drink per day.<br><br>**10. Plan and Prepare Meals:**<br><br>● Plan meals ahead to ensure they are balanced and healthy.<br>● Cook at home more often to control ingredients and portion sizes.<br><br>**Example Meal Plan:**<br><br>● **Breakfast:** Oatmeal topped with fresh berries and a sprinkle of nuts.<br>● **Lunch:** Grilled chicken salad with a variety of vegetables, drizzled with olive oil and lemon dressing.

	● **Dinner:** Baked salmon with quinoa and steamed broccoli.<br>● **Snacks:** Fresh fruit, yogurt, or a handful of almonds.<br><br>**Lifestyle Changes:**<br><br>In addition to dietary changes, other lifestyle changes such as regular physical activity, maintaining a healthy weight, managing stress, and not smoking can also reduce the risk of stroke.<br><br>Would you like more detailed recipes or specific dietary plans?

-Diagnosis

TOT	Responses
I’ve been having headaches quite often these days, especially on cold days. They seem to be getting worse with time. I’m really worried about my condition. Could it be a sign of stroke?	I'm sorry to hear that you've been experiencing frequent headaches. While headaches can be a symptom of various conditions, they are not typically a primary sign of stroke. However, since you have a history of stroke, it's important to take any new or worsening symptoms seriously.<br><br>Common signs of a stroke include:<br><br>- Sudden numbness or weakness in the face, arm, or leg, especially on one side of the body<br>- Sudden confusion, trouble speaking, or difficulty understanding speech<br>- Sudden trouble seeing in one or both eyes<br>- Sudden trouble walking, dizziness, loss of balance, or lack of coordination<br>- Sudden severe headache with no known cause

	Given your concern and history, it would be best to consult with a healthcare professional as soon as possible to rule out any serious conditions and to address your headaches properly. Additionally, if you experience any of the stroke symptoms mentioned above, seek emergency medical attention immediately.<br><br>In the meantime, try to note any patterns or triggers for your headaches, such as specific weather conditions, activities, foods, or stress levels, as this information can be helpful for your doctor.
I woke up this morning with my hand feeling really numb. I also think that my neck is sort of stiff too. Could these be the symptoms of a stroke?	Numbness in the hand and a stiff neck can be concerning, but they are not specific enough on their own to definitively indicate a stroke. However, it's crucial to be vigilant and consider the possibility, especially given your history. Here are some key stroke symptoms to watch for:<br><br>1. **Sudden numbness or weakness** in the face, arm, or leg, particularly on one side of the body.<br>2. **Sudden confusion**, trouble speaking, or difficulty understanding speech.<br>3. **Sudden trouble seeing** in one or both eyes.<br>4. **Sudden trouble walking**, dizziness, loss of balance, or lack of coordination.<br>5. **Sudden severe headache** with no known cause.<br><br>If you experience any of these symptoms, it's important to seek immediate medical attention. It's always better to err on the side of caution when dealing with potential stroke symptoms. If you are concerned about your current symptoms, I strongly recommend contacting a healthcare professional or going to the emergency room as soon as possible.
I’ve been feeling dizzy for several weeks already. To	I'm sorry to hear that you're feeling unwell. While dizziness and headaches can be symptoms of various conditions, including a stroke, it's important not to jump to conclusions without proper medical evaluation. Common stroke symptoms often include:

make matters worse, the headaches seem to be getting worse with time. Do you think I have a stroke?	- Sudden numbness or weakness in the face, arm, or leg, especially on one side of the body.<br>- Sudden confusion, trouble speaking, or understanding speech.<br>- Sudden trouble seeing in one or both eyes.<br>- Sudden trouble walking, dizziness, loss of balance, or coordination.<br>- Sudden severe headache with no known cause.<br><br>Given your symptoms and your history, it's crucial to seek medical attention as soon as possible. A healthcare professional can provide a proper diagnosis and recommend the appropriate treatment. Please contact your doctor or visit an emergency room to get evaluated.
My blood pressure has been higher than usual these days. So high that my head hurts even when I walk around. Could it be a stroke?	I'm really sorry to hear that you're experiencing these symptoms. Elevated blood pressure and headaches can be concerning, especially given your history. While high blood pressure can increase the risk of stroke, it's essential to be aware of other stroke symptoms. These can include:<br><br>- Sudden numbness or weakness in the face, arm, or leg, particularly on one side of the body<br>- Sudden confusion, trouble speaking, or difficulty understanding speech<br>- Sudden trouble seeing in one or both eyes<br>- Sudden trouble walking, dizziness, loss of balance, or lack of coordination<br>- Sudden severe headache with no known cause<br><br>Given your symptoms and medical history, it's crucial to seek medical attention immediately. High blood pressure and headaches alone don't confirm a stroke, but they do warrant prompt evaluation by a healthcare professional. It's better to be safe and get a thorough assessment as soon as possible.

<table>
<tr><td>Ever since my dad had a stroke, I've been constantly on the lookout for the signs. Can you tell me exactly what the symptoms are? Perhaps this will make me feel more at ease.</td><td>Absolutely, understanding the symptoms can help you feel more prepared. Here are the main symptoms of a stroke, summarized by the acronym FAST:

1. Face drooping: One side of the face may droop or feel numb. Ask the person to smile and see if their smile is uneven.
2. Arm weakness: One arm may feel weak or numb. Ask the person to raise both arms and see if one drifts downward.
3. Speech difficulty: Speech may be slurred or hard to understand. Ask the person to repeat a simple sentence and check for any abnormalities in their speech.
4. Time to call emergency services: If any of these symptoms are present, even if they go away, call emergency services immediately.

Other possible symptoms include:

● Sudden confusion or trouble understanding: This can manifest as difficulty in understanding simple statements or responding appropriately.
● Sudden trouble seeing: This can include blurred vision, double vision, or complete loss of vision in one or both eyes.
● Sudden trouble walking: This includes dizziness, loss of balance or coordination, and sudden falls.
● Sudden severe headache: This is often described as the worst headache of one's life and can come on with no known cause.

If you notice any of these signs, it's crucial to act quickly. Immediate medical attention is essential for the best possible outcome.</td></tr>
</table>

-Treatment

TOT	Responses
I’ve just been diagnosed with	I'm sorry to hear about your diagnosis. It's important to follow your doctor's advice closely. There are several treatments and

stroke. The doctor told me that there is severe blood plaque blockage. I think that this is way more serious than I thought. Are there any medications or treatments that can help?	medications that can help manage stroke and reduce the risk of future strokes, especially in the presence of severe blood plaque blockage:<br><br>**Medications**<br><br>1. **Antiplatelet Drugs**:<br>○ **Examples**: Aspirin, Clopidogrel<br>○ **Purpose**: Help prevent blood clots from forming.<br>2. **Anticoagulants**:<br>○ **Examples**: Warfarin, Dabigatran<br>○ **Purpose**: Reduce the blood's ability to clot.<br>3. **Statins**:<br>○ **Examples**: Atorvastatin, Simvastatin<br>○ **Purpose**: Lower cholesterol levels and stabilize plaque.<br>4. **Blood Pressure Medications**:<br>○ **Examples**: ACE inhibitors, Beta-blockers<br>○ **Purpose**: Control high blood pressure, which is a major risk factor for stroke.<br>5. **Diabetes Medications**:<br>○ **Purpose**: Manage blood sugar levels if you have diabetes.<br><br>**Procedures and Surgeries**<br><br>1. **Carotid Endarterectomy**:<br>○ **Purpose**: Surgery to remove plaque from the carotid arteries in the neck.<br>2. **Angioplasty and Stenting**:<br>○ **Purpose**: Procedure involves inflating a small balloon inside the artery and placing a stent to keep it open.

	**Lifestyle Changes**<br><br>1. **Healthy Diet**:<br>○ **Recommendations**: Eat a diet low in saturated fats, trans fats, and cholesterol, and high in fruits, vegetables, and whole grains.<br>2. **Regular Exercise**:<br>○ **Recommendations**: Engage in physical activity to improve cardiovascular health.<br>3. **Smoking Cessation**:<br>○ **Recommendations**: Quit smoking to significantly reduce your risk of stroke.<br>4. **Limiting Alcohol**:<br>○ **Recommendations**: Reduce alcohol intake to moderate levels.<br><br>**Rehabilitation**<br><br>1. **Physical Therapy**:<br>○ **Purpose**: Help regain strength and coordination.<br>2. **Occupational Therapy**:<br>○ **Purpose**: Help relearn daily activities and improve fine motor skills.<br>3. **Speech Therapy**:<br>○ **Purpose**: Improve ability to speak or understand speech if affected by the stroke.<br><br>**Regular Monitoring and Follow-Ups**<br><br>It's crucial to work closely with your healthcare team to develop a comprehensive treatment plan tailored to your specific condition. Regular follow-ups and monitoring are essential to adjust treatments as needed.

	If you have any more specific questions or need further information, feel free to ask.
I've just been diagnosed with acute stroke. Even though it's bad, I think that with the right treatment I will be able to get better. Do you have any suggestions on how this can be done?	I'm really sorry to hear about your diagnosis. It's good to hear that you're optimistic about recovery, and there are indeed many ways to support your rehabilitation. Here are some suggestions:<br><br>**Medical Treatment**<br><br>1. **Medication:** Your doctor will likely prescribe medications to manage symptoms and prevent further strokes. Common medications include blood thinners, cholesterol-lowering drugs, and blood pressure medications.<br>2. **Surgery:** In some cases, procedures like carotid endarterectomy or angioplasty might be necessary to remove blockages or widen arteries.<br><br>**Rehabilitation Therapies**<br><br>1. **Physical Therapy:** Helps to regain movement and coordination. Exercises can improve strength, flexibility, and balance.<br>2. **Occupational Therapy:** Focuses on improving daily living skills such as dressing, eating, and bathing.<br>3. **Speech Therapy:** If the stroke has affected your ability to speak or swallow, a speech therapist can help you regain these abilities.<br><br>**Lifestyle Changes**<br><br>1. **Diet:** Eat a healthy diet rich in fruits, vegetables, whole grains, and lean proteins. Avoid high salt and high-fat foods.<br>2. **Exercise:** Regular physical activity can improve cardiovascular health and overall well-being. Follow your therapist's guidelines on safe exercises.<br>3. **Quit Smoking:** Smoking significantly increases the risk of stroke. If you smoke, seek help to quit.

	4. **Limit Alcohol:** Excessive drinking can raise your blood pressure and risk of stroke. Limit your intake to moderate levels.<br><br>**Emotional and Psychological Support**<br><br>1. **Counseling:** A therapist can help you cope with the emotional impact of a stroke.<br>2. **Support Groups:** Joining a support group can provide encouragement and understanding from others who are experiencing similar challenges.<br><br>**Monitoring and Follow-up**<br><br>1. **Regular Check-ups:** Keep all follow-up appointments with your healthcare providers to monitor your progress and adjust treatments as needed.<br>2. **Home Monitoring:** Monitor your blood pressure and other vital signs as recommended by your doctor.<br><br>**Education and Awareness**<br><br>1. **Learn About Stroke:** Understanding your condition can help you manage it better. Resources from organizations like the American Stroke Association can be very helpful.<br>2. **Family Involvement:** Educate your family about stroke so they can support you effectively.<br><br>**Stay Positive and Patient**<br><br>Recovery from a stroke can take time. Celebrate small victories and stay positive. Your healthcare team is there to support you every step of the way.<br><br>Would you like more detailed information on any of these suggestions?

The doctor just prescribed some medication for stroke. However, I am really worried about the potential side effects. I don’t want to end up worse than I already am. How long do I need to keep taking them?	It's important to follow your doctor's instructions regarding medication after a stroke, as they are tailored to help your recovery and prevent further strokes. However, concerns about side effects are valid and should be discussed with your healthcare provider. They can provide detailed information about what to expect and how to manage any side effects that may occur.<br><br>The duration for taking stroke medication can vary based on the type of stroke, the specific medications prescribed, and your overall health condition. Some medications may be prescribed for a short period, while others might be necessary for long-term use.<br><br>Common types of medication prescribed after a stroke include:<br><br>1. **Antiplatelet Drugs (e.g., aspirin, clopidogrel)**:<br>○ **Purpose**: To prevent blood clots.<br>○ **Duration**: Often taken long-term.<br>2. **Anticoagulants (e.g., warfarin, dabigatran)**:<br>○ **Purpose**: To prevent new clots from forming.<br>○ **Duration**: Typically taken long-term.<br>3. **Blood Pressure Medications**:<br>○ **Purpose**: To manage hypertension.<br>○ **Duration**: Usually taken long-term.<br>4. **Cholesterol-Lowering Drugs (e.g., statins)**:<br>○ **Purpose**: To reduce the risk of further strokes.<br>○ **Duration**: Often taken for life.<br>5. **Medications for Managing Diabetes**:<br>○ **Purpose**: If you have diabetes, to control blood sugar levels.<br>○ **Duration**: As long as needed to manage diabetes effectively.

	**Steps to Take**<br><br>1. **Discuss Side Effects with Your Doctor**:<br>◦ Bring up your concerns about side effects and ask what you should watch for. Your doctor can adjust your treatment plan if side effects become problematic.<br>2. **Medication Review**:<br>◦ Periodically review your medications with your doctor to ensure they are still necessary and effective.<br>3. **Lifestyle Changes**:<br>◦ Implementing lifestyle changes, such as a healthy diet, regular exercise, and quitting smoking, can sometimes reduce the need for certain medications.<br>4. **Follow-Up Appointments**:<br>◦ Regular follow-ups with your healthcare provider are crucial to monitor your progress and make any necessary adjustments to your treatment plan.<br><br>If you have specific details about the medication you've been prescribed, I can provide more detailed information about potential side effects and management strategies.
I’ve just been diagnosed with a severe stroke in the brain. From the CT scan, there seem to be blood clots everywhere. Is it really possible for	I'm really sorry to hear about your diagnosis. Recovery from a stroke, especially a severe one with widespread blood clots, can be challenging and varies significantly from person to person. Here are some general points about stroke recovery:<br><br>**1. Initial Treatment**<br><br>**Immediate medical treatment** is crucial. Treatments like clot-dissolving medications (e.g., tPA) or procedures like mechanical thrombectomy can sometimes help restore blood flow to affected areas.

<table>
<tr>
<td>the damaged area to recover?</td>
<td>

2. Neuroplasticity

The brain has a remarkable ability to adapt and reorganize itself, known as neuroplasticity. Over time, healthy parts of the brain can sometimes take over functions lost due to stroke.

3. Rehabilitation

Intensive rehabilitation is essential and may include:

Physical Therapy: To improve strength, coordination, and mobility.
Occupational Therapy: To help regain the ability to perform daily activities.
Speech Therapy: To address communication and swallowing difficulties.
Cognitive Therapy: To improve memory, attention, and problem-solving skills.

4. Medications

Medications to manage risk factors and prevent further clots include:

Blood Thinners: To prevent new clots from forming.
Blood Pressure Medications: To control high blood pressure.
Cholesterol-Lowering Drugs: To reduce the risk of further strokes.
Diabetes Medications: To manage blood sugar levels.

5. Lifestyle Changes

Adopting a healthy lifestyle can support recovery and prevent further strokes:

Diet: Eat a balanced diet rich in fruits, vegetables, whole grains, and lean proteins.

</td>
</tr>
</table>

	● **Exercise**: Engage in regular physical activity.<br>● **Avoid Smoking**: Smoking significantly increases stroke risk.<br>● **Limit Alcohol**: Reduce alcohol intake to moderate levels.<br><br>**6. Support Systems**<br><br>**Emotional and psychological support** from family, friends, and support groups is vital. Mental health care, including counseling or therapy, can also be beneficial.<br><br>**General Points to Consider**<br><br>● **Recovery Process**: Recovery can be a long process and may not result in complete restoration of all functions, but many people see significant improvements with time and effort.<br>● **Personalized Plan**: Working closely with your healthcare team to create a personalized recovery plan is crucial.<br>● **Patience and Persistence**: Progress may be slow, and it's important to stay patient and persistent with rehabilitation efforts.<br><br>By following these steps and working with your healthcare team, you can optimize your chances for recovery and improve your quality of life. If you have any specific questions or need more detailed information on any aspect of recovery, please let me know.
I am about to undergo surgery for stroke. This is my first time in the operating room and I am really scared.	It's completely understandable to feel scared before your surgery. Knowing what to expect can help ease some of your anxiety. Here's a general overview of what happens during stroke surgery:<br><br>**Pre-Operation**<br><br>1. **Preparation:**

What will happen to me in there?	○ **Preoperative Assessment:** You will undergo a thorough assessment, including blood tests, imaging scans (like MRI or CT), and a review of your medical history.<br>○ **Consent:** The surgical team will explain the procedure, risks, and benefits. You will need to sign a consent form.<br>○ **Fasting:** You may be asked not to eat or drink for a certain period before the surgery.<br>2. **Admission to Hospital:**<br>○ **Arrival:** You'll arrive at the hospital and be taken to a preoperative area where you will change into a hospital gown.<br>○ **IV Line:** An intravenous (IV) line will be inserted into your arm to administer fluids and medications.<br>3. **Meeting the Team:**<br>○ **Surgeon and Anesthesiologist:** You will meet your surgeon and anesthesiologist, who will explain their roles and what to expect.<br><br>**During the Surgery**<br><br>1. **Anesthesia:**<br>○ **General Anesthesia:** You'll be given medication through the IV line to make you sleep and ensure you don't feel any pain during the procedure.<br>2. **Monitoring:**<br>○ **Vital Signs:** Your heart rate, blood pressure, oxygen levels, and other vital signs will be continuously monitored.<br>3. **The Surgery:**<br>○ **Procedure:** The type of surgery will depend on your specific condition. It could be a clot removal, aneurysm repair, or other procedures to address the stroke's cause.<br>○ **Duration:** The length of the surgery varies, but your surgeon can give you an estimate beforehand.<br><br>**Post-Operation**<br><br>1. **Recovery Room:**

	○ **Waking Up:** You'll wake up in a recovery room where nurses will monitor your vital signs and ensure you're comfortable.<br>○ **Pain Management:** You may receive pain medication if needed.<br>2. **Observation:**<br>○ **Monitoring:** You'll be closely observed for any immediate postoperative complications.<br>○ **Instructions:** The medical team will provide specific instructions for your recovery, including medications and activity restrictions.<br>3. **ICU or Regular Room:**<br>○ Depending on the surgery and your condition, you may be transferred to an intensive care unit (ICU) or a regular hospital room.<br><br>**Tips for Coping**<br><br>● **Ask Questions:** Don't hesitate to ask your medical team any questions you have.<br>● **Stay Informed:** Knowing what to expect can help reduce anxiety.<br>● **Relaxation Techniques:** Practice deep breathing, meditation, or other relaxation techniques to calm your nerves.<br><br>Your medical team is there to support you and ensure the best possible outcome. Take comfort in knowing you are in good hands.

-Recovery

TOT	Responses
I am worried that I will never recover from the stroke I had	I'm sorry to hear about your concerns. Recovery from a stroke can indeed be challenging, but many people do make significant improvements with the right treatments and therapies. Here are some steps and treatments that can be helpful:

a few days ago. I am willing to try anything that may work, including rehabilitation and hyperbaric oxygen therapy.	**1. Rehabilitation Therapy**<br><br>**Physical Therapy (PT):**<br><br>● Focuses on improving strength, coordination, and balance.<br>● Exercises are designed to help regain movement and function.<br><br>**Occupational Therapy (OT):**<br><br>● Helps you relearn daily activities and skills, such as dressing, bathing, and cooking.<br><br>**Speech and Language Therapy:**<br><br>● Assists with communication skills if the stroke has affected your speech or understanding of language.<br><br>**2. Hyperbaric Oxygen Therapy (HBOT)**<br><br>● **HBOT involves breathing pure oxygen in a pressurized room or chamber. Some studies suggest it may help with brain repair and recovery by increasing oxygen supply to damaged tissues.<br>● **Consult with your doctor to see if HBOT is a suitable option for your condition and location.<br><br>**3. Medication**<br><br>● **Medications to reduce the risk of another stroke, manage blood pressure, cholesterol, and diabetes if present.<br>● **Medications to address specific symptoms or complications from the stroke.<br><br>**4. Lifestyle Changes**

	**Healthy Diet:**<br><br>● Focus on a balanced diet rich in fruits, vegetables, lean proteins, and whole grains.<br><br>**Regular Exercise:**<br><br>● Tailored exercises to improve cardiovascular health and aid in recovery.<br><br>**Avoid Smoking and Limit Alcohol:**<br><br>● Both can increase the risk of another stroke and impede recovery.<br><br>**5. Support Networks**<br><br>**Support Groups:**<br><br>● Connecting with others who have had similar experiences can provide emotional support and practical advice.<br><br>**Counseling or Therapy:**<br><br>● Professional support to help with the emotional and psychological aspects of recovery.<br><br>**6. Alternative Therapies**<br><br>**Acupuncture:**<br><br>● Some find it helpful in managing pain and improving overall well-being.

Massage Therapy:

- Can help with muscle tension and stress relief.

7. Technological Aids

Assistive Devices:

- Tools and technologies designed to help with mobility, communication, and daily tasks.

8. Monitoring and Follow-up

Regular follow-up appointments with your healthcare team:

- To monitor progress and make adjustments to your treatment plan as needed.

9. Mental and Emotional Health

Mindfulness and Meditation:

- Techniques to reduce stress and improve mental clarity.

Positive Attitude:

- Maintaining a hopeful and positive outlook can significantly impact your recovery.

Summary

Recovery from a stroke requires a multi-faceted approach that includes medical treatment, rehabilitation, lifestyle changes,

	emotional support, and regular monitoring. Working closely with your healthcare team to develop a comprehensive and personalized recovery plan is crucial for the best possible outcomes. If you have any specific questions or need more detailed information about any of these therapies, feel free to ask.
I am worried that I will never recover from the stroke I had a few days ago. I am willing to try anything that may work, including rehabilitation and intravenous laser irradiation of blood.	I'm really sorry to hear that you're feeling this way. It's completely natural to feel concerned about your recovery, but there are many steps you can take to improve your chances of a full recovery. Here are some key points and steps you might consider:<br><br>**Rehabilitation**<br><br>1. **Physical Therapy**: A physical therapist can help you regain movement and strength through targeted exercises. This is crucial for improving mobility and reducing disability.<br>2. **Occupational Therapy**: This focuses on helping you regain independence in daily activities, such as dressing, bathing, and cooking.<br>3. **Speech Therapy**: If your stroke affected your ability to speak or understand language, a speech therapist can help you improve these skills.<br>4. **Cognitive Rehabilitation**: This helps with memory, problem-solving, and thinking skills, which may be affected after a stroke.<br><br>**Medical Treatments**<br><br>1. **Medication**: Follow your doctor’s recommendations for medications that can help prevent another stroke or address underlying conditions such as high blood pressure, diabetes, or high cholesterol.<br>2. **Intravenous Laser Irradiation of Blood (ILIB)**: This is a less conventional treatment where low-level laser therapy is applied to the blood. Some studies suggest it may have benefits, but it's essential to discuss this with your healthcare provider to understand the potential risks and benefits.

Lifestyle Changes

1. **Healthy Diet**: Eating a balanced diet can help with your overall health and recovery. Focus on fruits, vegetables, whole grains, lean proteins, and healthy fats.
2. **Regular Exercise**: Even gentle exercise can help improve your strength and cardiovascular health. Always consult with your healthcare provider before starting a new exercise regimen.
3. **Quit Smoking and Limit Alcohol**: These can significantly impact your recovery and overall health.
4. **Manage Stress**: Practices like mindfulness, meditation, or counseling can help you cope with the emotional aspects of recovery.

Support Systems

1. **Family and Friends**: Lean on your loved ones for support. They can provide emotional encouragement and practical help.
2. **Support Groups**: Connecting with others who have experienced a stroke can provide comfort and practical advice.
3. **Mental Health Support**: A counselor or psychologist can help you deal with anxiety, depression, or other emotional challenges during your recovery.

Monitoring Progress

1. **Regular Check-ups**: Keep all appointments with your healthcare providers to monitor your progress and adjust treatments as needed.
2. **Track Improvements**: Keeping a journal of your progress can help you stay motivated and provide useful information to your healthcare team.

It's important to stay positive and patient with yourself during this recovery period. Every stroke and recovery journey is unique, so what works for one person might be different for another. Discuss all potential treatments and therapies with your healthcare

	provider to create a plan tailored to your needs.
I am worried that I will never recover from the stroke I had a few days ago. I am willing to try anything that may work, including rehabilitation and transcranial magnetic stimulation.	I'm sorry to hear about your stroke, but it's good that you're proactive about your recovery. It's important to approach this with a comprehensive plan, including medical guidance and rehabilitation. Here are some key points and options to consider:<br><br>**Medical and Rehabilitation Support**<br><br>1. **Consult Your Healthcare Team**:<br>○ **Neurologist**: They can provide detailed information about your specific condition and prognosis.<br>○ **Physiatrist**: A specialist in physical medicine and rehabilitation who can oversee your recovery plan.<br>2. **Rehabilitation Programs**:<br>○ **Physical Therapy (PT)**: To help regain strength and mobility.<br>○ **Occupational Therapy (OT)**: To improve daily functioning and independence.<br>○ **Speech Therapy**: If you have any speech or swallowing difficulties.<br>3. **Lifestyle Changes**:<br>○ **Healthy Diet**: To support brain health and overall recovery.<br>○ **Regular Exercise**: Under guidance, to improve physical and mental well-being.<br>○ **Avoid Smoking and Limit Alcohol**: These can impede recovery.<br><br>**Emerging and Additional Therapies**<br><br>1. **Transcranial Magnetic Stimulation (TMS)**:<br>○ **What It Is**: TMS is a non-invasive procedure that uses magnetic fields to stimulate nerve cells in the brain.<br>○ **Potential Benefits**: It has shown promise in improving motor function and mood in stroke patients, though more research is needed.

	◦ **Considerations**: Discuss with your neurologist to see if you're a suitable candidate. It's usually part of a broader rehabilitation program.<br>2. **Mirror Therapy**:<br>◦ **What It Is**: Uses the reflection of the unaffected limb to create visual feedback that the affected limb is moving.<br>◦ **Potential Benefits**: Can be helpful in regaining motor function and reducing pain.<br>3. **Virtual Reality (VR) Rehabilitation**:<br>◦ **What It Is**: Utilizes VR technology to simulate real-life activities and provide interactive exercises.<br>◦ **Potential Benefits**: Engaging and motivating, may help improve motor skills and cognitive function.<br>4. **Robotics and Exoskeletons**:<br>◦ **What It Is**: Robotic devices that assist with movement and physical therapy exercises.<br>◦ **Potential Benefits**: Can help with regaining strength and movement in affected limbs.<br><br>**Psychological and Emotional Support**<br><br>1. **Counseling and Support Groups**:<br>◦ **Individual Therapy**: To address feelings of anxiety, depression, or frustration.<br>◦ **Support Groups**: Connecting with others who have had similar experiences can provide emotional support and practical advice.<br>2. **Mindfulness and Stress Management**:<br>◦ **Meditation and Relaxation Techniques**: To reduce stress and improve overall mental health.<br>◦ **Cognitive Behavioral Therapy (CBT)**: Can help manage negative thoughts and promote positive thinking.<br><br>**Monitoring and Adjustments**<br><br>1. **Regular Follow-Ups**:<br>◦ **Track Progress**: Regular assessments by your healthcare team to monitor progress and adjust the rehabilitation

	plan as needed.<br>◦ **Adjust Therapies**: Based on your progress, your therapies and treatments might be modified for better results.<br><br>**Staying Informed**<br><br>1. **Stay Updated**:<br>◦ **Research and Innovations**: Keep informed about new treatments and technologies in stroke recovery.<br>◦ **Educational Resources**: Utilize resources from reputable organizations like the American Stroke Association.<br><br>Remember, recovery from a stroke is often a long process that requires patience and persistence. It's important to set realistic goals and celebrate small victories along the way. Working closely with your healthcare team and exploring a variety of therapies can maximize your chances of a successful recovery.
How useful is rehabilitation is for stroke patients? I don't really think that some simple exercises can really help me recover. Exactly what kind of assistance does it provide?	Rehabilitation for stroke patients is highly beneficial and can significantly aid in recovery. It goes beyond simple exercises, encompassing a range of therapies and support strategies designed to address the diverse needs of stroke survivors. Here's how rehabilitation can assist you:<br><br>**1. Physical Therapy**<br><br>● **Motor Skills**: Helps improve muscle strength, coordination, and flexibility.<br>● **Mobility**: Aims to restore the ability to walk and move independently.<br>● **Balance**: Works on balance to prevent falls and enhance stability.<br><br>**2. Occupational Therapy**<br><br>● **Daily Activities**: Teaches strategies to perform everyday tasks such as dressing, eating, and bathing.

- **Cognitive Skills**: Enhances memory, problem-solving, and planning abilities.
- **Use of Assistive Devices**: Trains you to use tools that can aid in daily living.

3. Speech and Language Therapy

- **Communication**: Helps improve speech, language, and communication skills.
- **Swallowing**: Addresses difficulties in swallowing, ensuring safe eating and drinking.

4. Psychological Support

- **Emotional Well-being**: Provides counseling to deal with depression, anxiety, and emotional changes.
- **Cognitive Rehabilitation**: Targets issues like attention, memory, and executive function.

5. Social Support and Education

- **Family Training**: Educates family members on how to support and care for the stroke survivor.
- **Support Groups**: Offers a community of individuals who share similar experiences, providing emotional and practical support.

6. Technological Aids

- **Robotics and Virtual Reality**: Utilize advanced technology to enhance physical and cognitive rehabilitation.
- **Tele-rehabilitation**: Provides remote therapy options, allowing continued rehabilitation at home.

7. Customized Rehabilitation Plans

- **Individualized Approach**: Tailors therapy to address specific deficits and goals unique to each patient.

	● **Progress Monitoring**: Regularly assesses progress and adjusts the plan as needed to maximize recovery.<br><br>**Evidence of Effectiveness**<br><br>Studies have shown that stroke rehabilitation can:<br><br>● **Improve Functional Outcomes**: Patients undergoing rehabilitation are more likely to regain independence.<br>● **Reduce Disability**: Effective in minimizing long-term disability.<br>● **Enhance Quality of Life**: Contributes to better physical, emotional, and social well-being.<br>● **Promote Neuroplasticity**: Encourages the brain to rewire itself, forming new neural connections.<br><br>Rehabilitation is a crucial component of stroke recovery, significantly impacting the degree of independence and quality of life post-stroke. It's essential to start rehabilitation as soon as possible after a stroke to achieve the best outcomes.
I was recently discharged from the hospital after being treated for stroke. Unfortunately, I need to go back for rehabilitation each week. I have a feeling that it will take some time to recover. Can you tell me how long it will	Rehabilitation for stroke patients is highly beneficial and can significantly aid in recovery. It goes beyond simple exercises, encompassing a range of therapies and support strategies designed to address the diverse needs of stroke survivors. Here's how rehabilitation can assist you:<br><br>**1. Physical Therapy**<br><br>● **Motor Skills**: Helps improve muscle strength, coordination, and flexibility.<br>● **Mobility**: Aims to restore the ability to walk and move independently.<br>● **Balance**: Works on balance to prevent falls and enhance stability.<br><br>**2. Occupational Therapy**<br><br>● **Daily Activities**: Teaches strategies to perform everyday tasks such as dressing, eating, and bathing.

last?	• **Cognitive Skills**: Enhances memory, problem-solving, and planning abilities.<br>• **Use of Assistive Devices**: Trains you to use tools that can aid in daily living.<br><br>**3. Speech and Language Therapy**<br><br>• **Communication**: Helps improve speech, language, and communication skills.<br>• **Swallowing**: Addresses difficulties in swallowing, ensuring safe eating and drinking.<br><br>**4. Psychological Support**<br><br>• **Emotional Well-being**: Provides counseling to deal with depression, anxiety, and emotional changes.<br>• **Cognitive Rehabilitation**: Targets issues like attention, memory, and executive function.<br><br>**5. Social Support and Education**<br><br>• **Family Training**: Educates family members on how to support and care for the stroke survivor.<br>• **Support Groups**: Offers a community of individuals who share similar experiences, providing emotional and practical support.<br><br>**6. Technological Aids**<br><br>• **Robotics and Virtual Reality**: Utilize advanced technology to enhance physical and cognitive rehabilitation.<br>• **Tele-rehabilitation**: Provides remote therapy options, allowing continued rehabilitation at home.<br><br>**7. Customized Rehabilitation Plans**<br><br>• **Individualized Approach**: Tailors therapy to address specific deficits and goals unique to each patient.

	● **Progress Monitoring**: Regularly assesses progress and adjusts the plan as needed to maximize recovery.<br><br>**Evidence of Effectiveness**<br><br>Studies have shown that stroke rehabilitation can:<br><br>● **Improve Functional Outcomes**: Patients undergoing rehabilitation are more likely to regain independence.<br>● **Reduce Disability**: Effective in minimizing long-term disability.<br>● **Enhance Quality of Life**: Contributes to better physical, emotional, and social well-being.<br>● **Promote Neuroplasticity**: Encourages the brain to rewire itself, forming new neural connections.<br><br>Rehabilitation is a crucial component of stroke recovery, significantly impacting the degree of independence and quality of life post-stroke. It's essential to start rehabilitation as soon as possible after a stroke to achieve the best outcomes.

Claude responses:

-Prevention

TOT	Responses
My dad recently suffered a stroke. Now I am really worried about myself too. What if I	I'm sorry to hear about your dad's stroke. That must be really scary and stressful for you and your family. It's understandable to be worried about your own risk after seeing a loved one go through that.<br><br>While strokes can happen at any age, there are several key things you can do to reduce your risk:

suddenly get a stroke and die? I need to know how to prevent this from happening.	1. Manage blood pressure - High blood pressure is a major stroke risk factor. Have your blood pressure checked regularly and work with your doctor to keep it in a healthy range through lifestyle changes or medication if needed.<br><br>2. Don't smoke and avoid secondhand smoke - Smoking greatly increases stroke risk.<br><br>3. Exercise regularly - Getting moderate exercise most days helps reduce stroke risk factors like obesity, high blood pressure, and high cholesterol.<br><br>4. Eat a healthy diet low in salt, saturated fats, and cholesterol - Focus on fruits, vegetables, whole grains, lean protein, and healthy fats.<br><br>5. Limit alcohol - Heavy or binge drinking can raise blood pressure.<br><br>6. Control diabetes - Having diabetes increases stroke risk, so carefully managing it is important.<br><br>7. Be aware of atrial fibrillation - This irregular heartbeat condition increases stroke risk, so getting it treated is advisable.<br><br>The good news is that up to 80% of strokes may be prevented by addressing risk factors through lifestyle changes like those above. Talking to your doctor about your specific risk factors and taking preventive steps can provide reassurance. Don't hesitate to ask for help coping with the stress too.
My parents recently suffered a stroke. I've recently found an article on the	I'm sorry to hear about your parents suffering strokes. That must be a very difficult situation for your family. Regarding your question about the potential hereditary nature of strokes:<br><br>There are some genetic factors that can increase a person's risk of having a stroke, however, having a parent who had a stroke

internet saying that it is hereditary. Does it mean that I will get one too?	does not necessarily mean you will definitely have one yourself. Here are a few key points about the hereditary aspects of stroke risk:<br><br>- Stroke does tend to run in families to some degree, likely due to shared genetic and environmental factors.<br><br>- However, lifestyle factors like diet, exercise, smoking, and managing conditions like high blood pressure play a huge role as well.<br><br>- Certain genetic disorders that affect blood vessels or clotting factors can significantly raise stroke risk if inherited.<br><br>- Your overall stroke risk is influenced by a combination of your genes, family history, age, gender, race/ethnicity, and lifestyle choices.<br><br>The good news is that even if you have a family history of stroke, there are many preventative measures you can take like not smoking, exercising regularly, maintaining a healthy diet, and managing conditions like high blood pressure and diabetes.<br><br>While having a parent with stroke does increase your risk somewhat, it does not mean a stroke is inevitable for you. Being proactive about your health can greatly reduce your personal stroke risk. I'd suggest discussing your specific family history and risk factors with your doctor.
My dad recently suffered a stroke. Perhaps it’s because he often stays up late for work. I seem to have the same	I'm sorry to hear about your father's stroke. Strokes can be very serious health events, and it's understandable that you want to take steps to reduce your own risk. Here are some lifestyle changes that may help prevent strokes:<br><br>1. Manage stress levels: Chronic stress can increase blood pressure and inflammatory factors that contribute to stroke risk. Practice stress management techniques like meditation, yoga, deep breathing exercises, or talking to a therapist.

problem too. I wonder how I can change my lifestyle to prevent this.	2. Get enough sleep: As you mentioned, lack of sleep has been linked to increased stroke risk. Aim for 7-9 hours of quality sleep per night by establishing a consistent sleep schedule and creating a relaxing bedtime routine.<br><br>3. Exercise regularly: Physical activity helps control weight, reduce blood pressure, improve cholesterol levels, and boost overall cardiovascular health. Aim for at least 150 minutes of moderate exercise or 75 minutes of vigorous exercise per week.<br><br>4. Follow a healthy diet: Eat plenty of fruits, vegetables, whole grains, lean proteins, and healthy fats. Limit sodium, saturated fats, and added sugars. The DASH or Mediterranean diets are great options for stroke prevention.<br><br>5. Stop smoking: Smoking doubles the risk of ischemic stroke. If you smoke, make a plan to quit with support from your doctor or a cessation program.<br><br>6. Limit alcohol: Heavy, long-term alcohol use can increase stroke risk. If you drink alcohol, do so only in moderation (no more than 1 drink per day for women, 2 for men).<br><br>7. Manage medical conditions: Keep conditions like high blood pressure, high cholesterol, diabetes, and obesity well-controlled through lifestyle changes and medication if needed.<br><br>Making some of these adjustments can seem challenging, but small steps can lead to big improvements in your overall health and stroke risk reduction. Be sure to discuss your concerns with your doctor as well.
My dad recently suffered a stroke. I think it’s because he seldom exercises.	I'm sorry to hear about your dad's stroke. Strokes can be very serious, and it's good that you want to take steps to reduce your own risk. Here are some exercises that may help decrease the risk of stroke:<br><br>1. Aerobic exercise: Activities that get your heart rate up like brisk walking, jogging, swimming, cycling, or dancing for at least 150

What kinds of exercise can I do to decrease the risk of stroke?	minutes per week can improve cardiovascular health and lower stroke risk.<br><br>2. Strength training: Lifting weights or doing resistance exercises 2-3 times per week can help keep muscles strong and improve overall fitness.<br><br>3. Yoga or tai chi: These mind-body practices that combine gentle movements with breathing techniques may help reduce stress and lower blood pressure.<br><br>4. Adding more general movement: Even light activity like gardening, housework, or taking regular breaks from sitting can provide benefits when combined with more vigorous exercise.<br><br>The key is to incorporate aerobic exercise most days of the week and include some strengthening activities too. Staying active helps control weight, cholesterol levels, blood pressure - all factors that impact stroke risk. Be sure to check with your doctor before significantly increasing exercise, especially if you have any existing health conditions. Making lifestyle changes can make a real difference in stroke prevention.
My dad recently suffered a stroke. I have a feeling it’s because he enjoys eating fast foods. How can I reduce the risk of stroke by changing my diet?	I'm sorry to hear about your dad's stroke. Strokes can be very serious, and dietary factors do play a role in stroke risk. Here are some dietary tips that may help reduce your stroke risk:<br><br>1. Eat more fruits and vegetables: Diets rich in fruits and vegetables provide nutrients, antioxidants, and fiber that can help lower blood pressure and cholesterol levels, reducing stroke risk.<br><br>2. Choose whole grains: Opt for whole-grain breads, cereals, pasta, and rice instead of refined grains. Whole grains are higher in fiber and nutrients that may help regulate blood pressure.

	3. Limit sodium intake: Excess sodium can raise blood pressure, a major risk factor for stroke. Avoid processed and fast foods, which are typically high in sodium, and don't add extra salt to your meals.<br><br>4. Increase potassium intake: Potassium can help counter the effects of sodium and lower blood pressure. Good sources include bananas, sweet potatoes, spinach, and low-fat dairy products.<br><br>5. Eat more fish: Fatty fish like salmon, mackerel, and sardines are rich in omega-3 fatty acids, which can help reduce inflammation and lower stroke risk.<br><br>6. Limit saturated and trans fats: These unhealthy fats can raise cholesterol levels and increase stroke risk. Avoid fried foods, full-fat dairy products, and processed snacks.<br><br>7. Maintain a healthy weight: Being overweight or obese increases stroke risk. A diet rich in fruits, vegetables, lean proteins, and whole grains can help with weight management.<br><br>Remember, a balanced diet along with regular exercise and not smoking can significantly reduce your risk of stroke and other cardiovascular diseases.

-Diagnosis

TOT	Responses
I’ve been having headaches quite often these days, especially on cold	I do not have enough information to determine if your headaches could be a sign of stroke. However, frequent or worsening headaches, especially those that seem to be affected by cold weather, are concerning and warrant medical evaluation. Here are a few points to consider:

days. They seem to be getting worse with time. I'm really worried about my condition. Could it be a sign of stroke?	- Stroke can sometimes present with severe, sudden headache, but usually has other neurological symptoms like numbness, slurred speech, vision changes, weakness on one side of the body.<br><br>- Your headaches being recurrent and linked to cold weather may point to an underlying condition like migraines, sinus issues, or sometimes more serious problems like brain aneurysms.<br><br>- Worsening headache patterns over time should be taken seriously, as this can indicate a progressive neurological disorder that needs diagnosis and treatment.<br><br>My advice would be to make an appointment with your primary care doctor or a neurologist as soon as possible. They can take a detailed history, examine you, and potentially order imaging tests like a CT scan or MRI to investigate the cause.<br><br>Try to provide the doctor with details like frequency, location, severity and timing of the headaches, triggers like cold weather, associated symptoms, and any medications you've tried. Early evaluation is important for proper diagnosis and to rule out any serious underlying conditions. Don't ignore worsening headaches - get them evaluated promptly.
I woke up this morning with my hand feeling really numb. I also think that my neck is sort of stiff too. Could these be the symptoms of a stroke?	The symptoms you describe - numbness or tingling in the hand along with neck stiffness - could potentially be signs of a stroke, but they can also have other less serious causes. Here are a few key points:<br><br>- Sudden numbness or weakness, especially on one side of the body, is one of the major warning signs of a possible stroke. This numbness or weakness often affects the face, arm and leg on one side.<br><br>- Neck pain or stiffness alone is usually not a stroke symptom, but could point to a musculoskeletal issue like a pinched nerve.<br><br>- However, the combination of one-sided numbness/weakness along with neck symptoms could potentially indicate a stroke

	affecting the blood supply to part of the brain stem.<br><br>- Other stroke symptoms to watch for include sudden confusion, trouble speaking or understanding speech, dizziness, loss of balance/coordination, and severe headache.<br><br>If the numbness/weakness persists or you develop any other neurological symptoms, it's advisable to get evaluated by a doctor immediately, as quick treatment is crucial for stroke. However, if the numbness is mild and transient, and the neck stiffness feels more like typical muscle tension, it may not necessarily be a stroke. Erring on the safe side is wise when it comes to stroke-like symptoms.
I’ve been feeling dizzy for several weeks already. To make matters worse, the headaches seem to be getting worse with time. Do you think I have a stroke?	Based on the limited information provided, persistent dizziness and worsening headaches over several weeks are concerning symptoms that warrant medical evaluation, but do not necessarily indicate you have had a stroke. Here are a few points to consider:<br><br>- Stroke symptoms tend to come on suddenly, not gradually over weeks. Classic stroke symptoms include one-sided weakness/numbness, speech difficulties, vision problems, severe headache.<br><br>- Persistent dizziness and headaches can have many possible causes like migraines, inner ear disorders, low blood pressure, dehydration, side effects of medications etc.<br><br>- However, new, severe or worsening headaches coupled with dizziness should be evaluated promptly by a doctor to rule out any serious underlying condition.<br><br>- Certain risk factors like high blood pressure, diabetes, high cholesterol increase the risk of stroke if left uncontrolled.

	My advice would be to not try to self-diagnose, but make an appointment with your primary care physician or visit an urgent care facility to get properly evaluated. They can take a history, examine you, order any necessary tests and provide the correct diagnosis and treatment plan. Delaying medical care for persistent, worsening neurological symptoms carries risks. It's better to get it checked out thoroughly.
My blood pressure has been higher than usual these days. So high that my head hurts even when I walk around. Could it be a stroke?	A sustained high blood pressure with headaches could potentially be a warning sign of a more serious condition like an impending stroke. However, there could also be other causes. Here are a few points to consider:<br><br>- Very high blood pressure readings (e.g. over 180/120 mmHg) along with a severe, sudden headache, vision changes, numbness, weakness on one side of the body could indicate a hypertensive crisis needing emergency care for risk of stroke.<br><br>- Moderately elevated blood pressure with headaches may be caused by conditions like anxiety, stress, medication side effects, etc. and may not necessarily mean an imminent stroke is happening.<br><br>- Recurrent headaches with high readings over time do increase the risk of complications like stroke down the line if the high BP is not treated properly.<br><br>My advice would be:<br><br>1) Monitor your BP readings carefully and note any other symptoms like numbness, vision changes, etc.<br><br>2) See your doctor promptly, especially if readings remain very high (over 180/120). Get evaluated for any underlying causes.<br><br>3) Follow your doctor's guidance on medications, lifestyle changes to control your BP and reduce your stroke risk.

	4) If you develop a sudden, severe headache unlike usual, vision changes, weakness - seek emergency care immediately as it could indicate a stroke.<br><br>Properly managed, high BP alone does not always lead to a stroke. But getting evaluated and treated is crucial to reduce your risk. Don't wait if symptoms worsen.
Ever since my dad had a stroke, I've been constantly on the lookout for the signs. Can you tell me exactly what the symptoms are? Perhaps this will make me feel more at ease	Sure, being aware of the signs and symptoms of a stroke is important so you can act quickly if one occurs. Here are the main signs and symptoms to watch out for:<br><br>- Sudden numbness or weakness in the face, arm or leg, especially on one side of the body<br>- Sudden confusion, trouble speaking or understanding speech<br>- Sudden trouble seeing in one or both eyes<br>- Sudden trouble walking, dizziness, loss of balance or lack of coordination<br>- Sudden severe headache with no known cause<br><br>The acronym F.A.S.T. can help recognize the main symptoms:<br>F - Face drooping<br>A - Arm weakness<br>S - Speech difficulty<br>T - Time to call emergency services<br><br>If you notice one or more of these signs, even if the symptoms go away, it's crucial to call 911 or get to a hospital immediately. Quick treatment can reduce brain damage and potentially save a life.<br><br>Let me know if you need any clarification or have additional questions! Being vigilant about recognizing stroke symptoms can help

	ensure rapid medical care if needed.

-Treatment

TOT	Responses
I've just been diagnosed with stroke. The doctor told me that there is severe blood plaque blockage. I think that this is way more serious than I thought. Are there any medications or treatments that can help?	I'm sorry to hear about your stroke diagnosis and the news of severe blood plaque blockage. That must be very concerning. Strokes are indeed serious medical events that require prompt treatment.<br><br>There are several medications and treatments that may help, depending on the type of stroke, location of the blockage, and time since symptom onset. Some potential options include:<br><br>Clot-Busting Medications (e.g. tPA)<br>- These can help dissolve a blood clot if given within a few hours of an ischemic stroke caused by blockage.<br><br>Catheter Procedures<br>- Doctors may perform a catheter procedure to physically remove or break up the clot blocking blood flow.<br><br>Blood Thinners<br>- Antiplatelet drugs like aspirin or anticoagulants may be prescribed long-term to prevent future clots.<br><br>Surgery<br>- In some cases, surgery like carotid endarterectomy may be needed to reopen a narrowed artery.<br><br>Rehabilitation<br>- Physical, occupational, and speech therapy can help recover abilities affected by the stroke.

	The most appropriate course of action depends on many factors. I would recommend having an open discussion with your stroke team about all the options and getting a clear plan for your treatment and recovery. The sooner blockages are addressed, the better for preventing further brain damage. Please don't hesitate to advocate for yourself throughout this process.
I've just been diagnosed with acute stroke. Even though it's bad, I think that with the right treatment I will be able to get better. Do you have any suggestions on how this can be done?	I'm so sorry to hear you've been diagnosed with an acute stroke. That must be extremely difficult and scary. You're right that with prompt treatment, many people are able to recover substantially from strokes. Here are some suggestions that may help in your recovery:<br><br>• Seek treatment immediately if you haven't already. Getting clot-busting medication like tPA within 3-4.5 hours of stroke onset can help minimize brain damage. Endovascular thrombectomy up to 24 hours can remove larger clots.<br><br>• Once stable, you'll likely need rehabilitation to regain functions affected by the stroke. This usually includes physical, occupational, and speech therapy to retrain the brain and rebuild skills.<br><br>• Be guided by your doctors, but rehab generally starts very soon after the stroke, even within a day or two. Earlier and more intensive rehab is associated with better outcomes.<br><br>• Stick with the rehab program diligently. It can be frustrating, but putting in the work maximizes your potential for regaining mobility, speech, cognition, and independence.<br><br>• Make lifestyle changes recommended by your doctors - stop smoking, treat conditions like high blood pressure/diabetes, adopt a stroke prevention diet, exercise as able.<br><br>• Stay positive and give yourself time. Recovery is an ongoing process but the brain can be remarkably plastic, especially with

	rehabilitation.<br><br>The most important things are following your treatment team's guidance while staying committed and hopeful about regaining your capabilities. Let me know if any other specific stroke recovery questions come up.
The doctor just prescribed some medication for stroke. However, I am really worried about the potential side effects. I don’t want to end up worse than I already am. How long do I need to keep taking them?	I don't actually have enough specifics about your medical condition or the medication prescribed to advise on how long you need to take it. Medication regimens can vary greatly depending on the drug, dosage, your individual health status and risks, and the goal of treatment.<br><br>However, I understand your concerns about potential side effects. It's wise to be cautious about new medications. The best thing to do is have an open discussion with your doctor about:<br><br>- Exactly what condition the medication is meant to treat and how it works<br>- What the expected benefits are and how long until you may see improvement<br>- What the potential side effects are, their likelihood, and how to monitor for them<br>- How long the typical course of treatment lasts for your condition<br>- If there are any alternative treatments to consider<br><br>Your doctor can explain the reasoning behind prescribing this particular medication for you and address your specific concerns. Don't hesitate to ask questions until you fully understand the risks and benefits involved. Following dosage instructions carefully is also important for managing side effects.<br><br>Ultimately, you and your doctor will need to weigh the medication's effects against your stroke recovery needs. Don't stop taking it before consulting your doctor, as that could have negative impacts as well. An open physician-patient dialogue is key.

I've just been diagnosed with a severe stroke in the brain. From the CT scan, there seem to be blood clots everywhere. Is it really possible for the damaged area to recover?	I'm sorry to hear about your stroke diagnosis. Strokes can indeed be very serious medical events. However, the brain does have an remarkable ability to recover and reorganize functions through neuroplasticity, even after significant damage.<br><br>Some key points about stroke recovery:<br><br>- The location and extent of the stroke play a big role in what functions may be impaired and the potential for recovery. Strokes affecting the "non-dominant" hemisphere (right side in most people) tend to allow for better language recovery.<br><br>- Time is critical. The sooner blood flow is restored to the damaged areas, the more brain tissue can potentially be saved. Clot-busting drugs like tPA can help if given shortly after stroke onset.<br><br>- Rehabilitation therapies like physical, occupational, and speech therapy are crucial. They help rewire undamaged brain regions to take over lost functions.<br><br>- Recovery often continues over 1-2 years after a stroke as the brain gradually reorganizes itself through neuroplasticity. Improvement can still occur even years later with rehabilitation.<br><br>- Younger patients tend to have better recovery outcomes compared to older adults due to greater neuroplasticity.<br><br>So while a severe stroke obviously causes significant initial deficits, the brain has a remarkable capacity for repair and regeneration over time with proper medical care and rehabilitative assistance. Maintaining hope and diligence with therapies is important. Each recovery is unique, but meaningful improvement is very possible for many stroke survivors.
I am about to undergo surgery for	I understand your anxiety about undergoing surgery - it's completely normal to feel scared, especially when facing something as major as a procedure to treat a stroke. However, try to take some reassurance knowing that you'll be in highly capable hands with

stroke. This is my first time in the operating room and I am really scared. What will happen to me in there?	the surgical team. Here's generally what to expect when you go into the operating room:<br><br>Before the Surgery:<br>- You'll change into a hospital gown and they may insert an IV line into your arm to administer fluids and medications.<br>- The anesthesia team will go over your medical history and explain the process of being put under general anesthesia for the operation.<br><br>During the Surgery:<br>- You'll be wheeled into the OR suite and helped onto the operating table. Equipment will be attached to monitor your vital signs.<br>- The anesthesiologist will start giving you medications through your IV to make you deeply unconscious and pain-free during the procedure.<br>- The surgical team will clean and sterilize the operative area, such as your head for a brain procedure related to the stroke.<br>- The neurosurgeon will make the required incisions and operate, using advanced imaging and tools to safely treat the area affected by the stroke.<br><br>After the Surgery:<br>- You'll be moved to a recovery room while still unconscious from the anesthesia as its effects slowly wear off.<br>- Nurses will closely monitor you as you wake up to ensure you are stable and managing any discomfort appropriately.<br><br>The operating room can seem daunting, but just focus on breathing and try to stay calm knowing you'll essentially be "asleep" for the actual operation part. The doctors and nurses are tremendously skilled at their jobs. Let me know if any other specific concerns are on your mind.

-Recovery

TOT	Responses
I am worried that I will never recover from the stroke I had a few days ago. I am willing to try anything that may work, including rehabilitation and hyperbaric oxygen therapy.	I don't actually have specific details about your medical condition or history. However, I can provide some general information that may be helpful regarding stroke recovery.<br><br>It's understandable to feel worried after having a stroke, as they can have significant impacts. However, with proper treatment and rehabilitation, many stroke survivors are able to regain abilities and recover function over time.<br><br>Some key points about stroke recovery:<br><br>- Rehabilitation plays a crucial role. This includes physical, occupational, and speech therapy tailored to your specific needs and deficits. Consistent rehabilitation can help retrain the brain and body.<br><br>- Recovery is an ongoing process. With time and hard work in rehab, the brain can forge new neural connections allowing some abilities to be regained, even years later.<br><br>- Attitude and determination are important. Staying motivated and putting in the work in rehab can positively impact recovery.<br><br>- New treatments are being researched. Things like constraint-induced therapy, robotic aids, brain stimulation, and hyperbaric oxygen therapy show some promise when combined with rehab.<br><br>The most important things are to follow your doctors' advice, be diligent with rehabilitation, maintain a positive mindset, and not lose hope. Many stroke survivors are able to regain significant function, especially with early and intensive rehab. The road may be difficult, but recovery is very possible with patience and perseverance.

I am worried that I will never recover from the stroke I had a few days ago. I am willing to try anything that may work, including rehabilitation and intravenous laser irradiation of blood.	I'm sorry to hear you had a stroke recently. Recovering from a stroke can be a long and difficult process, but many people are able to regain significant function with time, effort, and appropriate treatment.<br><br>Regarding your treatment options, rehabilitation is definitely recommended as part of the standard care after a stroke. This typically involves working with physical, occupational, and speech therapists to regain skills that were lost or impaired.<br><br>As for intravenous laser irradiation of blood, this is a much less common and experimental treatment. From the medical literature in 2023, there was some very early research looking at using low-level laser therapy delivered intravenously to potentially help neurological recovery after stroke. However, the evidence was still quite preliminary and more study was needed.<br><br>My advice would be to focus first on proven stroke rehabilitation methods recommended by your doctors and therapists. Stick with the standard rehab program diligently as that gives you the best chance at maximizing your recovery. You can certainly discuss experimental treatments like laser therapy with your doctor, but be aware they are unproven and potentially risky. Don't lose hope - many stroke survivors are able to regain significant abilities with time and hard work in rehab. I wish you the very best in your recovery.
I am worried that I will never recover from the stroke I had a few days ago. I am willing to try anything that may work, including rehabilitation and transcranial	I don't actually have enough specific information about your medical condition and stroke to make recommendations. Recovery from a stroke can vary greatly depending on factors like the area of the brain affected, severity, your age, rehabilitation efforts, and more. A few general thoughts:<br><br>- Be patient with yourself and don't lose hope. Recovery can be a long process, but the brain has remarkable abilities to heal and rewire itself over time.<br><br>- Listen closely to your doctors and follow their treatment plan, whether that involves rehab, medications, lifestyle changes, or other approaches proven to aid stroke recovery.

magnetic stimulation.	- Ask your medical team about all options that could help in your situation, including techniques like transcranial magnetic stimulation if they feel it may be beneficial for you.<br><br>- Take an active role in your recovery by doing prescribed exercises/therapies, making healthy lifestyle choices, and having a positive, determined mindset.<br><br>- Don't hesitate to seek support from loved ones, support groups, counseling services if struggling emotionally or mentally during this process.<br><br>The most important thing is to work closely with your doctors, have patience and determination, and never give up hope. With proper treatment and rehabilitation, many stroke survivors can recover remarkably over time. I wish you all the best in your recovery process.
How useful is rehabilitation is for stroke patients? I don’t really think that some simple exercises can really help me recover. Exactly what kind of assistance does it provide?	Rehabilitation can play a crucial role in helping stroke patients regain abilities and function that were impacted by the stroke. While it may seem like simple exercises, rehabilitation is a comprehensive process that aims to address various aspects of recovery. Here's how rehabilitation can assist stroke patients:<br><br>1. Physical therapy: This focuses on improving mobility, strength, balance, and coordination. Therapists use exercises, gait training, and assistive devices to help patients relearn movement patterns and regain independence in activities like walking, transferring, and performing daily tasks.<br><br>2. Occupational therapy: This targets the skills needed for daily living activities, such as dressing, grooming, and cooking. Occupational therapists work on fine motor skills, cognitive abilities, and adaptive techniques to help patients become more independent and confident in their daily routines.

	3. Speech-language therapy: Many stroke patients experience difficulties with speech, language, and swallowing. Speech therapists work on improving communication skills, language comprehension, and swallowing techniques to help patients regain these essential functions.<br><br>4. Cognitive rehabilitation: Strokes can affect cognitive abilities like memory, attention, problem-solving, and decision-making. Cognitive rehabilitation exercises aim to retrain and improve these cognitive functions, which are essential for everyday activities and independence.<br><br>5. Psychological support: Stroke can have emotional and psychological impacts, such as depression, anxiety, and frustration. Rehabilitation teams often include counseling and support to help patients cope with the changes and challenges they face during recovery.<br><br>6. Education and support for caregivers: Rehabilitation also involves educating and training family members or caregivers on how to support the patient's recovery and assist with daily activities at home.<br><br>The specific rehabilitation plan is tailored to each patient's individual needs, abilities, and goals. With time, patience, and consistent effort, rehabilitation can help stroke patients regain significant functional improvements, promote neuroplasticity (rewiring of the brain), and improve their overall quality of life.
I was recently discharged from the hospital after being treated for stroke. Unfortunately, I need	The length of time needed for stroke rehabilitation can vary significantly from person to person, depending on several factors:<br><br>1. Severity of the stroke: Generally, the more severe the stroke, the longer the recovery period. Strokes that cause greater brain damage and more debilitating effects tend to require more extensive rehabilitation.

to go back for rehabilitation each week. I have a feeling that it will take some time to recover. Can you tell me how long it will last?	2. Type of stroke: Ischemic strokes (caused by a blood clot) and hemorrhagic strokes (caused by bleeding in the brain) may have different recovery trajectories and rehabilitation needs.<br><br>3. Age and overall health: Younger patients and those with better overall health before the stroke may recover more quickly than older patients or those with pre-existing medical conditions.<br><br>4. Extent of impairments: The specific abilities affected by the stroke, such as paralysis, speech difficulties, or cognitive impairments, will influence the type and duration of rehabilitation required.<br><br>5. Individual response to therapy: Some individuals may respond better to rehabilitation than others, even with similar stroke severity.<br><br>That said, many stroke survivors require rehabilitation services for several months to a year or more after the stroke, depending on their progress and goals. It's common for intensive inpatient rehabilitation to last a few weeks to a couple of months, followed by outpatient rehabilitation for an extended period.<br><br>The key is to work closely with your rehabilitation team, set realistic goals, and be patient and persistent with the process. With time, consistent effort, and the right support, many stroke survivors can regain significant function and independence.

Gemini responses:

-Prevention

TOT	Responses
My dad recently suffered a stroke. Now I am really worried about myself too. What if I suddenly get a stroke and die? I need to know how to prevent this from happening.	I understand how worried you must be after your dad's stroke. It's completely natural to be concerned about your own health.<br><br>The good news is that there are steps you can take to significantly reduce your risk of stroke.<br><br>Here are some key lifestyle changes that can make a big difference:<br><br>● **Healthy diet:** Focus on fruits, vegetables, whole grains, and lean protein. Limit saturated and trans fats, cholesterol, and salt https://health.gov/myhealthfinder/health-conditions/heart-health/lower-your-risk-stroke.<br>● **Exercise regularly:** Aim for at least 150 minutes of moderate-intensity exercise per week, or 75 minutes of vigorous exercise https://health.gov/myhealthfinder/health-conditions/heart-health/lower-your-risk-stroke.<br>● **Maintain a healthy weight:** Being overweight or obese increases stroke risk.<br>● **Manage blood pressure:** High blood pressure is a major risk factor. Get regular checkups and take medication as prescribed by your doctor.<br>● **Control blood sugar:** If you have diabetes, managing your blood sugar is crucial.<br>● **Limit alcohol:** Excessive alcohol consumption can raise blood pressure.

	• **Don't smoke:** Smoking is a major risk factor for stroke. Quitting smoking is one of the best things you can do for your health.<br><br>If you have any risk factors like high blood pressure or cholesterol, it's important to see your doctor regularly and follow their treatment plan. They can also assess your individual risk and recommend specific steps for you.<br><br>Here are some resources that you might find helpful:<br><br>• American Heart Association https://www.health.harvard.edu/womens-health/8-things-you-can-do-to-prevent-a-stroke<br>• Centers for Disease Control and Prevention https://www.cdc.gov/stroke/about/index.html<br>• NHS (UK National Health Service) https://www.northamptongeneral.nhs.uk/Services/Our-Clinical-Services-and-Departments/Medicine/Stroke-Services/Documents/Stroke-Prevention.pdf
My parents recently suffered a stroke. I've recently found an article on the internet saying that it is hereditary. Does	While having a family history of stroke does increase your risk, it doesn't guarantee you'll have one. There are two main points to consider:<br><br>1. **Hereditary Stroke Conditions:** There are rare genetic conditions that can increase the likelihood of stroke. These are

it mean that I will get one too?	uncommon, but if you're concerned, talk to your doctor about your family history and see if genetic testing might be appropriate.<br>2. **Shared Risk Factors:** Often, families share similar lifestyles and habits. These habits, like diet, exercise, and smoking, can all influence stroke risk. So, a family history might indicate a need to focus on healthy lifestyle choices to reduce your own risk.<br><br>Here's what you can do:<br><br>● **Speak to your doctor:** Let them know about your parents' strokes and any concerns you have. They can assess your individual risk factors and advise you on preventative measures.<br>● **Understand your risk factors:** These include high blood pressure, high cholesterol, diabetes, and smoking. Talk to your doctor about how to manage these if applicable.<br>● **Embrace a healthy lifestyle:** Eat a balanced diet, exercise regularly, and maintain a healthy weight.<br><br>Remember, most strokes are preventable. By taking charge of your health, you can significantly reduce your risk.

My dad recently suffered a stroke. Perhaps it’s because he often stays up late for work. I seem to have the same problem too. I wonder how I can change my lifestyle to prevent this.	I'm sorry to hear about your dad's stroke. It's understandable that you're concerned about your own sleep habits, especially considering the potential link to late nights. Here are some lifestyle changes you can make to improve your sleep quality:<br><br>**Develop a Consistent Sleep Schedule:**<br><br>● Go to bed and wake up at the same time each day, even on weekends. This helps regulate your body's natural sleep-wake cycle (circadian rhythm).<br><br>**Create a Relaxing Bedtime Routine:**<br><br>● Wind down for 30-60 minutes before bed with calming activities like reading, taking a warm bath, or light stretching.<br>● Avoid stimulating activities like watching TV, using electronic devices, or working.<br><br>**Optimize Your Sleep Environment:**<br><br>● Make sure your bedroom is cool, dark, and quiet. Invest in blackout curtains, earplugs, or a white noise machine if necessary.<br>● Reserve your bed for sleep and intimacy only. Avoid working, watching TV, or using electronic devices in bed.

	**Diet and Exercise:**<br>• Avoid caffeine and alcohol close to bedtime, as they can disrupt sleep.<br>• Eat a healthy diet and exercise regularly, but avoid strenuous activity close to bedtime.<br>**Light Exposure:**<br>• Get regular exposure to bright light during the day, especially in the morning. This helps regulate your circadian rhythm.<br>• Limit exposure to blue light from electronic devices in the evening, as it can suppress melatonin production and make it harder to fall asleep.<br>**Manage Stress:**<br>• Chronic stress can significantly impact sleep quality. Practice relaxation techniques like deep breathing, meditation, or yoga to manage stress levels.<br>**If you continue to experience sleep problems after making these changes, consult a doctor to rule out any underlying medical**

	**conditions.**<br><br>Here are some additional resources that you might find helpful:<br><br>• American Academy of Sleep Medicine: https://aasm.org/<br>• National Sleep Foundation: https://www.sleepfoundation.org/<br>• Mayo Clinic: https://www.mayoclinic.org/departments-centers/sleep-medicine/sections/overview/ovc-20407454<br><br>Remember, good sleep hygiene is essential for overall health and well-being. By making some lifestyle changes, you can improve your sleep quality and reduce your risk of future health problems.
My dad recently suffered a stroke. I think it's because he seldom exercises. What kinds of exercise can I do to decrease the risk of stroke?	I'm sorry to hear about your dad's stroke. Exercise is indeed a great way to reduce the risk of stroke, and it can be beneficial for people of all ages and fitness levels. Here are some exercises you can incorporate into your routine:<br><br>**Moderate-intensity Aerobic Exercise:** This is the foundation of a stroke-preventive exercise program. Aim for at least 150 minutes per week, or 30 minutes most days of the week. Examples include:

	• Brisk walking<br>• Swimming<br>• Biking<br>• Dancing<br><br>**Strength Training:** This helps build muscle mass, which improves overall health and can help regulate blood pressure. Aim for at least 2-3 sessions per week that target all major muscle groups. You can use bodyweight exercises, free weights, or resistance bands.<br><br>**Low-impact Exercises:** These are great options for beginners or those with limitations. Examples include:<br><br>• Walking<br>• Yoga<br>• Tai Chi<br>• Water aerobics<br><br>**Daily Activity:** Even small changes can make a big difference. Take the stairs instead of the elevator, park further away from your

	destination, or do some stretches or housework during commercial breaks while watching TV.<br><br>**Important Note:** Before starting any new exercise program, especially if you have a history of health problems, consult with your doctor. They can help you create a safe and effective routine based on your individual needs and fitness level.<br><br>Here are some resources that you may find helpful:<br><br>● American Heart Association: https://www.heart.org/en/healthy-living/fitness<br>● Centers for Disease Control and Prevention: https://www.cdc.gov/physical-activity/php/about/index.html
My dad recently suffered a stroke. I have a feeling it's because he enjoys eating fast foods. How can I reduce the risk of stroke by changing my diet?	I'm sorry to hear about your dad's stroke. You're right that diet can be a major factor in stroke risk. Here's how you can reduce your risk through diet:<br><br>● **Focus on fruits, vegetables, and whole grains:** Aim for at least 5 servings of fruits and vegetables daily. They're packed with vitamins, minerals, and fiber that can help lower blood pressure and cholesterol, both risk factors for stroke. Whole grains are also high in fiber and can help with blood sugar control, another important factor.<br>● **Choose healthy fats:** Replace unhealthy saturated and trans fats with healthy fats like those found in olive oil, avocados,

	nuts, and fatty fish (salmon, tuna). These fats can help improve cholesterol levels.<br>• **Limit processed foods and red meat:** Processed foods are often high in sodium, unhealthy fats, and can contribute to high blood pressure. Red meat consumption should be moderated, with lean cuts preferred.<br>• **Reduce added sugar:** Excess sugar can contribute to weight gain and diabetes, both risk factors for stroke.<br><br>Two dietary patterns that are particularly well-regarded for stroke prevention are the DASH (Dietary Approaches to Stop Hypertension) diet and the Mediterranean diet. Both emphasize fruits, vegetables, whole grains, healthy fats, and lean protein sources, while limiting unhealthy fats, salt, and added sugar. You can find more information about these diets online or by consulting a registered dietitian.<br><br>It's important to note that while diet plays a major role, it's not the only factor. Other lifestyle changes like regular exercise, maintaining a healthy weight, and managing stress can also significantly reduce stroke risk. If you have any underlying health conditions, talk to your doctor about how to manage them to further decrease your risk.

-Diagnosis

TOT	Responses
I've been having headaches quite often these days, especially on cold days. They seem to be getting worse with time. I'm really worried about my condition. Could it be a sign of stroke?	It's understandable to be worried about worsening headaches, especially with colder weather. While strokes can cause headaches, it's less likely compared to other causes.<br><br>Here's some general information on cold weather headaches:<br><br>● **Possible causes:** Changes in barometric pressure, reduced blood flow due to constricted blood vessels, dehydration from drier air.<br>● **Symptoms:** Throbbing pain, sinus pressure, feeling generally unwell.<br><br>Headaches caused by these factors are usually not severe and improve with home remedies.<br><br>However, it's important to get checked by a doctor to rule out other causes, especially since your headaches are worsening. Here are some warning signs to be aware of for stroke:<br><br>● Sudden and severe headache<br>● Weakness or numbness on one side of the body

	● Difficulty speaking or understanding speech<br>● Vision problems<br>● Dizziness or trouble walking<br><br>If you experience any of these in addition to headaches, seek immediate medical attention.<br><br>Here's what you can do in the meantime:<br><br>● **Track your headaches:** Keep a record of the frequency, severity, and any triggers you notice (cold weather, stress, etc.).<br>● **Stay hydrated:** Drink plenty of fluids throughout the day.<br>● **Dress warmly:** Cover your head and neck when going outside in the cold.<br>● **Over-the-counter pain relievers:** Consider pain relievers like ibuprofen or acetaminophen, but consult a doctor for long-term use.<br><br>I recommend scheduling a doctor's appointment to discuss your headaches in detail. They can properly diagnose the cause and recommend the best course of treatment.

I woke up this morning with my hand feeling really numb. I also think that my neck is sort of stiff too. Could these be the symptoms of a stroke?	Numbness in one hand and neck stiffness could be symptoms of a stroke, but it's also possible there's another explanation. Here's why:<br><br>● **Stroke symptoms can come on suddenly:** If your numbness and stiffness developed gradually over time, a stroke is less likely. Strokes typically cause sudden onset of symptoms.<br>● **Strokes often cause other symptoms:** In addition to numbness and stiffness, strokes often cause other neurological problems like weakness on one side of the body, facial drooping, slurred speech, or vision problems.<br><br>**Here are some other possible explanations for your symptoms:**<br><br>● **Cervical radiculopathy (pinched nerve):** This is a common condition where a nerve in your neck gets compressed, which can cause numbness and weakness in your arm and hand.<br>● **Poor sleep posture:** Sleeping in an awkward position can cause temporary numbness and stiffness.<br>● **Repetitive stress injury:** If you use your hand a lot for work or hobbies, you could have a repetitive stress injury that's causing numbness.<br><br>**It's important to get checked out by a doctor to determine the cause of your symptoms.** Early diagnosis and treatment of a

	stroke can significantly improve outcomes.<br><br>Here's what you can do:<br><br>● **If you're experiencing any other stroke symptoms, call emergency services immediately.**<br>● **If you're only experiencing numbness and stiffness, call your doctor to schedule an appointment.** They can do a physical exam and ask you questions about your medical history to help diagnose the cause.<br><br>In the meantime, you can try:<br><br>● **Applying heat to your neck:** This can help to loosen tight muscles.<br>● **Taking over-the-counter pain medication:** This can help to relieve pain and inflammation.<br>● **Gently stretching your neck:** Be careful not to overdo it.<br><br>I hope this information is helpful. Please let me know if you have any other questions.
I’ve been feeling dizzy for several	I understand your concern, dizziness and headaches lasting for weeks can be scary. While a stroke is a possibility, there are many

weeks already. To make matters worse, the headaches seem to be getting worse with time. Do you think I have a stroke?	other reasons you might be experiencing these symptoms.<br><br>Here are some more common causes of headaches and dizziness:<br><br>● Dehydration<br>● Migraines<br>● Medication side effects<br>● Inner ear infection<br>● Sinusitis<br>● Low blood sugar<br>● Stress or anxiety<br><br>**It is important to see a doctor to get a diagnosis and discuss treatment.** They can perform a physical exam and ask you about your medical history to rule out more serious causes.<br><br>In the meantime, here are some things you can do to feel better:

	- Drink plenty of fluids<br>- Get enough sleep<br>- Eat regular meals<br>- Manage stress<br><br>**If you experience any of the following symptoms, call emergency services immediately:**<br><br>- Sudden, severe headache<br>- Weakness or numbness in any part of your body<br>- Difficulty speaking or understanding speech<br>- Vision problems<br>- Loss of balance or coordination<br>- Seizures<br><br>I hope this information helps. Please don't hesitate to see a doctor to get the care you need.

<table>
<tr><td>My blood pressure has been higher than usual these days. So high that my head hurts even when I walk around. Could it be a stroke?</td><td>It's definitely concerning that your blood pressure is high and you're experiencing headaches. While headaches aren't a typical symptom of high blood pressure, they can occur, especially if your blood pressure spikes very high. However, there are other reasons for headaches and high blood pressure, and a stroke is a serious possibility that needs to be ruled out.

Here's what to do:

● See a doctor immediately. They can measure your blood pressure and determine if it's a hypertensive crisis (very high blood pressure that requires emergency attention). They can also investigate the cause of your high blood pressure and headache.
● In the meantime, rest. Avoid strenuous activity that could further elevate your blood pressure.

Here are some resources that you might find helpful:

● American Heart Association on high blood pressure: <u>What are the Signs and Symptoms of High Blood Pressure?</u>
● Medical News Today on high blood pressure and headaches: <u>Does high blood pressure cause headaches? Myths vs. facts</u></td></tr>
<tr><td>Ever since my dad</td><td>It's understandable that you'd be worried about your dad after he had a stroke. Strokes are serious medical events, but early</td></tr>
</table>

had a stroke, I've been constantly on the lookout for the signs. Can you tell me exactly what the symptoms are? Perhaps this will make me feel more at ease	detection and treatment can greatly improve the outcome. Here are the main symptoms of stroke to be aware of, using the acronym FAST:<br><br>● **F - Face Drooping** Does one side of your dad's face droop or feel numb? Ask him to smile. Does one side of his mouth droop down?<br>● **A - Arm Weakness** Can your dad raise both of his arms equally? Does one arm drift downward when he tries to hold both arms up?<br>● **S - Speech Difficulty** Is your dad slurring his words or having difficulty speaking? Can he understand what you are saying?<br>● **T - Time to Call 911** If you notice any of these signs, call 911 immediately. Time is critical when it comes to stroke treatment.<br><br>Here are some other stroke symptoms to be aware of:<br><br>● Sudden numbness or weakness of the face, arm, or leg on one side of the body<br>● Sudden confusion, trouble speaking or understanding speech<br>● Sudden trouble seeing in one or both eyes

	• Sudden trouble walking, dizziness, or loss of balance or coordination<br>• Sudden severe headache with no known cause<br><br>It's important to note that not everyone will experience all of these symptoms, and some symptoms may be more severe than others. However, if you notice any of these signs, it's important to call 911 immediately.<br><br>While knowing the signs of stroke can help you be prepared, it's important to remember that you can't control whether or not your dad will have another stroke. The best thing you can do is to encourage him to live a healthy lifestyle, which can help reduce his risk. This includes eating a healthy diet, exercising regularly, managing stress, and not smoking.<br><br>If you're feeling anxious about your dad's health, it may be helpful to talk to a therapist or counselor. They can teach you coping mechanisms for dealing with anxiety and help you develop a support system. You can also find support groups for stroke survivors and their families. Talking to others who have been through similar experiences can be very helpful.<br><br>Remember, you're not alone in this. There are many resources available to help you and your dad cope with the effects of stroke.

-Treatment

TOT	Responses

I've just been diagnosed with stroke. The doctor told me that there is severe blood plaque blockage. I think that this is way more serious than I thought. Are there any medications or treatments that can help?	I understand your concern. Severe blood plaque blockage, also known as atherosclerosis, can be serious. However, there are medications and treatments that can help.<br><br>**Medications:**<br><br>● **Clot Busters:** If you are experiencing a stroke caused by a blood clot, medications called thrombolytics or clot busters can be administered to dissolve the clot and restore blood flow to the brain. Alteplase (Activase) is the most common clot buster used for ischemic stroke. This medication is most effective if administered within 4.5 hours of stroke symptom onset [1].<br>● **Antiplatelet medications:** These medications help prevent platelets in your blood from clumping together and forming clots. Aspirin is a common antiplatelet medication that may be used to prevent future strokes. Other medications such as clopidogrel (Plavix) and dipyridamole (Persantine) may also be used [2].<br>● **Anticoagulants:** Also known as blood thinners, these medications help prevent blood clots from forming. Warfarin (Coumadin) is a common anticoagulant, but newer medications such as dabigatran (Pradaxa), rivaroxaban (Xarelto), and apixaban (Eliquis) may also be prescribed depending on your individual situation [2].

- **Cholesterol-lowering medications:** Statins are the most common type of medication used to lower cholesterol levels. By lowering cholesterol levels, statins can help prevent the buildup of plaque in your arteries [2].
- **Blood pressure medications:** High blood pressure is a major risk factor for stroke. If you have high blood pressure, your doctor will prescribe medication to help lower it. Medications such as ACE inhibitors, angiotensin II receptor blockers, beta-blockers, calcium channel blockers, and diuretics may all be used to control blood pressure [2].

Treatments:

- **Angioplasty and stenting:** This procedure involves inserting a thin tube with a balloon at the tip into the blocked artery. The balloon is then inflated to open the artery and a stent, a small wire mesh tube, may be inserted to help keep the artery open [2].
- **Carotid endarterectomy:** This is a surgical procedure to remove plaque buildup from the carotid arteries in your neck. Carotid endarterectomy may be recommended if you have severe narrowing of a carotid artery and are at high risk of stroke [2].

Lifestyle Changes:

	In addition to medications and treatments, there are several lifestyle changes that can help reduce your risk of stroke:<br><br>● **Healthy diet:** Eating a healthy diet that is low in saturated fat, cholesterol, and salt, and high in fruits, vegetables, and whole grains can help lower your blood pressure and cholesterol levels.<br>● **Regular exercise:** Aim for at least 30 minutes of moderate-intensity exercise most days of the week.<br>● **Maintain a healthy weight:** If you are overweight or obese, losing weight can help improve your blood pressure and cholesterol levels.<br>● **Quit smoking:** Smoking is a major risk factor for stroke. Quitting smoking is one of the best things you can do to improve your health.<br>● **Limit alcohol consumption:** Excessive alcohol consumption can increase your blood pressure and risk of stroke.<br><br>**Important Note:**<br><br>I am unable to provide any specific medical advice. The medications and treatments listed above are for informational purposes only. Please consult with your doctor to discuss the best course of treatment for you. Early intervention is essential for stroke treatment, so call emergency services immediately if you suspect a stroke. Signs of stroke include sudden weakness or numbness

	on one side of the body, slurred speech, trouble seeing, and dizziness.
I've just been diagnosed with acute stroke. Even though it's bad, I think that with the right treatment I will be able to get better. Do you have any suggestions on how this can be done?	I understand this must be a very scary time for you. A stroke is a serious medical condition, but with timely treatment, many people can make a significant recovery. Unfortunately, I cannot give specific medical advice. However, I can share some general information about acute stroke treatment:<br><br>● **Time is critical**. The sooner you receive treatment, the better your chances of recovery. If you suspect a stroke, call emergency services immediately.<br>● There are two main types of strokes: ischemic (caused by a blood clot) and hemorrhagic (caused by bleeding in the brain). The treatment for each type is different.<br>● **Ischemic stroke:** The most common treatment for ischemic stroke is a clot-busting medication called tissue plasminogen activator (tPA). This medication is most effective if given within 3-4.5 hours of stroke onset. In some cases, it may be given up to 4.5 hours after symptoms began.<br>● **Hemorrhagic stroke:** There is no clot-busting medication for hemorrhagic stroke. Treatment may involve medications to

	control blood pressure, surgery to remove the blood clot, or other procedures to reduce pressure in the brain.<br><br>Here are some resources that you may find helpful:<br><br>• **National Stroke Association:** https://www.stroke.org/en/<br>• **American Stroke Association:** https://www.stroke.org/en/<br>• **Mayo Clinic - Stroke:** https://www.mayoclinic.org/diseases-conditions/stroke/diagnosis-treatment/drc-20350119<br><br>Remember, I am not a substitute for professional medical advice. Your doctor will be able to create a personalized treatment plan based on the specific type of stroke you have and your overall health.
The doctor just prescribed some medication for stroke. However, I am really worried about the potential side effects. I don't want to end up worse than I already	I understand your worry about side effects, especially after a stroke. Here's what I can tell you:<br><br>• **Side effects:** Many stroke medications, especially blood thinners, can cause increased bleeding. Other potential side effects depend on the specific medication.<br>• **Talking to your doctor:** The best course of action is to discuss your concerns with your doctor. They can explain the specific

am. How long do I need to keep taking them?	risks and benefits of the medication you were prescribed considering your situation. They can also address any questions you have about side effects and how to manage them.<br>• **How long you need to take them:** This depends entirely on your individual situation and the type of medication. Stroke medications can be prescribed for short-term to break up a clot or long-term to prevent future strokes. Your doctor will determine the appropriate duration based on your needs.<br><br>**Here's what NOT to do:**<br><br>• Don't stop taking your medication without consulting your doctor. Stopping abruptly can be dangerous.<br>• Don't take someone else's medication for stroke. Stroke medications are prescribed based on individual needs.<br><br>It's important to have an open conversation with your doctor about your concerns. They can provide the best guidance to ensure you get the treatment you need while managing any potential side effects.
I’ve just been diagnosed with a severe stroke in the	I understand this must be a very scary time for you. A severe stroke with blood clots is a serious condition, but there is still hope

brain. From the CT scan, there seem to be blood clots everywhere. Is it really possible for the damaged area to recover?	for recovery.<br><br>Here's some important information:<br><br>• **Time is critical:** The sooner you receive treatment, the better the chances of minimizing brain damage and maximizing recovery.<br>• **Treatment options:** Medications to dissolve clots (thrombolysis) or procedures to remove them mechanically (thrombectomy) can significantly improve outcomes if administered quickly [3].<br>• **Recovery potential:** The brain has some plasticity, meaning it can reorganize itself to compensate for some damage. The extent of recovery depends on the severity and location of the stroke, but even with significant clots, improvement is possible [4].<br><br>**Here's what to do now:**<br><br>• Don't wait - Seek medical attention immediately. Every minute counts in stroke treatment.<br>• **Doctors will assess the situation and determine the best course of treatment.**

	**Additional Tips:**<br><br>● While waiting for medical help, someone can use the acronym FAST to recognize stroke symptoms:<br>○ **F**ace drooping<br>○ **A**rm weakness<br>○ **S**peech difficulty<br>○ **T**ime to call emergency services [1]<br><br>I cannot provide specific medical advice, but I hope this information is helpful. There is still a chance for recovery, and early intervention is crucial. Focus on getting the medical attention you need as soon as possible.
I am about to undergo surgery for stroke. This is my first time in the operating room and I am really scared. What will happen to	I understand how scary it is to be facing surgery for the first time, especially for something serious like a stroke. Here's a general idea of what to expect in the operating room, but remember, the specifics may vary depending on your unique situation and the type of stroke surgery you'll be undergoing.

me in there?	**Before Surgery:**<br><br>● You'll likely meet with the anesthesiologist to discuss your medical history and anesthesia options (general anesthesia where you're completely asleep, or regional anesthesia where you're numb in a specific area).<br>● Nurses will prepare you for surgery with an IV and medication. They may also shave the surgical area (head) if necessary.<br>● You'll be asked to sign consent forms after you've had a chance to ask any questions.<br><br>**In the Operating Room:**<br><br>● The OR will be a sterile environment with bright lights and a team of medical professionals including surgeons, anesthesiologists, nurses, and surgical technicians.<br>● You'll be transferred to the operating table and anesthesia will be administered.<br>● The surgical team will continuously monitor your vital signs throughout the procedure.<br>● The exact procedure will depend on the type of stroke you had. Here are two common surgeries:<br>○ **Carotid Endarterectomy:** This is for strokes caused by blocked arteries in the neck. The surgeon removes plaque buildup from the carotid artery to improve blood flow to the brain.

- **Hemicranectomy:** This is for strokes that cause severe swelling in the brain. The surgeon removes a portion of the skull to allow the brain room to swell without causing life-threatening pressure.

After Surgery:

- You'll be taken to the recovery room where nurses will monitor you as you wake up from anesthesia.
- Once you're stable, you'll be transferred to a hospital room for continued monitoring and recovery.

What to Ask Your Doctor:

The best way to manage your fear is to be informed. Here are some questions to ask your doctor to get a clearer picture of what to expect:

- What type of stroke surgery will I be undergoing?
- How long will the surgery take?
- What are the risks and complications associated with this surgery?
- What will recovery be like?

	• When can I go home?<br><br>Remember, it's completely normal to feel scared. Don't hesitate to talk to your doctor or nurses about your anxieties. They can provide support and answer any questions you may have.

-Recovery

TOT	Responses
I am worried that I will never recover from the stroke I had a few days ago. I am willing to try anything that may work, including rehabilitation and hyperbaric oxygen therapy.	It's completely understandable to feel worried after a stroke. The good news is that there are effective treatments available, and people can make significant progress in their recovery. Here's what you can do:<br><br>• **Rehabilitation is key:** As you mentioned, rehabilitation is crucial for stroke recovery. Talk to your doctor about starting a program as soon as possible. The first 3-4 months are considered the prime window for improvement [Johns Hopkins Medicine]. Rehabilitation can include physical, occupational, and speech therapy, depending on your needs.<br>• **Hyperbaric oxygen therapy:** While research is ongoing, hyperbaric oxygen therapy shows promise for some stroke patients. Discuss this with your doctor to see if it might be a good fit for your specific case.

	Here are some resources that you may find helpful:<br><br>● **American Stroke Association:** https://www.stroke.org/en/<br>● **National Institute of Neurological Disorders and Stroke:** https://www.ninds.nih.gov/<br><br>Remember, recovery is a journey, and it takes time and dedication. Focus on your progress, no matter how small it may seem.<br><br>There's also great support available from stroke survivor communities online and in-person. Don't hesitate to reach out for encouragement and share your experiences with others who understand.
I am worried that I will never recover from the stroke I had a few days ago. I am willing to try anything that may work, including rehabilitation and intravenous laser irradiation of blood.	I understand your worry and frustration after a stroke. It's completely normal to feel that way. The good news is that stroke recovery is possible, and many people make significant progress with rehabilitation.<br><br>Here's what you can do:<br><br>● **Rehabilitation is key:** Stroke rehabilitation is a well-established and highly effective treatment for improving function and regaining independence after a stroke. It typically involves a team of specialists like physiotherapists, occupational

	therapists and speech therapists who will create a personalized plan to address your specific needs.<br>• **Intravenous laser irradiation of blood (ILIB):** While there have been some studies suggesting benefits of ILIB for stroke recovery, the evidence is still limited. Larger and more robust studies are needed to confirm its effectiveness.<br><br>Here are some resources that you might find helpful:<br><br>• **American Stroke Association:** This website has a wealth of information on stroke recovery, including different types of rehabilitation and what to expect https://www.stroke.org/en/<br>• **National Institute of Neurological Disorders and Stroke:** This website provides information on stroke recovery research and clinical trials https://www.ninds.nih.gov/<br><br>**It is important to discuss any new treatments, including ILIB, with your doctor.** They can help you weigh the risks and benefits and decide if it's right for you.<br><br>Finally, remember that recovery after stroke takes time. Be patient with yourself and celebrate even small improvements.

I am worried that I will never recover from the stroke I had a few days ago. I am willing to try anything that may work, including rehabilitation and transcranial magnetic stimulation.	It's completely understandable to feel worried after a stroke, especially so soon after the event. Recovery is absolutely possible, and it's great that you're already looking into rehabilitation and transcranial magnetic stimulation (TMS). These are both well-regarded approaches to stroke recovery.<br><br>Here's some information that might be helpful:<br><br>• **Stroke Recovery Timeline:** While it varies by person, the first 3 months are considered the prime window for significant improvement https://www.hopkinsmedicine.org/health/conditions-and-diseases/stroke/stroke-recovery-timeline. This is a good time to be proactive with therapy.<br>• **Rehabilitation:** Rehabilitation is a cornerstone of stroke recovery. A team of specialists will assess your needs and create a personalized plan to help you regain function https://www.mayoclinic.org/diseases-conditions/stroke/in-depth/stroke-rehabilitation/art-20045172.<br>• **Transcranial Magnetic Stimulation (TMS):** TMS is a developing therapy that uses magnetic pulses to stimulate brain cells. While research is ongoing, TMS shows promise for improving motor function after stroke https://pubmed.ncbi.nlm.nih.gov/27394378/.

	**Here are some next steps you can take:**<br><br>● **Talk to your doctor:** Discuss your concerns about recovery and your interest in rehabilitation and TMS. They can advise you on the best course of treatment for your specific situation.<br>● **Stroke support groups:** Connecting with other stroke survivors can provide emotional support and helpful information during your recovery https://www.stroke.org.uk/stroke/support.<br><br>Remember, recovery from stroke takes time and dedication. It's important to stay positive and focus on the progress you can make. There are also resources available to help you manage the emotional impact of stroke.
How useful is rehabilitation is for stroke patients? I don't really think that some simple exercises can really help me recover. Exactly what kind of assistance does it	Stroke rehabilitation is actually very beneficial for patients, and it can go far beyond simple exercises. It's a comprehensive program designed to help you regain as much independence and quality of life as possible after a stroke. Here's why it's so useful:<br><br>● **Relearn and rebuild skills:** The brain has plasticity, which means it can reorganize itself after a stroke. Rehab helps retrain your brain to compensate for damaged areas and strengthen existing connections. This can include relearning movement,

provide?	speech, swallowing, and daily living tasks.<br>• **Many types of therapy:** Rehab isn't just about physical exercise. It often includes occupational therapy to practice daily tasks like dressing and bathing, speech therapy to improve communication, and cognitive therapy to address memory and thinking problems.<br>• **Improve emotional well-being:** Stroke can be emotionally challenging. Rehabilitation can include support groups and therapy to help you cope with anxiety, depression, and other emotional effects.<br><br>While it may seem like simple exercises at first, they are designed to gradually increase complexity and challenge your brain to rebuild lost connections. Over time, these exercises can lead to significant improvements in your abilities.<br><br>Here are some resources you can look at for more info:<br>• Stroke Association<br>• Mayo Clinic
I was recently discharged from the	Unfortunately, there's no one-size-fits-all answer to how long stroke rehabilitation lasts. It depends on several factors, including:

hospital after being treated for stroke. Unfortunately, I need to go back for rehabilitation each week. I have a feeling that it will take some time to recover. Can you tell me how long it will last?	• **Stroke severity:** More severe strokes tend to require longer rehabilitation times.<br>• **Time to treatment:** Strokes treated quickly often have better recovery outcomes.<br>• **Your overall health:** Pre-existing health conditions can impact recovery.<br>• **Rehabilitation intensity:** More intensive therapy programs can lead to faster progress.<br><br>However, there are some general guidelines:<br><br>• **The first 3 months:** This is considered a critical window for rehabilitation. Most patients see significant progress during this time [NIH (.gov) critical time window for rehabilitation after a stroke].<br>• **6 months and beyond:** Recovery continues, but at a slower pace. Many patients reach a plateau around this time [Mayo Clinic stroke rehabilitation].<br>• **Long-term:** It's important to note that some improvements can even be seen 12-18 months after a stroke, and continued therapy can be beneficial [Johns Hopkins Medicine stroke recovery timeline].<br><br>**The good news is that even with dedication and therapy, most stroke patients experience significant recovery.**

	Here are some resources that you might find helpful:<br><br>• The National Institute of Neurological Disorders and Stroke [National Institutes of Health (.gov) stroke ON National Institutes of Health (.gov) ninds.nih.gov]<br>• The American Stroke Association [stroke association ON American Stroke Association strokeassociation.org]<br><br>Remember, it's important to discuss your specific recovery timeline with your doctor or therapist. They can tailor a rehabilitation plan to your individual needs and give you a more accurate idea of how long it might take.
